# Understanding the health and well-being impacts and implementation barriers and facilitators of legally-mandated non-custodial drug and alcohol treatment for justice-involved adults: a qualitative evidence synthesis

**DOI:** 10.1186/s40352-025-00361-5

**Published:** 2025-10-01

**Authors:** Emma Fiona France, Louise Hoyle, Pauline Campbell, Hilda Bissozo Hernandez, Julie Cowie, Candida Fenton, Hannah Carver, Catriona Connell, Joshua Dumbrell, Rosie Hill, Fiona Blacklaw, NIHR Evidence Synthesis Scotland Initiative NESSIE, Bridget Davis

**Affiliations:** 1 University of Stirling, Stirling, UK; 2 National Institute for Health and Care Research Evidence Synthesis Scotland Initiative (NESSIE), Usher Institute, University of Edinburgh, Edinburgh, UK; 3 Glasgow Caledonian University, Glasgow, UK

**Keywords:** Justice-involved adults, Offenders, Drug treatment court, Treatment orders, Non-custodial sentences, Systematic review, Qualitative evidence synthesis, Framework synthesis, Health outcomes, Substance use

## Abstract

**Background:**

Non-custodial judicial treatment orders aim to reduce recidivism for justice-involved people with drug and/or alcohol use problems, but health and well-being impacts are not understood. We conducted the first qualitative evidence synthesis to explore the perceived impacts on health and well-being of treatment orders and the perceived barriers and facilitators to implementation from the perspectives of justice-involved adults, their family members/significant others, and staff delivering/ mandating the treatment.

**Design:**

We searched 14 bibliographic databases (31/10/2023-07/11/2023) and conducted supplementary searches to identify qualitative evidence. Two reviewers appraised methodological limitations using CASP and assessed confidence in review findings using GRADE-CERQual. We used framework synthesis to synthesise evidence. We integrated synthesised findings with results of a complementary quantitative review investigating health and well-being effects of treatment orders.

**Results:**

We synthesised 25 studies (29 reports); 22/29 reports had moderate or high methodological limitations. Most studies (*n* = 20) focused on USA drug courts; none focused on alcohol interventions. Only three studies had health and well-being as their main focus. No studies involved family members. Only one study reported a theory of how treatment orders might impact health. GRADE-CERQual assessments of 13 findings were high (*n* = 7/13), moderate (*n* = 4/13), or low (*n* = 2/13) confidence. Justice-involved adults perceived treatment orders to reduce mortality/morbidity risk, improve sense of self and coping with emotions, to result in feeling healthier, but also to exacerbate trauma and increase stress. Coerced treatment was perceived to interfere with “therapeutic change,” nonetheless it was often perceived to reduce, cease and/or stabilise illicit drug use. Justice-involved adults’ challenging life circumstances were an important barrier to reducing/ ceasing substance use. Abstinence-based approaches were common but abstinence may be unrealistic. Intervention effectiveness trials rarely measured relational outcomes of importance to justice-involved adults e.g., impacts on their children, or health outcomes.

**Conclusions:**

High-quality qualitative studies are urgently needed on the health impacts of diverse treatments orders. Treatment orders should emphasise harm-reduction treatment approaches and address participants’ healthcare and social needs. Theories of how treatment orders work are needed. Unintended negative health consequences of treatment orders must be researched. Future trials should measure and report health and relational outcomes. Study protocol registration: [CRD42023484923]. The National Institute for Health and Care Research (NIHR) Evidence Synthesis Programme (Grant: NIHR153425, project number NIHR162046) funded this study.

**Supplementary Information:**

The online version contains supplementary material available at 10.1186/s40352-025-00361-5.

## Background

People who become involved in the criminal justice system, by being charged with, or convicted for an offence (referred to hereafter as “justice-involved people”), have an extremely high likelihood of having substance (drug and/or alcohol) use problems (Gallagher, 2014; SAMHSA, 2021). For instance, in 2021 in the United States, 47.5% of adults aged 18 years or older who were on state or federal probation had diagnosed substance use problems, compared to only 17.3% of adults who were not involved in the criminal justice system (SAMHSA, 2023). In England in 2021–2022, around 45,000 adults in prisons and secure settings were undergoing alcohol and drug treatment (Official Statistics, 2023).

Adults involved in the justice system are overrepresented in deaths related to alcohol and drugs, for example, in England and Wales from 2011 to 2021 drug-related deaths in offenders who were supervised in the community by the probation service were over 16 times greater than the general population (Office of National Statistics, 2023). The health consequences of substance use are extensive including over 200 related conditions such as hepatitis C, AIDS/HIV, pulmonary, cardiovascular and liver diseases (United Nations, 2022).

Short-term custodial sentences have been strongly criticised as an ineffective means of rehabilitation for justice-involved people with substance use problems because they fail to address complex underlying issues such as experiences of trauma and victimization, poor mental health, homelessness, or housing insecurity (Eaton & Mews, 2019; Scottish Government, 2023; Trebilcock, 2011; Wermink et al., 2023). Incarceration does little to reduce drug use and criminal recidivism (DeMatteo et al., 2011). One approach to try to address substance use and related offending is through imposing mandatory substance use treatment as a condition of a non-custodial sentence instead of, or as well as, the traditional custodial criminal justice pathways.

Non-custodial judicial treatment orders started in the United States when the first drug court was established in Miami-Dade County, Florida, in 1989 and were subsequently adopted in various forms by other countries including Canada, Australia and the United Kingdom (UK) (Scottish Government, 2023). One influential underpinning theory was “therapeutic jurisprudence” - the impact of law and the legal process on the well-being of justice-involved people (Wexler & Winick, 1992) - which aims to promote rehabilitation, rather than just punishment. Another was the rationale that substance use increases the risk of reoffending, sometimes called the “drug-crime nexus” (Goldkamp, 2003). Mandatory rehabilitative interventions intended to reduce offending are likely to comprise a mix of components including mandatory substance use testing or continuous monitoring through electronic tagging; psychological/ behavioural interventions aimed at preventing relapse; other community-based interventions (e.g., mutual aid programmes like the 12-steps programmes); medication-assisted treatment (MAT) for opioid use; and/or attendance at specialist drug or alcohol courts (Baughman et al., 2019; Green & Rempel, 2012; Lindenfeld et al., 2022). Some interventions are aimed at harm reduction which might involve reducing alcohol consumption or illicit drug use, using a legal substitute like methadone, or using a safer method of drug administration, e.g. orally rather than injected (Klein, 2020).

In most cases a person must agree to comply with the treatment requirement before the order is made (Bright & Martire, 2013; Scottish Government, 2023). Failure to comply with the original requirement may result in legal penalties (e.g., probation, community service, or incarceration) (Bright & Martire, 2013; Scottish Government, 2023). The integrated monitoring and rehabilitation ultimately seek to address the underlying substance use in order to reduce recidivism (Bright & Martire, 2013; Hall & Lucke, 2010a, b; Scottish Government, 2023; Werb et al., 2016).

The effects of mandatory substance use treatment interventions have been evaluated mainly in relation to reoffending, not their health impacts (Bright & Martire, 2013; Hall & Lucke, 2010a, b; Scottish Government, 2023; Trood et al., 2021; Werb et al., 2016; Zanis et al., 2003). Therefore, there is a risk that justice-involved people with substance use problems are being mandated to engage with treatment and interventions that may have unintended negative impacts on their health (Perkins et al., 2022; Scottish Government, 2023). To our knowledge, there have been no systematic reviews of the health and well-being impacts of mandatory substance use treatment orders (Perry et al., 2009; Scottish Government, 2023). Therefore, we conducted two complementary systematic reviews: a review of intervention effects (henceforth referred to as the “quantitative review”) which aimed to determine the effects of non-custodial mandatory treatment on the health and quality of life of justice-involved people with substance use problems, reported elsewhere (Campbell et al., 2025), and a qualitative evidence synthesis. This article reports the qualitative evidence synthesis and its integration with the quantitative review.

### Aim and objectives

The aim was to explore the perceived impacts on health and well-being of non-custodial mandatory treatment for drug and/or alcohol use, and the perceived barriers and facilitators to treatment order implementation, from the perspectives of:

- Justice-involved adults mandated to participate in drug and/or alcohol treatment as part of their sentence, whether or not they comply with the orders.
- Their affected family members/ significant others, and.
- Staff / intervention providers (e.g., health and social care professionals, social workers, probation officers) involved in the mandated treatment.

### Review question

What are the perceived impacts on health and well-being of non-custodial judicial treatment orders for justice-involved adults and the perceived barriers and facilitators to treatment order implementation from the perspectives and experiences of adults mandated to participate in drug and/or alcohol treatment, their affected family members/significant others, and staff / intervention providers delivering or mandating the treatment?

## Methods

### Study design

We conducted two complementary evidence syntheses. Here we report the qualitative evidence synthesis using framework synthesis chosen because it suits applied research (Brunton et al., 2023). The reporting follows PRISMA (Preferred Reporting Items for Systematic Reviews and Meta-Analyses) (Page et al., 2021) and ENTREQ (Enhancing transparency in reporting the synthesis of qualitative research) guidelines (see Additional file 1) (Tong et al., 2012). The protocol was registered on PROSPERO: CRD42023484923. For amendments to the protocol, see Additional file 2. A list of abbreviations is given in Additional file 3.

### Patient and public involvement (PPI) and stakeholder involvement

We had PPI input from people with experience of treatment orders and affected family members/significant others and expert stakeholder involvement (JD, CC, HC, RH) throughout the review including during protocol development, review conduct and dissemination. PPI is important to ensure that evidence syntheses are meaningful and relevant to those who will be affected by the research (Pollock et al., 2019). Following good practice in PPI reporting, full details are reported using the ACTIVE (Pollock et al., 2019) and GRIPP2 (Staniszewska et al., 2017) reporting checklists (see Additional file 6).

### Inclusion criteria

We included primary qualitative studies on experiences and perceptions of treatment orders, and mixed-methods studies in which qualitative evidence was reported separately. Studies had to have used qualitative data collection and analysis methods. We included peer-reviewed publications and other published and unpublished texts, in English, with any publication date and full text available. We excluded systematic reviews and evidence syntheses. We planned to include studies that presented data from justice-involved juveniles and adults, if the data on adults were presented separately. We planned to include any non-custodial sentence that had mandatory substance use treatment within it. We excluded Family Alcohol and Drug Court orders because they involve voluntary treatment and are typically non-criminal courts.

### Identification of studies

#### Search strategy

An experienced Information Specialist (CF) devised and conducted a single search for both reviews and ran searches of 14 bibliographic databases between 31 October to 7 November 2023. The MEDLINE search (see Additional file 4) was adapted for other databases.

#### Electronic databases

We searched MEDLINE, Embase, CINAHL, PsycInfo, Web of Science, LexisPSL, Westlaw UK, National Criminal Justice Reference Service (NCJRS), ASSIA (Applied Social Science Index and Abstracts), IBSS (International Bibliography of Social Science), Policy Commons, Social Care Online, the World Health Organization International Clinical Trials Registry Platform (ICTRP) and ClinicalTrials.gov. We conducted forward and backward citation searches of trials included in the quantitative review to identify any linked qualitative studies.

### Data collection and analysis

#### Selection of studies

An information specialist (CF) removed duplicate records using Endnote software and then imported records into Covidence systematic review management software (Veritas Health Innovation, 2022). Two reviewers (PC, BD, EF, JC and/or LH) independently screened titles and abstracts and then full texts for inclusion. Disagreements were discussed, involving a third review author (CC, HC, JD) when necessary. Records excluded during full text screening are listed in a table, with reasons (see Additional file 5).

An exhaustive sample was not required because qualitative evidence synthesis aims for depth of understanding and insights and a large volume of data can impede in-depth analysis; therefore, we purposively sampled eligible studies (Ames et al., 2019; Benoot et al., 2016). We consulted with our Patient and Public Involvement (PPI) and stakeholder groups (for details, see Additional file 6) and referred to published guidance to develop the sampling criteria below (Noyes et al., 2018):

- A study linked to a trial in the quantitative review.
- The fit between the context (country, region and type of intervention e.g., drug court, treatment order, MAT, alcohol versus drugs focus) of qualitative studies and the context of trials in the quantitative review to facilitate integration of findings.
- Diversity of research participants e.g., justice-involved adults, legal staff, treatment providers, family members, peers/volunteers.
- Representing the views of minority or marginalised groups such as ethnic minorities, justice-involved women and justice-involved younger adults.
- Providing data on other equity issues of importance to our PPI and stakeholder groups, including (un)employment and housing/homelessness issues.
- Providing data on communication between different organisations/practitioners involved in delivering or mandating treatment orders.
- Alcohol and drugs focus.
- The fit of the study’s aim with our review aim.
- The volume of data in the study relevant to our review aim.
- The current relevance of the study data.

#### Data extraction and coding

One reviewer (BD, EF, HBH, or LH) extracted data on study, participant and intervention characteristics including design, aim, methods, setting, funder, conflicts of interest, substance use details and equity data (Cochrane Methods Equity, 2021). We used a standardised form within Covidence developed by the author team, piloted on five publications and revised. A second reviewer (BD or EF) checked all data extraction.

#### Qualitative data coding, analysis and synthesis

Where multiple included publications reported the same qualitative study but presented different findings, separate assessments and analyses of the data were conducted. People’s views, experiences, and preferences regarding an intervention can tell us how acceptable and feasible it is which are key considerations affecting its implementation (Yardley et al., 2015). Therefore, we analysed qualitative findings on views, experiences, and preferences relating to treatment orders.

We used framework synthesis involving: (1) familiarisation, (2) framework identification, (3) indexing, (4) charting and (5) mapping and interpretation (Brunton et al., 2023). In steps 1 and 2, we developed an initial deductive coding framework (Ritchie & Spencer, 1994) of thematic categories (see Additional file 7) based on familiarity with the review topic, the review aim, the included studies, and a theory-informed implementation framework for policy-maker decision making (The SURE Collaboration, 2011). In step 3, one reviewer coded qualitative findings from each included study using the coding framework in NVivo software (Veritas Health Innovation, 2022); a second reviewer independently checked coding of seven studies to ensure accuracy and consistency of coding. We modified the framework inductively to accommodate additional thematic categories. In step 4 we distilled coded data into charts by thematic category in Microsoft Excel differentiating between study participants, interventions, and geographical settings. In step 5, we further developed findings and thematic categories inductively from the data. The wider review team, topic experts, and our PPI group regularly discussed the data analysis and interpretation. We have presented the synthesised findings in a narrative organised under 12 overarching categories.

### Assessment of methodological limitations of included studies

We used the Critical Appraisal Skills Programme (CASP) for qualitative studies (CASP, 2018). Two reviewers (EF, BD, LH, and/or HBH) independently assessed methodological limitations according to nine domains: research aims, methodology, research design, recruitment strategy, data collection, reflexivity, ethics, data analysis, and findings. We then made an overall judgement of low, moderate or high methodological limitations. Disagreements were resolved through discussion, involving a third reviewer when necessary. CASP results informed GRADE-CERQual (Confidence in the Evidence from Reviews of Qualitative research) judgements of how much confidence can be placed in our synthesised findings (Lewin et al., 2018).

### Assessment of the confidence in the evidence

Two reviewers (EF, BD, and/or LH) applied GRADE-CERQual (Lewin et al., 2018) to evaluate the overall confidence in the synthesised evidence for each key review finding based on:

- Methodological limitations: any concerns about the design or conduct of the primary studies that contributed evidence to an individual review finding (Munthe-Kaas et al., 2018).
- Coherence: how clear and well-supported the fit is between the data from the primary studies and a review finding (Colvin et al., 2018).
- Adequacy: the degree of richness and quantity of data contributing to a review finding (Glenton et al., 2018).
- Relevance: how applicable to the context (population, phenomenon of interest, setting) specified in the review question the data from the primary studies supporting a review finding are (Noyes, Booth, Lewin et al., 2018a, b).

We judged the confidence that each review finding is a reasonable representation of the phenomenon of interest as high, moderate, low, or very low (Lewin et al., 2018).

### Integrating the quantitative and qualitative reviews

We integrated our two complementary reviews during their design, including the review question formulation (Harden et al., 2018; Noyes et al., 2019), and integrated the findings during the synthesis using quantitative/qualitative data integration methods from Cochrane Qualitative and Implementation Methods group (Harden et al., 2018). Joint qualitative and quantitative review team membership with close weekly collaboration enabled us to establish a high level of coherence between the qualitative and quantitative evidence.

We planned to determine if the programme theories (i.e. how a complex intervention is thought to work) (Noyes et al., 2016) of interventions/trials included in the quantitative review matched research participants’ views and expectations. We also used a matrix approach adapted from prior reviews (for example (France et al., 2023; Munabi-Babigumira et al., 2017) to explore whether research participants’ views, preferences and desired outcomes, identified in the qualitative evidence synthesis as having the potential to affect treatment order implementation, were acknowledged or addressed in the trials.

## Results

### Results of the search

**Fig. 1:**
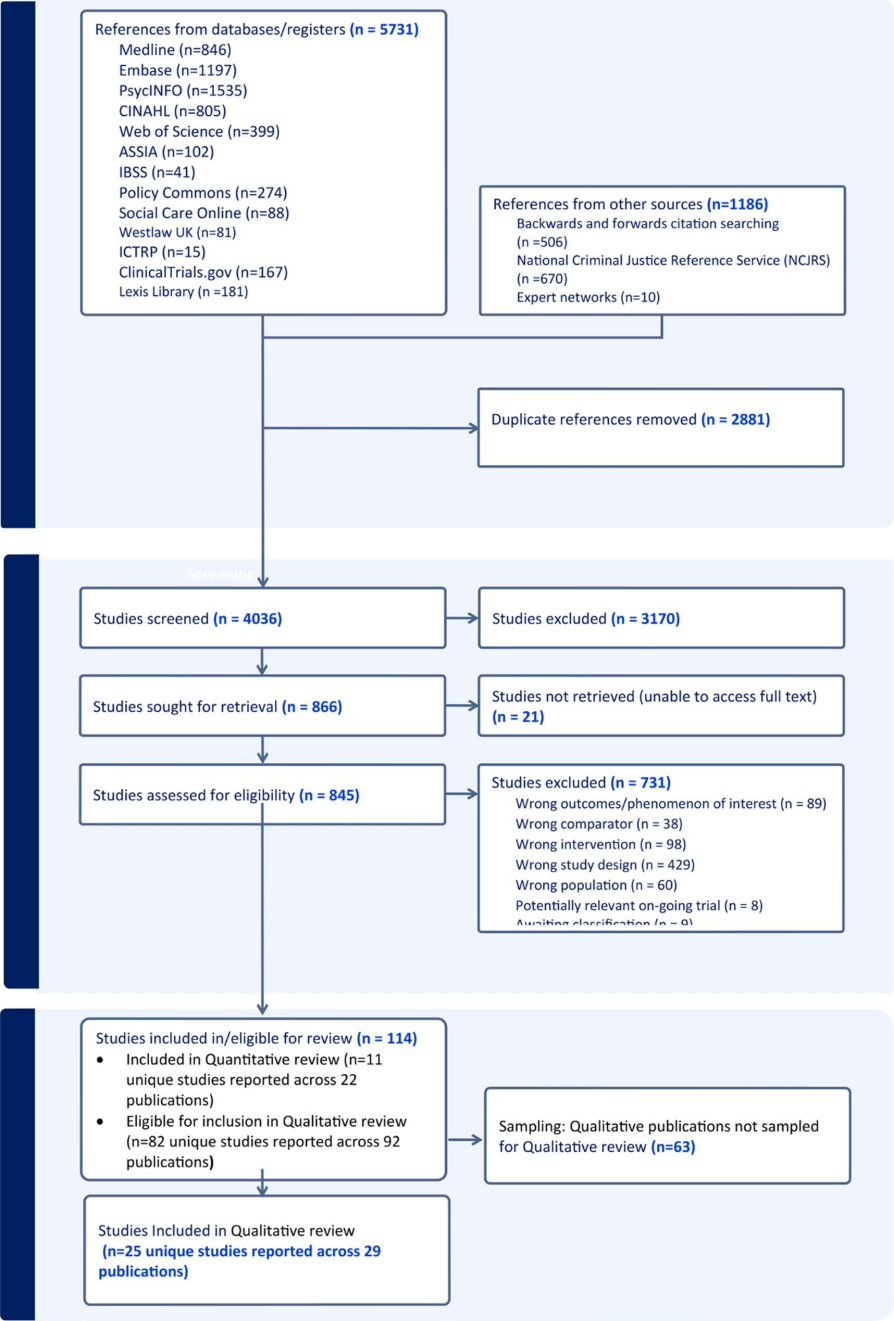
PRISMA

Our systematic searches identified 6,917 potential records, of which 82 qualitative or mixed qualitative-quantitative studies reported in 92 publications (listed in Additional file 8) met the eligibility criteria. Results of the search are summarised in the PRISMA flow diagram (Fig. 1). We excluded 731 studies. The main reasons for exclusion were wrong (ineligible) study design (*n* = 429), wrong intervention (*n* = 98) or wrong outcomes/ phenomena of interest (*n* = 89). The reasons for exclusion are reported in Fig. 1 and Additional file 5.

Eligible studies were published between 1998 and 2023 and all were conducted in high-income countries (The World Bank 2023). Most studies were carried out in the USA (*n* = 69 studies, 84%); five (6%) in Canada; five in England, United Kingdom (UK) (6%); one in Scotland, UK (1.5%); and one (1.5%) in each of Ireland, New Zealand and Australia. These studies represented the views of justice-involved people (*n* = 57 studies), of which 10 focused solely on women; judicial staff (*n* = 39 studies); and treatment providers (*n* = 31 studies); none included family members or significant others. Thirty-five studies focused on drug use only. Only three eligible qualitative studies focused on legally-mandated alcohol interventions e.g. a Driving While Intoxicated (DWI)/Driving Under the Influence (DUI) Court or programme (Engstrom, 2023; Narag et al., 2013; Osilla et al., 2017). However, 44 studies (53%) focused on justice-involved adults who used both drugs and alcohol. Only three studies (four publications) main focus was health and well-being (Garcia et al., 2019; Hamilton, 2019; Morse et al., 2014, 2015). For the table of study characteristics see Additional file 8.

### Results of sampling

We applied sampling criteria resulting in a sample of 29 publications published from 1998 to 2023 (72% were published 2010–2023) reporting 25 studies for inclusion in the synthesis. Three publications reported findings from a longitudinal evaluation of a drug court pilot conducted in Scotland, UK (Eley et al., 2002; McIvor, 2009; McIvor et al., 2006). Two publications reported the same cross-sectional focus group study in the USA (Morse et al., 2014, 2015) and two publications reported different findings from the same open-ended survey in the USA (Gallagher et al., 2017; Gallagher & Wahler, 2018).

We sampled and included the only eligible qualitative (mixed-methods) study related to a trial in the quantitative review (Harrell et al., 1998). Trials in the quantitative review all focused on “drug courts” and were conducted in the USA (Arizona, Baltimore, New York, North Carolina, Washington DC, ) or Australia (New South Wales). Therefore, we also sampled qualitative studies conducted in the USA (*n* = 17) and the only eligible qualitative study conducted in Australia (*n* = 1); we included studies from the same regions/states where possible. We also sampled qualitative studies from other high-income countries (UK and Canada) with interventions based on the USA drug court model. We included studies of drug courts, Drug Treatment and Testing Orders, and treatment orders. We did not include any of the three relevant eligible studies on legally-mandated alcohol interventions because they had a poor fit with our review aim (focusing mainly on recidivism) (Engstrom, 2023) or had insufficient relevant findings (Narag et al., 2013; Osilla et al., 2017).

### Key characteristics of included studies

Seventeen of the 25 included studies were conducted in the USA, four in England, two in Canada, one in Australia, one in Scotland. Included studies represented the views and experiences of 1,389 (range 5-316) research participants: 804 justice-involved people, 262 judicial staff, and 323 treatment provider staff (where reported). Most studies collected data via qualitative interviews only (13 studies), interviews plus focus groups (4 studies), or interviews plus observation (3 studies). Other data collection methods included secondary analysis of interviews, drug court records, and surveys.

### Types of intervention and substances

Most studies focused on drug court treatment programmes (20 studies) including one on The Substance Abuse and Crime Prevention Act of 2000, otherwise known as the Proposition 36 programme, in California, USA (Bevli, 2018). Three studies specifically focused on Drug Treatment and Testing Orders (Kouimtsidis et al., 2007; Powell, 2012; Ricketts et al., 2005). One focused on a residential treatment programme for pregnant women (Salzman, 2023), and one on MAT under community supervision (Kennedy-Hendricks et al., 2021). Fifteen studies focused on drug use only, ten studies on drug and alcohol use, and none on only alcohol use. The drugs being used by research participants were reported in eight studies and included cocaine/crack cocaine, cannabis, heroin, benzodiazepines, methamphetamine, ecstasy, psychedelics, prescription drugs and other opioids such as fentanyl.

### Theories or rationales for how treatment orders work

In their intervention descriptions, 16 studies did not report an underpinning theory or rationale for how the treatment orders were thought to work in general; only one study in Vancouver, Canada, referred to health in their rationale (Garcia et al., 2019). Where stated, theories or rationales tended to lack detail. They included that addressing drug use through mandatory treatment would reduce drug-related crime (a “drug-crime nexus” approach) (Eley et al., 2002; Kouimtsidis et al., 2007; McIvor, 2009; McIvor et al., 2006; Powell, 2012). Another underpinning theory was an abstinence-based treatment philosophy (Gallagher et al., 2019). Two studies referred to “legal leverage” (Garcia et al., 2019; Morse et al., 2015), which Garcia et al. explicitly linked to health: “the use of legal authority to promote treatment adherence and good health and well-being” (Garcia et al., 2019, p. 4). One study referred to both therapeutic jurisprudence and the drug-crime nexus (Salzman, 2023).

### Treatment providers

Twenty-two included studies did not report who delivered the treatment. Where specified, providers included multidisciplinary teams of social workers, drug/alcohol counsellors and medical staff (Eley et al., 2002; McIvor, 2009; McIvor et al., 2006); drug/alcohol counsellors (Fulkerson et al., 2012; Kouimtsidis et al., 2007) and consultant psychiatrists (Kouimtsidis et al., 2007); UK National Health Service (NHS) drug treatment services (Powell, 2012); and addiction psychologists (Kerr et al., 2011). Some studies referred only to “treatment providers” e.g., (Fischer et al., 2007; Harrell et al., 1998). See the table of eligible studies in Additional file 8 and the table of included studies in Additional file 9 for further details of study characteristics.

### Demographics of justice-involved participants in included studies

Justice-involved research participants were aged from 18 to 78 years old, with mean ages from 26 to 39 years (where reported). Nine studies did not report participants’ ages. The ethnic origins of justice-involved participants included Aboriginal/First Nations; Asian (not specified), Hispanic/Latinx; Black African, African-Caribbean, or African American; White; Mixed; Pacific Islander or ‘other’. Eight studies did not report race/ethnicity. The number of women versus men was not reported in six studies. In studies reporting sex, most justice-involved participants were men (*n* = 571) and only 172 were women. Six included studies focused only on justice-involved women.

Equity data were poorly reported. No studies reported justice-involved participants’ sexual orientation. Very few studies reported participants’ parental status (*n* = 7 studies), level of education (*n* = 6), work/education status (*n* = 4), living arrangements (*n* = 3), housing status (*n* = 2), duration of substance use problems (*n* = 2), co-morbid physical health conditions (*n* = 2) or mental health conditions including post-traumatic stress disorder/trauma and other adverse life experiences such as childhood abuse or domestic abuse (*n* = 6), or the seriousness/type of crime (*n* = 8). Gender identity was slightly better reported (*n* = 15 studies) but only as a binary male/female distinction.

### Methodological limitations of included studies

**Fig. 2:**
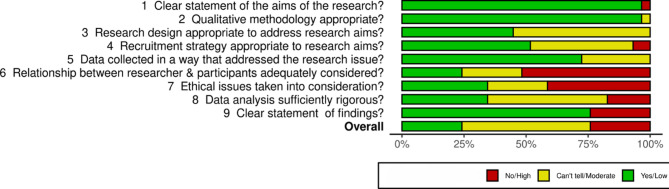
Summary of Methodological Limitations

For the overall assessment of methodological limitations in the sample of 25 included studies across 29 publications, almost one quarter of study reports (24%, *n* = 7) were judged to have minor methodological limitations, over half (52%, *n* = 15) had moderate limitations and almost one quarter (24%, *n* = 7) had high limitations. Figure 2 presents the summary of methodological limitations created using Robvis web app (McGuinness & Higgins, 2021). For the full CASP assessments, see Additional file 10. The most common methodological limitations were a lack of consideration of the researcher-participant relationship, ethical issues, lack of a clear statement of findings, and low rigour of data analysis. In many domains, such as the rationale for the research design, appropriateness of the recruitment strategy, data collection methods, and/or rigour of the data analysis, assessing limitations was hampered because relevant information was not reported.

### Findings

We developed, and present below, synthesis findings organised under 12 overarching categories, two of which are grouped under the heading “impacts of treatment orders,” eight under “facilitators and barriers to treatment order implementation” and two under “equity issues.” There were very limited data about perceived and experienced impacts of treatment orders on health and well-being including impacts on substance use, but more data on the perceived barriers and facilitators to treatment order implementation that might influence substance use. The studies and data that contributed to each category are described in Additional file 11.

### Impacts of treatment orders

#### Impact of treatment orders on health and well-being

The sparse data related more to mental and emotional health than to physical health and mainly reported the views of justice-involved adults. Some providers addressed mental health problems in treatment and this was perceived to have a positive impact on substance use and coping with prior trauma (Morse et al., 2014). Justice-involved adults identified improvements to emotional well-being such as an improved sense of self (Moore et al., 2017) and greater awareness of and ability to handle their emotions as a result of treatment orders (Bates, 2009), as this justice-involved adult described:

I’m more in contact with myself. I feel now. I have feelings that I can work through. I actually have to let it out and work through it instead of just covering it up with drugs. It’s like, back then it was just a trying-to-fit-in thing. Now, it’s just, I don’t care if I fit in, but we’re all doing it together in drug court. I’m kind of my own person. I’ve changed too. I feel like I have pride now and I have more self-esteem. (Bates, 2009, p. 91).

Some mandated treatments did not address experiences of trauma, violence and abuse which contributed to substance use; moreover, time in prison to punish substance use relapse could compound trauma (Datchi & Ancis, 2017). Other unintended negative consequences of lengthy treatment orders were that they could negatively impact mental health due to the stress of competing demands of family, work and treatment (Hamilton, 2019). Loss of custody of one’s children due to participating in mandated treatment (e.g., because residential treatment disallowed children) or loss of parental rights due to jail time could have a serious adverse effect on parental mental health; the consequent psychological impacts also needed addressing (Datchi & Ancis, 2017; Fischer et al., 2007).

In Scotland, justice-involved adults reported feeling better physically and having improved health due to mandated treatment, with sheriffs noting their healthier appearance (Eley et al., 2002). In terms of perceived impacts on mortality, young justice-involved adults in a USA drug court feared relapse would result in their death (Moore et al., 2017). In a USA study, some pregnant women said mandated treatment saved their lives by getting them off drugs and reducing their exposure to incurable sexually-transmitted infections from sex work which had funded their illicit drug use (Salzman, 2023): “Amy: I would be a sad excuse for a person [if I had not been arrested]. I’d still be walking the streets; I’d probably have herpes or aids and I know I wouldn’t have my son.” (Salzman, 2023, p. 208). Staff (referred to only as “court staff”) expressed that combining substance use treatment with housing for women and their children helped provide stability to focus on their health (e.g., dental care, contraception) and recovery (Morse et al., 2015).

Referral and eligibility criteria for drug court could exclude certain groups, such as women involved in sex work, from any health benefits associated with mandated treatments because their crimes were dealt with via other parts of the legal system (Morse et al., 2015):

”A lot of our female clients are charged with prostitution and…have combined chemical dependency and mental health issues… [which have] failed to be addressed in the past… A lot of them don’t qualify for drug court. [They] can’t get into mental health court because prostitution is a misdemeanor, not a felony, so there’s no real appropriate avenue to send them.” (Court Staff) (Morse et al., 2015, p. 4).

This had an unintended negative impact on equity for women.

In terms of family members’ health, pregnant women’s babies were at risk from the effects of illicit drug use (Salzman, 2023). Some worried about the impacts of methadone on their baby (Salzman, 2023).

#### Impact of treatment orders on substance use

Given the known negative impacts of illicit drug use on people’s physical health, reducing, stabilising and/or ceasing drug use should have positive physical health impacts.

In drug courts in the USA, England and Scotland, some justice-involved adults and judicial staff perceived that mandated treatment could reduce and/or stabilise drug use for justice-involved adults (men, women and young adults) through offering structure, routine and daily occupation e.g., frequently attending court, probation appointments and counselling (Bates, 2009; Bevli, 2018; Eley et al., 2002; Francis & Abel, 2014; Gallagher et al., 2019a; Gallagher & Wahler, 2018; McIvor et al., 2006; Ricketts et al., 2005). Other possible aids were medical support including MAT and psychiatric treatment; peer support (for example, in group counselling and Alcoholics Anonymous (AA) and Narcotics Anonymous (NA) meetings); and providing people with the skills to cope and solve problems without substance use (Bates, 2009; Bevli, 2018; Eley et al., 2002; Gallagher, Nordberg et al., 2019; Gallagher & Wahler, 2018; Gallagher et al., 2019; Moore et al., 2017; Ricketts et al., 2005; Salzman, 2023). For instance, this young justice-involved man stated:

I’ve learned that I’m not perfect. Nothing happens overnight, you’ve got to want a new life. Drugs take over your life before you know it. You have to create goals, make them tangible, focus on them, and achieve them (Moore et al., 2017, p. 756).

Support of family and friends also helped people cease substance use while subject to orders (see section “Relationships with family and friends”). However, interactions with justice-involved peers could also undermine mandated treatment. For instance, in group counselling sessions as part of Drug Treatment and Testing Orders in England, some attendees were selling drugs and talking about substance use which triggered cravings; attendees’ lack of motivation to stop was demotivating for others (Powell, 2012). Similarly, living with other women who were in early-stage addiction treatment with frequent relapses could trigger relapse for some First Nations women in Canada (Schiff & Waegemakers Schiff, 2010).

Drug courts sometimes involved medical support including MAT which was viewed as important during justice-involved adults’ early stabilisation (McIvor et al., 2006). MAT was perceived to have varying degrees of success in reducing/stopping illicit substance use (Eley et al., 2002; Gallagher et al., 2019b; McIvor et al., 2006).

### Facilitators and barriers to treatment order implementation

#### Motivation to stop substance use

Judicial staff in the USA, court Sheriffs (Scottish judges) and many justice-involved adults believed that participants needed to be ready to change their substance use behaviours for a treatment order to work (Bevli, 2018; Datchi & Ancis, 2017; Eley et al., 2002; Fischer et al., 2007; Kouimtsidis et al., 2007; McIvor et al., 2006; Morse et al., 2014; Powell, 2012; Ricketts et al., 2005):

You know, it wasn’t about me going to prison, it was about me, if I was ready to accept this, be ready to do what they’re asking me to do, and learn to love [myself]. (Chelsea, 38, African American, married with children, addiction to crack cocaine) (Datchi & Ancis, 2017, p. 109).

There were no data on participants’ motivation regarding treatment providers’ views and few data from legal staff.

Motivating factors to be drug-free included seeing the harm their substance use caused to others (Fulkerson et al., 2012), and pregnancy and motherhood for pregnant women - although many continued, or resumed, drug use after the birth of their children (Salzman, 2023). However, many justice-involved adults highlighted that they agreed to attend drug court to avoid jail (Bevli, 2018; Eley et al., 2002; Harrell et al., 1998; Maddox, 2023; McIvor et al., 2006; Salzman, 2023). Many justice-involved adults undertook the treatment order solely due to legal coercion (Bates, 2009). Nonetheless, motivation to complete the treatment order could develop over time with encouragement from judicial staff (Bates, 2009; Francis & Abel, 2014; Gallagher, Nordberg et al., 2019; Kerr et al., 2011; McIvor et al., 2006) and through experiencing success at reducing/ceasing drug use (Moore et al., 2017; Powell, 2012). A barrier to changing substance use behaviours was the use of substances to cope with unaddressed mental health/ emotional problems, including bereavement and trauma (Bates, 2009; Datchi & Ancis, 2017; Moore et al., 2017).

#### Life context of justice-involved adults who use substances

The prior and current life context and circumstances of justice-involved adults was an important barrier to them reducing or ceasing illicit drug use when undertaking a treatment order. Justice-involved adults often reported poor mental health, such as depression, anxiety and trauma (Francis & Abel, 2014; Gallagher, Nordberg et al., 2019; Hamilton, 2019; Salzman, 2023), which was also recognised by some drug court judicial staff (Dickson-Gomez et al., 2022; Garcia et al., 2019; Murphy, 2011). Treatment providers highlighted the negative effects of mental health (Morse et al., 2014) and justice-involved adults cited negative effects of both mental and physical health on ability to engage in treatment (Hamilton, 2019). Most treatment orders excluded people with diagnosed severe mental illness, however, some studies included participants with commonly reported mental health problems such as depression and anxiety (Francis & Abel, 2014; Gallagher, Nordberg et al., 2019; Hamilton, 2019; Salzman, 2023). Some justice-involved adults wanted mental health treatment integrated in their mandated treatment (Maddox, 2023).

Many justice-involved adults, especially women and Canadian First Nation adults, had experienced abuse, trauma and/or intergenerational trauma in childhood and/or adulthood which they said had contributed to their substance use and offending; many had started using substances as children (Bates, 2009; Bevli, 2018; Datchi & Ancis, 2017; Fischer et al., 2007; Garcia et al., 2019; Maddox, 2023; Moore et al., 2017; Salzman, 2023) Abuse-related trauma made it difficult for participants to trust and thus engage with treatment providers (Morse et al., 2014).

Other issues which affected people’s ability to engage with mandated treatment included financial problems, often linked to unemployment, which led to difficulties paying for mandated treatment and drug court fees in the USA (Bates, 2009; Francis & Abel, 2014; Maddox, 2023; Salzman, 2023), and affording transport to treatment sessions (Bevli, 2018; Maddox, 2023; Morse et al., 2015; Powell, 2012). Drug courts or local counties in the USA sometimes helped to pay fees which enabled participation (Bevli, 2018; Fischer et al., 2007). For some justice-involved adults, the stress of being unemployed made it more difficult to complete the treatment order (Gallagher, Nordberg et al., 2019). Other barriers to engaging with and completing treatment orders were lack of childcare (Eley et al., 2002; Fischer et al., 2007), homelessness or insecure housing (Dickson-Gomez et al., 2022; Morse et al., 2014; Powell, 2012; Ricketts et al., 2005), and living in neighbourhoods where drug use was common which “triggered” their own drug use (Bevli, 2018; Maddox, 2023; Murphy, 2011;. SAMHSA, 2021; Schiff & Waegemakers Schiff, 2010). Participants reported that having a conviction made it harder to secure housing (Morse et al., 2014, 2015). Usually, a combination of factors affected engagement:

‘The client…[does] not show up for their appointments…Or they don’t have a birth certificate, or…they’re missing their social security card. So the onus has to be on the drug court participant and they…may be homeless. They may not have an alarm clock. They may not have transportation… it’s an evil cycle because they may be using. They can’t remember when their appointment is. And the case managers can’t pick them up.’ Court staff member (Morse et al., 2014, p. 7).

The data suggest that mandated treatment orders did not adequately address the trauma, challenging life circumstances and complex-needs of this population, which resulted in justice-involved adults often struggling to engage and comply with Orders.

#### Ideology and defining treatment success

Differences were apparent in the perceived underlying ideology or philosophy of the drug courts or programmes and staff. The ideology affected how people understood and regarded substance use problems, relapse, which treatments were seen as acceptable, views and perceptions of monitoring and sanctions, and how staff viewed the “success” of mandated treatment.

Mandated treatments in USA studies tended to adopt a more punitive approach favouring abstinence and with a low tolerance of relapse (Datchi & Ancis, 2017; Fischer et al., 2007; Murphy, 2011). In the UK, a more rehabilitative orientation was seen (Kerr et al., 2011; McIvor et al., 2006). However, conflicting philosophies (e.g., rehabilitative versus punitive, abstinence versus harm reduction) were sometimes apparent in the UK and USA, e.g., between legal staff versus community treatment providers (Dickson-Gomez et al., 2022; Eley et al., 2002; Kennedy-Hendricks et al., 2021; Kouimtsidis et al., 2007) and between staff and justice-involved adults; the latter subscribed to a harm-reduction approach rather than an abstinence-based (Gallagher et al., 2019) or religious approach (Fischer et al., 2007).

Sheriffs (who had received drug court training) in Scotland believed that success should not be too narrowly defined and might differ for each person (Eley et al., 2002; McIvor et al., 2006):

(…) It may be regarded as a success if we manage to keep somebody out of trouble and out of prison for a couple of years. It may be regarded as a success if we get someone who’s on methadone for the rest of their life. We have to have different measures of success beyond the absolute. (Sheriff). (McIvor et al., 2006, p. 73).

Relapse was expected and the treatment emphasis was on MAT, specifically methadone (McIvor et al., 2006). Treatment providers in England also preferred a more holistic view of success which took account of improvements in health, well-being, and the likelihood of employment, rather than just focusing on abstinence from drugs and offending (Powell, 2012). Accommodating initial relapses meant participants could continue the treatment order and were more likely to succeed at ceasing/reducing illicit drug use (McIvor et al., 2006).

#### Tension between punishment and therapeutic treatment

The tension between mandated treatment as a punishment as opposed to a therapeutic experience affected experiences of, and attitudes to, treatment orders, and the use and perceptions of monitoring and legal sanctions. A range of justice-involved participants in several studies (men, women, Hispanic adults, non-completers of treatment orders in the USA and Scotland) perceived that being coerced to accept the mandated treatment interfered with “therapeutic change” (addressing the underlying problems to facilitate recovery through therapy) (Bates, 2009; Bevli, 2018; Datchi & Ancis, 2017; McIvor et al., 2006). However, some justice-involved adults undergoing Drug Treatment and Testing Orders in England perceived that precisely because treatment was forced it gave them the opportunity to benefit from it (Kouimtsidis et al., 2007). Other justice-involved participants saw mandated treatment as a good opportunity to change drug use behaviours and to recover despite the coercion (Eley et al., 2002; Fischer et al., 2007; McIvor et al., 2006).

The ambiguity of therapy versus punishment was also apparent in legal staff and treatment providers’ accounts (Eley et al., 2002; Kouimtsidis et al., 2007; Murphy, 2011), e.g., they used a specific residential treatment in the USA as both a sanction and a clinical intervention (Murphy, 2011). In a study in England, treatment providers felt they put in a lot of effort to overcome the therapeutic barrier of forced treatment since participants should attend voluntarily (Kouimtsidis et al., 2007).

Intensive monitoring and sanctions tended to be perceived as helpful if they were *not* humiliating, excessive, or purely punitive (Datchi & Ancis, 2017; Fischer et al., 2007; McIvor et al., 2006; Murphy, 2011). The threat of imprisonment for non-compliance could encourage justice-involved adults to accept accountability for their behaviour (Fulkerson et al., 2012) but monitoring and sanctions were not enough to prevent rule breaking and promote active engagement in treatment (Fischer et al., 2007; Harrell et al., 1998). Legal coercion, monitoring and sanctions as part of a wider treatment and support programme helped some, but not all, justice-involved adults comply with treatment order requirements which ultimately could help reduce/ cease illicit drug use (Datchi & Ancis, 2017; Fischer et al., 2007; Maddox, 2023; McIvor et al., 2006).

#### Attitudes to treatments

Studies mainly reported the views of justice-involved adults and treatment provider staff with fewer data from legal staff regarding their attitudes to treatments. Treatment staff in the USA and Scotland perceived that judicial staff and community treatment providers might favour or oppose a particular treatment (Eley et al., 2002; Kennedy-Hendricks et al., 2021). For example, some USA providers seemed to oppose MAT or to prefer one medication, such as methadone rather than buprenorphine or naltrexone, which could exclude justice-involved adults who were using a particular medication (Kennedy-Hendricks et al., 2021). In Scotland in early 2000, where the main mandated treatment was methadone, some participants were encouraged to use methadone even when they preferred not to (Eley et al., 2002; McIvor et al., 2006). Treatment team staff felt that a bias towards MAT could potentially limit efficacy of treatment orders (Eley et al., 2002):

I don’t think the services we have available for clients has been appropriate to their needs, I don’t think it has been good enough. I think that we’ve definitely done a sterling job with regard to substitute prescribing, but I think that we too often perhaps go down that road when, if there was a really high quality abstinence based treatment plan available or even a residential treatment plan… (…) if there were alternative treatment providers on the abstinent-based side, we would have had more success with abstinent based treatment plans. (Eley et al., 2002, p. 40).

In the USA and UK, some legal staff, treatment team staff and justice-involved adults felt that a wider range and quality of treatments, not just MAT, should be offered for effective treatment; suggestions included psychosocial treatment (Gallagher et al., 2019b; Kennedy-Hendricks et al., 2021), residential rehabilitation provision (Eley et al., 2002), job skills training (Harrell et al., 1998), and incorporating people’s existing supports such as faith groups, hobbies and sport (Gallagher & Wahler, 2018). Justice-involved adults in England wanted a wider range of medications, such as minor tranquillisers, not just substitute opioids (Powell, 2012). Justice-involved adults desired treatments tailored to their individual needs, including housing, childcare, transportation, and therapy needs, to maximise their chances of compliance and success (Eley et al., 2002; Fischer et al., 2007; Maddox, 2023). Treatment team staff stated that tailoring treatment plans was important but did not always happen (McIvor et al., 2006).

#### Relationships between justice-involved adults and staff

In drug courts in the USA and Scotland and for Drug Treatment and Testing Orders in England, positive relationships between justice-involved adults and staff, particularly the judge or sheriff (in Scottish drug courts), were often seen as central to the sustainability and success of mandated treatment (Bates, 2009; Bevli, 2018; Eley et al., 2002; Fischer et al., 2007; Fulkerson et al., 2012; Gallagher, Nordberg et al., 2019; Kerr et al., 2011; Maddox, 2023; McIvor, 2009; Ricketts et al., 2005). The studies reported mainly the views of justice-involved adults, with scant views of drug court staff and treatment provider staff.

Participants highly valued and were motivated to succeed when staff treated them with acceptance, support, fairness, respect, care and compassion, which many had rarely experienced in their lives (Bates, 2009; Bevli, 2018; Eley et al., 2002; Fischer et al., 2007; Gallagher, Nordberg et al., 2019; Maddox, 2023; Ricketts et al., 2005): “He [the judge] is very compassionate, very understanding, and very knowledgeable about recovery. He has our best interests and our welfare at heart. (Sharon)” (Fischer et al., 2007, p. 708).

Some participants referred to the judge/sheriff as a key “parental” figure of importance, (Bates, 2009; Eley et al., 2002; McIvor, 2009). Seeing the same judge/sheriff repeatedly helped develop mutual trust, rapport and a relationship (Bates, 2009; Fulkerson et al., 2012; Kerr et al., 2011; McIvor, 2009). Sheriffs also acknowledged the importance of enabling change and perceived that direct, personal connection was “a means of enhancing participants’ commitment and motivation to change” (McIvor, 2009)(p. 40). However, justice-involved adults in the USA sometimes distrusted and had conflict with drug court staff, often stemming from previous negative judicial experiences (Bates, 2009; Fischer et al., 2007; Fulkerson et al., 2012).

Justice-involved adults also perceived the personal, caring approach of treatment team staff, especially their counsellor or case manager, as a major contributory factor to achieving success (Bevli, 2018; Francis & Abel, 2014; McIvor, 2009; Moore et al., 2017; Salzman, 2023): “I started getting connected to a counselor [sic.] and I felt he believed in me. I really wanted to stay in treatment” (Francis & Abel, 2014, p. 332). In Scottish and USA drug courts, some justice-involved adults reported lack of trust in and feeling stigmatised by the treatment team which often resulted in missed appointments and non-compliance with treatment order requirements, thus impacting on progress (McIvor et al., 2006; Morse et al., 2014).

#### Relationships with family and friends

In Canada, the USA, and Scotland, family connections and peer support from other drug court participants could provide the support, strength and motivation to help complete a treatment order (Bevli, 2018; Eley et al., 2002; Gallagher, Nordberg et al.,; Garcia et al., 2019; Maddox, 2023; McIvor et al., 2006; Moore et al., 2017; Morse et al., 2014; Ricketts et al., 2005). However, judicial legal staff (in Scotland, USA, Canada) and justice-involved participants (in the USA) highlighted that participants’ family influences, stressors and dynamics could influence a relapse, for example, parents who were unsupportive of taking methadone, a partner or friends who used drugs or alcohol, or experiencing domestic abuse (Bevli, 2018; Datchi & Ancis, 2017; Eley et al., 2002; Maddox, 2023; McIvor et al., 2006; Morse et al., 2014, 2015): “When you’re in a domestic violence situation… she’s moving from place to place to place to place to place to place so that this person doesn’t find her. (Court Staff member)” (Morse et al., 2015, p. 5). Judges in Canada often took measures, such as safety management plans, to ensure that individuals experiencing domestic abuse were protected which could assist compliance with a treatment order (Garcia et al., 2019).

Justice-involved participants, including non-completers, highlighted that attending drug court and treatment orders could aid with repairing family relationships that had been damaged by their past substance use (Bates, 2009; Eley et al., 2002; Fischer et al., 2007; Fulkerson et al., 2012; McIvor, 2009; McIvor et al., 2006; Moore et al., 2017): “Just now I have never been so close to my family since I was a young boy, since before I was on heroin. They see me trying.” (Eley et al., 2002, p. 73). No studies gave family members’ perspectives.

Many women within drug courts in Scotland and USA felt that treatment order requirements did not recognise their caring responsibilities and specific needs as mothers (see also section “Impact of treatment orders on health and well-being”) (Eley et al., 2002; Fischer et al., 2007; Hamilton, 2019; Harrell et al., 1998; Maddox, 2023; Morse et al., 2014, 2015; Salzman, 2023). In the USA, Datchi and Ancis (2017) identified trauma, relationships, and family roles as critical factors that may explain differences in outcomes between men and women. Nonetheless, some justice-involved men also raised the struggle to combine childcare with a treatment order (Hamilton, 2019; Harrell et al., 1998).

#### Organisational and community barriers and facilitators

In several UK and USA studies, heavy staff workloads and caseloads, under-resourcing of treatment teams, staff illness, and difficulties recruiting and retaining staff were problematic, particularly in newly-established drug courts (Eley et al., 2002; Kerr et al., 2011; Maddox, 2023; McIvor et al., 2006). Treatment providers perceived these issues, many of which could be resolved with additional resources, to adversely affect the quality of service (Eley et al., 2002; McIvor et al., 2006).

Another issue was staff training and skills. Sheriffs and court staff in Scotland and judges in a Canadian drug court had been well-trained and demonstrated understanding of the complexity of substance use and offending behaviour (Eley et al., 2002; Garcia et al., 2019). Justice-involved adults preferred counsellors who had lived experience of substance use problems because they understood the challenges of recovery, were more relatable and trustworthy (Fischer et al., 2007; Hamilton, 2019; Harrell et al., 1998) or preferred trained counsellors specialised in drug use (Fulkerson et al., 2012; Maddox, 2023): “I’m not sure that they know as much about drug addiction as they do alcoholism. I would rather they have a counselor *[sic]* that really understands.. really knows drug addiction.” (Fulkerson et al., 2012, p. 1307). Women wanted female counsellors for gender-sensitive counselling to deal better with issues of physical and sexual abuse and separation from children (Fischer et al., 2007).

Staff raised the importance of good, frequent communication and good working relationships with mutual professional respect for multi-disciplinary working (Eley et al., 2002; Kennedy-Hendricks et al., 2021; Kerr et al., 2011; Kouimtsidis et al., 2007; McIvor et al., 2006). Poor communication was seen to affect referrals to drug court; result in treatment order participants receiving inconsistent messages about the rules, their appointments, and the services (Eley et al., 2002; Kouimtsidis et al., 2007; McIvor et al., 2006); and hampered monitoring participants’ compliance and imposing sanctions (Kennedy-Hendricks et al., 2021).

Delays between being assessed and receiving treatment (e.g., due to delayed drug test results) caused problems because the participants were highly likely to continue to use illicit substances in breach of their Order (Eley et al., 2002; Maddox, 2023; McIvor et al., 2006; Ricketts et al., 2005). Delays in psychiatric treatment were perceived to contribute to relapse (Morse et al., 2014).

Community-level barriers were apparent. The availability of appropriate community treatment facilities/services was identified as problematic in some USA studies. Suitable services for women were hard to find, for example, ones that accepted pregnant women (Salzman, 2023), women with children (Fischer et al., 2007), were women-only (Maddox, 2023); provided integrated housing (Maddox, 2023; Schiff & Waegemakers Schiff, 2010) or comprehensive holistic services (Fischer et al., 2007). Many treatment providers did not offer MAT or only offered one type of substitute medication (Kennedy-Hendricks et al., 2021) or were not accepting new patients (Dickson-Gomez et al., 2022).

### Equity issues

#### Homelessness, housing, employment, and training

Housing and employment are important equity issues that can affect health, well-being and substance use. To support maintenance of substance use reduction/cessation, assistance with housing and employment was desirable. Finding housing after completing a treatment order was challenging unless the programme provided housing assistance and support, which USA residential treatment providers tended to give (Dickson-Gomez et al., 2022; Maddox, 2023; Morse et al., 2015; Salzman, 2023; Schiff & Waegemakers Schiff, 2010). Participants reported that having a conviction made it harder to secure housing (Morse et al., 2014, 2015).

Justice-involved adults welcomed or desired support for employment and training as part of the treatment order (Eley et al., 2002; Fischer et al., 2007; Gallagher, Nordberg et al., 2019). Some reported that drug courts helped them find employment, education, or training (Fischer et al., 2007; Francis & Abel, 2014; Gallagher, Nordberg et al., 2019; Moore et al., 2017; Salzman, 2023). However, attending drug court and the treatment order requirements could negatively impact on securing and/or maintaining employment; many found juggling competing demands difficult (Bevli, 2018; Fischer et al., 2007; Fulkerson et al., 2012; Gallagher, Nordberg et al., 2019; Hamilton, 2019; Harrell et al., 1998; Maddox, 2023; Powell, 2012; Sarmiento et al., 2019): “The 9:00 a.m. to 3:00 p.m. treatment program leaves no time to get a job—forces you back to the streets to sell drugs for money.” (Harrell et al., 1998, p. 73). It helped when there was flexibility to enable them to maintain employment (Powell, 2012). Furthermore, clashes between job responsibilities and the strict drug court schedules could cause missed treatment appointments (Francis & Abel, 2014). Sanctions which prevented participants from working concerned justice-involved adults: e.g., losing their job by being sanctioned to live in a recovery house (Murphy, 2011) or detained in prison (Datchi & Ancis, 2017).

#### Ethnicity/race

Despite including three studies (reported in four publications) with a focus on particular ethnic groups (Hispanic, African American, First Nation Canadian) and many studies including a range of races/ethnicities, there was a lack of data about the specific impacts of drug courts on those from ethnic minority backgrounds (Bevli, 2018; Gallagher, Nordberg et al., 2019; Gallagher & Wahler, 2018; Schiff & Waegemakers Schiff, 2010). Datchi and Ancis (2017) highlighted that to improve treatment outcomes, there needs to be consideration of race as well as gender and socio-economic status.

#### Summary of barriers and facilitators

**Fig. 3:**
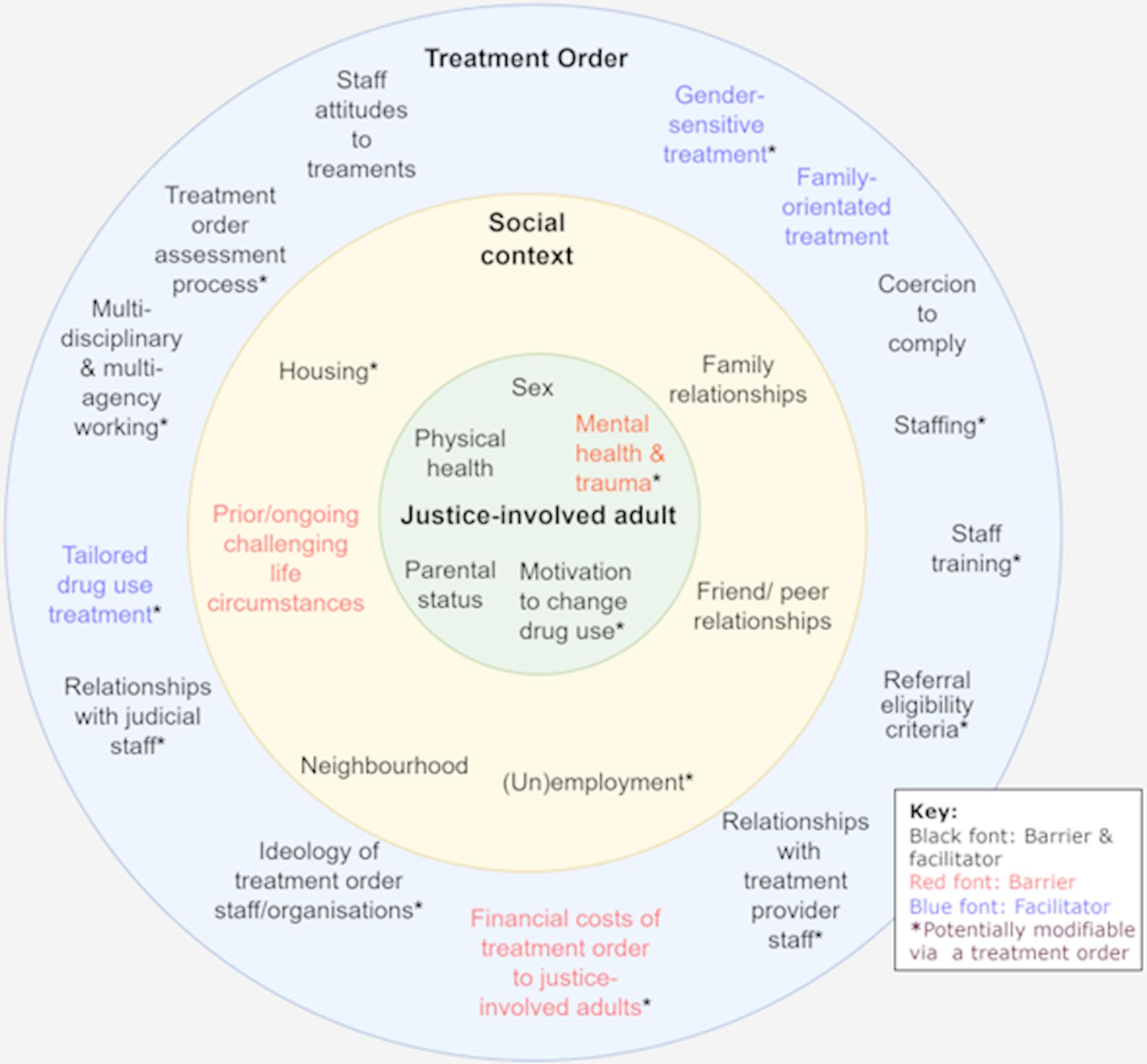
Treatment order barriers & facilitators affecting substance use

Our findings demonstrate barriers and facilitators at the individual, social and community, and organisational levels to successfully implementing mandated treatment orders to support cessation/reduction of substance use, especially illicit drug use, summarised in Fig. 3. These factors could influence health.

### Assessing confidence in the review findings

We have presented our application of GRADE-CERQual to the key synthesis findings in Summary of Qualitative Findings (see Additional file 12) and Evidence Profile tables (see Additional file 13) (Lewin et al., 2018; Lewin, Booth, Lewin et al., 2018a, b) created using the Interactive Summary of Qualitative Findings (iSoQ) tool (The Epistemonikos Foundation, Megan Wainwright Consulting, & The Norwegian Institute of Public Health for the GRADE-CERQual Project Group, 2022). There are 13 CERQual findings: seven assessed as high confidence, four moderate, two low and none very low confidence. All CERQual findings relating to the impacts of treatment orders on justice-involved adults’ health were low or moderate confidence. Other moderate confidence findings focused on justice-involved adults’ views of legal sanctions during a treatment order, the perceived impact of legal coercion on substance use, and the impact of positive relationships with judicial staff on engagement and compliance with treatment orders. High confidence findings related to relationships with family, peers and judicial staff; organisational barriers to treatment order implementation; and impacts of treatment orders on illicit drug use.

### Integration of the findings of the qualitative and quantitative reviews

There were no recognisable programme theories (specifically, describing the underpinning evidence for how the medical-judicial interventions were thought to work) in the trials included in the complementary quantitative review, therefore we could not integrate these with the qualitative synthesis findings. Three trials referred only to a “drug court model” (Harrell et al., 2001; Jones, 2013; MacDonald et al., 2007) and one to “therapeutic jurisprudence” (Gottfredson & Exum, 2002). In future trials, we would hope to see reporting of the evidence underpinning how the treatment order is thought to affect substance use problems, offending, and health and well-being, covering how the different components, e.g., counselling, court appearances, substance testing, and any sanctions, are thought to work together to achieve desired outcomes.

In the quantitative review we intended to analyse the primary outcomes of global functioning and quality of life and secondary outcomes of drug or alcohol use measures, severity of dependence, depression and anxiety, outcomes of family members/ significant others (e.g. depression, anxiety), and adverse events. Our framework synthesis identified several other key aspects of treatment orders that were important to justice-involved adults and staff: the needs of and impacts on justice-involved women may differ from men; parents of dependent children have particular needs; relationships with family, friends, peers and judicial and treatment staff play an important role in treatment order success; and treatment orders might impact family members, especially dependent children. Therefore, we focused this integration on whether the trials had:

- reported participants’ mental and physical health outcomes.
- reported family psychological/emotional and well-being outcomes.
- reported participants’ family relationship outcomes, such as family functioning, family support.
- compared participants with dependent children with non-parents.
- compared the outcomes of female and male justice-involved adults.
- reported aspects of the relationship with friends and peers and with judicial and treatment provider staff.

### Matrix analysis

The results of the matrix analysis (Table 1) show that, of the 11 trials, only one reported any physical health outcomes (number of days experiencing medical problems) (Harrell et al., 2001). Most trials (8/11) reported substance use outcomes (Deschenes et al., 1995; Desland & Batey, 1992; Harrell et al., 1998, 2001; Jones, 2013; MacDonald et al., 2007; Robertson, 2016; Rodriguez-Monguio et al., 2021). No trials reported well-being in terms of global functioning or quality of life outcomes, none reported anxiety outcomes, and only one reported a depression outcome (Green & Rempel, 2012). Two trials reported adverse life events: number of stressful life events (MacDonald et al., 2007) or sexual and physical victimisation (Green & Rempel, 2012). No trial reported family member or significant other health and well-being outcomes. Only three trials reported any family/significant other or friend/peer relationship outcomes, all of which were reported by the justice-involved adult: subjective functioning in family and social interactions (Robertson, 2016), family conflict (Green & Rempel, 2012; Harrell et al., 2001), family emotional support, family instrumental support (Green & Rempel, 2012), and peer conflict (Harrell et al., 2001).

All 11 trials included justice-involved women but most participants (3250/4643, 70%) were male; one trial was with women only (Harrell et al., 2001). One trial conducted a comparison of outcomes by sex but found no statistically significant differences for heroin use or employment (Desland & Batey, 1992). Two trials reported measures of the therapeutic relationship with judicial staff (meetings with parole officers, hearings or contacts with probation officers) (Deschenes et al., 1995; Gottfredson & Exum, 2002), but none reported frequency of contact or consistency of judge, or the relationship with friends and peers.

**Table 1:** Matrix integrating findings of the qualitative evidence synthesis with trials in the complementary quantitative review

	Important outcomes/variables to measure/report in trials						Important comparisons to make	
Trial in quantitative review	family psychological/ emotional & well-being	Family relationships	Friend/peer relationships	Justice-involved adults’ mental health	Justice-involved adults’ physical health	Relationship with judicial /treatment staff	Justice-involved adults with & without dependent children	Female & male justice-involved adults
Deschenes et al. (1995)	No	No	No	No	No	Yes	No	No
Desland and Batey (1992)	No	No	No	No	No	No	No	Yes
Festinger et al., (2016)	No	No	No	No	No	No	No	No
Green and Rempel (2012)	No	Yes	No	Yes	No	No	No	No
Gottfredson and Exum (2002)	No	No	No	No	No	Yes	No	No
Harrell et al. (1998)	No	No	No	No	No	No	No	No
Harrell et al. (2001)	No	Yes	No	No	Yes	No	No	No
Jones (2013)	No	No	No	No	No	No	No	No
MacDonald et al. (2007)	No	No	No	No	No	No	No	No
Robertson (2016)	No	Yes	No	No	No	No	No	No
Rodriguez-Monguio et al. (2021)	No	No	No	No	No	No	No	No

## Discussion

This qualitative evidence synthesis aimed to explore the perceived impacts on health and well-being of non-custodial judicial treatment orders and the perceived barriers and facilitators to treatment order implementation from the perspectives and experiences of justice-involved adults, their family members/significant others, and staff delivering or mandating the treatment. The findings of our synthesis reveal that justice-involved adults perceived treatment orders - intended to reduce offending through treating underlying substance use problems - to have some negative impacts on their mental health, such as stress from juggling mandated treatment with family and work responsibilities, and/or causing separation from one’s children. Phelps et al., (2022) also reported from a survey that the supervision demands of regular probation in the USA could negatively impact mental and physical health, such as poor relationships with probation officers causing stress, substituting illicit drugs with increased alcohol use, or no longer having illicit drugs as a coping mechanism for distress or pain; treatment orders may place even greater demands than probation on the health of justice-involved adults. We also found some reported positive impacts on mental health problems and physical health. However, overall there was a lack of evidence regarding perceived health and well-being impacts; all synthesis findings regarding health had low or moderate confidence CERQual assessments. Currently, there is a lack of quantitative evidence to corroborate how mandatory treatment affects health (Campbell et al., 2025); further research is needed.

We also identified that completing mandated treatment for substance use problems and offending was perceived to reduce and/or stabilise drug use for many justice-involved adults, despite coercion (high confidence CERQual finding). Quantitative research is required to support this finding. Furthermore, longitudinal research to follow-up those who have completed a treatment order is needed to investigate whether justice-involved adults maintain their behaviour change following a treatment order.

Our findings highlight that treatment orders need to address justice-involved adults’ pre-existing mental health problems, trauma and their highly challenging prior and ongoing life circumstances, which can interfere with their ability to engage and comply with treatment order requirements (low confidence CERQual finding). Failure to address these barriers may set people up to fail, exposing them to criminal sanction when they are unable to comply with a treatment order. Similar barriers have been identified for other substance using populations e.g., adults experiencing homelessness (O’Shaughnessy et al., 2024). Setiawan et al. (2024), in their synthesis of qualitative studies conducted internationally, found that for non-justice-involved individuals with substance use problems, psychological coping strategies needed to be included in treatments to address stress, negative emotions and triggering thoughts. An implication for practice is that for justice-involved adults who use illicit drugs to deal with mental health problems, coercing them to reduce or cease drug use without providing mental health support could have negative consequences on their well-being. Indeed, the UK National Institute for Health and Care Excellence (NICE) guidelines promote more coordinated care planning and service organisation across the criminal justice system for mental health (National Guideline Alliance UK, 2017).

Even outside the justice system, healthcare needs are poorly supported within substance use treatment settings (Savic et al., 2017), yet addressing physical health in substance use treatment can help prevent relapse (Manuel et al., 2017). Osborne’s international review of clinical practice guidelines for addressing the physical health of people attending alcohol and other drug treatment programmes highlighted that “greater consistency and specificity in the recommendations made for integrating physical health care within substance use treatment is needed” (Osborne et al., 2022, p. 1367). McKenna and Holtfreter (2021) have also recommended a need to address prior trauma, although in the traditional court context, to facilitate rehabilitation of justice-involved adults. To improve practice, judicial treatment orders need to integrate healthcare including mental health treatment and trauma-focused support.

Another of our key findings was that the influence of family, friends and peers can greatly influence treatment order progress e.g., by providing support or opposition (high confidence CERQual findings). Similarly, social support from family, friends and the wider community was seen as crucial in the recovery of individuals undergoing *voluntary* treatment for drug use (Farhoudian et al., 2022; Setiawan et al., 2024; Vigdal et al., 2022). Treatment providers should recognise the social and relational influences that may help or hinder progress and collaboratively plan for how to address this with the person undergoing mandatory treatment.

Justice-involved women highlighted that treatment orders could adversely affect relationships with their children because the treatment orders did not accommodate their caring responsibilities and needs, resulting in women being separated from their children and potentially losing child custody (high confidence CERQual finding). These issues also affect women undergoing voluntary treatment (Tsuda-McCaie & Kotera, 2022). In terms of improving practice, treatment orders need to develop and implement child- and family-focused services and family-inclusive practices. For instance, they could provide residential treatment facilities that accommodate children, support positive family and peer relationships that could facilitate reducing/ceasing illicit drug use, and aid the development or maintenance of supportive networks to sustain success post-treatment order. Accommodating parental needs and responsibilities would particularly benefit justice-involved women.

Our synthesis identified several factors unique to legally-mandated, as opposed to voluntary, drug use treatment which could facilitate or interfere with changing drug use behaviour: the treatment being coerced and the use of legal sanctions for non-compliance; the tension between punishment versus therapy; the relationship with judicial staff, especially the judge or sheriff; and multi-disciplinary, multi-agency working across judicial and treatment organisations. We explore the implications of each of these factors below.

Hybrid medical-legal interventions, such as treatment orders, face tensions between therapeutic treatment of substance use problems and coercion to participate, along with punishment for non-compliance. Treatment which is coerced might not be experienced as therapeutic (moderate confidence CERQual finding). Mandated treatment has both criminal justice and therapeutic goals (Freeman, 2001). This tension can affect the success of the intervention in changing substance use behaviour which might improve people’s health and reduce reoffending. Research and theories about motivation for change in other substance-using populations highlight that perceived choice of treatment goals is important to adherence and successful treatment (Miller, 2006; Paquette et al., 2022). Individually tailored goals could be incorporated into mandated treatment orders to enhance participants’ motivation and reduce reliance on coercion alone.

Research and theory suggest that some individual’s goals may be to reduce harms, in which case non-abstinence treatment may improve engagement, retention, and effectiveness (Miller, 2006; Paquette et al., 2022). Abstinence-based approaches tend to emphasise individual responsibility for substance use rather than the wider social, contextual and economic circumstances (Datchi & Ancis, 2017). Thus, abstinence may be an unrealistic or undesirable goal for many justice-involved adults with substance use problems. We found that perceptions of treatment order “success” differed depending on whether an abstinence-based or harm reduction model of substance use treatment was adopted. The sometimes-conflicting definitions of success need to be addressed when assessing the impact of treatment orders.

Our synthesis found that a positive relationship between justice-involved adults and judicial staff often developed, which encouraged and motivated participants to positively engage in the treatment order and address substance use problems (high confidence CERQual finding). For some people, a quasi-therapeutic relationship with drug court judges developed, consistent with a therapeutic jurisprudence perspective that court processes and “actors” can have therapeutic effects (Wexler & Winick, 1992). The relationship with treatment provider staff was also very important (moderate confidence CERQual finding), which is the case in voluntary treatment too (Setiawan et al., 2024). Consistency of judicial and treatment staff and developing trusting relationships should be a priority when implementing treatment orders.

Furthermore, we identified that the complexity of the multi-disciplinary, multi-agency working between diverse organisations with different approaches and ideologies about substance use problems and treatment could affect the quality of service and treatment (high confidence CERQual finding). All collaborating organisations should adopt a common underpinning ideology for treatment orders to guide their policies and practice and ensure consistent, coherent practices.

Therapeutic jurisprudence influenced the development of drug courts (Ahlin & Douds, 2020), but overall, the treatment orders as described in the synthesised studies did not appear to be underpinned by a comprehensive logic model or theory of change. Treatment orders as complex medical-legal interventions would benefit from further theoretical development with a focus on more holistic and harm-reduction approaches and should ensure that mandatory treatment orders consider health impacts. Those designing the interventions could draw on the socio-ecological model of behaviour change which specifies individual, interpersonal, organisational, community and societal factors (Bronfenbrenner, 1989). Theoretical development will be vital for informing the much-needed evaluative research in this area.

### Integration with complementary quantitative review

We integrated our synthesis with a complementary quantitative review which concluded that there is insufficient evidence to assess the effect of treatment orders on health and well-being (Campbell et al., 2025). The outcomes reported in the included trials from the quantitative review did not always match those of importance identified in our qualitative evidence synthesis. In addition, the trials lacked formal programme theories. Future trials should measure and report health and well-being outcomes for justice-involved adults and their families and compare men and women.

Since we started our synthesis, a new patient-centred core outcome set for “substance-related and addictive disorders” was published based on a consensus study with 26 experts but only five people with lived experience (Black et al., 2024). Their seven core outcome domains only include those relating to the individual: “frequency and quantity of addictive behaviour, symptom burden, health-related quality of life, global functioning, psychosocial functioning, and overall physical and mental health and wellbeing” (Black et al., 2024, p. 2). Our synthesis, that represents the views of justice-involved adults who use substances and judicial and treatment provider staff, has highlighted the importance of outcomes related to the wider social environment, such as family relationships, roles and social support; peer relationships; trauma and negative life events including domestic violence; and the process/experience, e.g., the therapeutic alliance or treatment experience. Trials should also measure these outcomes.

### Limitations

Syntheses are limited by the available relevant primary studies. We identified important gaps in the evidence which could limit the transferability of findings to other populations and settings. There was a lack of data on the perceived impact of treatment orders on justice-involved adults’ health and well-being: only three studies had health as a main focus. There were few data on the race-, ethnicity- or cultural-sensitivity of treatment orders. No studies explored the views and experiences of family members/significant others. Most data were from the perspective of justice-involved adults with less from judicial and treatment provider staff. None of the included studies focused on mandated alcohol treatment. All studies were conducted in high-income countries, most in the USA. Findings relate mainly to drug courts in the USA and UK. Studies in the UK are now quite old (2002–2012), and some findings may be outdated e.g., treatment orders might have evolved. Further research is needed on these topics and populations in a wider range of settings. More complete reporting of the specific interventions, equity data, methods, and ethics in primary qualitative studies is needed.

Four (out of 13) of our key findings were moderate confidence and two were low confidence (none were very low confidence), as assessed using GRADE-CERQual. The low confidence findings focused on the negative impacts of treatment orders on justice-involved adults’ mental and physical health. Moderate confidence findings focused on the positive impacts of treatment orders on mental health, justice-involved adults’ views of use of legal sanctions during a treatment order, the perceived impact of legal coercion substance use, and the impact of positive relationship with treatment staff on engagement and compliance with treatment orders. Further evidence could increase our confidence in these findings.

Our synthesis adopted a rigorous, systematic approach but we might have missed relevant studies because we did not conduct extensive supplementary searches. However, an exhaustive sample was not required because our intention was to develop understanding of a phenomenon, not make definitive conclusions about intervention effectiveness. We carefully designed a sampling strategy to select the most relevant and informative studies for analysis, including all those with data on perceived impacts on health.

### Implications for policy and practice

Our findings suggest that treatment orders should adopt a more holistic approach which incorporates healthcare, including mental health treatment and trauma-focused support, and support with housing and employment. Abstinence may be an unrealistic or undesirable goal for many justice-involved adults with substance use problems, therefore mandatory treatment should consider harm-reduction approaches with all collaborating organisations following the same underpinning ideology. Individually tailored treatment goals could be incorporated into treatment orders to enhance participants’ motivation. Treatment orders as complex medical-legal interventions require further theoretical development.

In order to support behaviour change in substance use by justice-involved adults, consistency of judicial and treatment staff and developing trusting relationships should be a priority when implementing treatment orders. Treatment orders should have family-focused services and practices e.g., supporting beneficial family relationships. Also, treatment providers should collaboratively plan with the individual how to address negative family or peer influences. High-quality research evidence including robust qualitative studies on the health and well-being impacts of treatment orders, for alcohol as well as drug use, is urgently needed.

### Reflexivity

The core team (EF, BD, PC, CF) kept a reflexive stance (Olmos-Vega et al., 2023) and interrogated how our professional and personal assumptions could influence interpretation of the data and interpretation of our own findings. For further details, see Additional file 14.

## Conclusions

This is the first qualitative evidence synthesis of which we are aware to explore the perceived impacts on health and well-being of non-custodial mandatory treatment for substance use among justice-involved adults, and the perceived barriers and facilitators to treatment order implementation. Treatment orders for illicit drug use require a holistic treatment approach to address a complex problem that incorporates physical and mental healthcare; supports social needs related to housing, employment/ training; and helps support longer-term changes in substance use. Further qualitative research is needed on how treatment orders affect health and well-being in a wider range of contexts including low-to-middle-income countries, focusing on different kinds of mandated interventions, not just drug courts, and exploring the views of family members. Programme theories and theories of change about how treatment orders affect health and well-being as well as offending must be developed and tested. Any unintended negative consequences of treatment orders on the health and well-being of justice involved adults and their families must be explored.

## Supplementary Information

Below is the link to the electronic supplementary material.

Additional file 1. ENTREQ checklist. Description of data: reporting guideline checklist of reported items

Additional file 2. Protocol amendments. Description of data: deviations from the original protocol

Additional file 3. List of abbreviations. Description of data: list of abbreviations and their meanings used throughout the article

Additional file 4. MEDLINE search strategy. Description of data: the terms used to search the MEDLINE database

Additional file 5. Excluded studies. Description of data: Table of the studies which did not meet review inclusion criteria with exclusion reasons

Additional file 6. ACTIVE and GRIPP2 reporting checklists. Description of data: details of patient and public involvement following reporting guidelines

Additional file 7. Coding framework. Description of data: the coding framework applied in NVivo software and the code definitions

Additional file 8. Table of eligible qualitative studies. Description of data: characteristics of each eligible study in a table

Additional file 9. Table of included studies. Description of data: detailed characteristics of each included study in a table

Additional file 10. Methodological limitations for methodological domains assessed using Critical Appraisal Skills Programme (CASP). Description of data: a table showing the judgements for each CASP methodological domain for each included study

Additional file 11. Studies contributing to categories. Description of data: details of studies, and their key characteristics, that contributed to each of the overarching categories under which synthesis findings are organised

Additional file 12. Summary of Qualitative Findings Table. Description of data: GRADE-CERQual Assessment of confidence summaries of the review findings, the overall CERQual assessments, and an explanation of each CERQual assessment

Additional file 13. Evidence Profile Table. Description of data: GRADE-CERQual Assessment of confidence summaries of the review findings, with judgments for each CERQual component and the overall assessment and its explanation

Additional file 14. Reflexivity. Description of data: details of the authors’ backgrounds, expertise and assumptions relevant to the review

Additional file 15. CRediT author Statement. Description of data: table describing author contributions in the form of the CRediT statement

## Data Availability

No datasets were generated or analysed during the current study.

## References

[CR1] Ahlin, E. M., & Douds, A. S. (2020). *Chapter 15 the problem with problem-solving courts*. The Black Box Remains.

[CR3] Ames, H., Glenton, C., & Lewin, S. (2019). Purposive sampling in A qualitative evidence synthesis: A worked example from A synthesis on parental perceptions of vaccination communication. *Bmc Medical Research Methodology*, *19*(1), 26. 10.1186/s12874-019-0665-4 30704402 10.1186/s12874-019-0665-4 PMC6357413

[CR4] Bates, T. J. (2009). *Drug court: Breaking the black magic spell of drug addiction for women: A qualitative study.* (Doctor of Philosophy). The University of Utah, Dissertation Abstracts International Section A: Humanities and Social Sciences.

[CR5] Baughman, M., Tossone, K., Singer, M. I., & Flannery, D. J. (2019). Evaluation of treatment and other factors that lead to drug court success, substance use reduction, and mental health symptomatology reduction over time. *International Journal of Offender Therapy and Comparative Criminology*, *63*(2), 257. 10.1177/0306624X18789832 30058416 10.1177/0306624X18789832

[CR6] Benoot, C., Hannes, K., & Bilsen, J. (2016). The use of purposeful sampling in a qualitative evidence synthesis: A worked example on sexual adjustment to a cancer trajectory. *Bmc Medical Research Methodology*, *16*(1), 21. 10.1186/s12874-016-0114-6 26891718 10.1186/s12874-016-0114-6 PMC4757966

[CR7] Bevli, S. (2018). *Effectiveness of the substance abuse and crime prevention act: the experiences of Hispanic residents.* (Doctor of Psychology). University of the Rockies, Dissertation Abstracts International: Section B: The Sciences and Engineering.

[CR8] Black, N., Chung, S., Tisdale, C., Fialho, L. S., Aramrattana, A., Assanangkornchai, S., & Brown, A. (2024). An international, multidisciplinary consensus set of patient-centered outcome measures for substance-related and addictive disorders. *Journal of Clinical Medicine*, *13*(7), 2154. 38610919 10.3390/jcm13072154 PMC11012938

[CR9] Bright, D. A., & Martire, K. A. (2013). Does coerced treatment of substance-using offenders lead to improvements in substance use and recidivism? A review of the treatment efficacy literature. *Australian Psychologist*, *48*(1), 69–81.

[CR10] Bronfenbrenner, U. (1989). Ecological Systems Theory: In: Vasta. *Six theories of child development: revised formulations and current issues*, 187–249.

[CR11] Brunton, G., Booth, A., & Carroll, C. (2023). Chapter 9. Framework Synthesis. Draft version (August 2023) version 1. In J. Noyes,. & A. Harden,. (Eds.), *Cochrane-Campbell Handbook for Qualitative Evidence Synthesis*.

[CR120] Campbell, P., Cowie, J., Davis, B., Fenton, C., Todhunter-Brown, A., Bissoso Hernandez, H., Hoyle, L., Carver, H., Connell, C., Dumbrell, J., Hill, R., Davies, N. & France, E. (2025). Effectiveness of legally mandated non-custodial drug and alcohol treatment orders for improved health, well-being, global functioning and quality of life: A systematic review and meta-analysis, *Health and Justice*, 7 Jul 2025 10.1186/s40352-025-00354-4 PMC12958499 41591589

[CR12] CASP (2018). Qualitative studies checklist. Retrieved from https://casp-uk.net/images/checklist/documents/CASP-Qualitative-Studies-Checklist/CASP-Qualitative-Checklist-2018_fillable_form.pdf

[CR13] Cochrane Methods Equity (2021). Progress-Plus framework. Retrieved from https://methods.cochrane.org/equity/projects/evidence-equity/progress-plus

[CR14] Colvin, C. J., Garside, R., Wainwright, M., Munthe-Kaas, H., Glenton, C., Bohren, M. A., & Lewin, S. (2018). Applying GRADE-CERQual to qualitative evidence synthesis findings-paper 4: How to assess coherence. *Implement Sci*, *13*(Suppl 1), 13. 10.1186/s13012-017-0691-8 29384081 10.1186/s13012-017-0691-8 PMC5791039

[CR15] Datchi, C. C., & Ancis, J. R. (2017). Women and adult drug treatment courts: Surveillance, social conformity, and the exercise of agency. In J. R. Ancis (Ed.), *Gender, psychology, and justice: The mental health of women and girls in the legal system* (pp. 101–126). New York University Press; US.

[CR16] DeMatteo, D., Filone, S., & LaDuke, C. (2011). Methodological, ethical, and legal considerations in drug court research. *Behavioral Sciences & the Law*, *29*(6), 806–820. 21971950 10.1002/bsl.1011

[CR17] Deschenes, E. P., Turner, S., & Greenwood, P. W. (1995). Drug court or probation? An experimental evaluation of Maricopa county’s drug court. *Justice System Journal*, *18*(1), 55–73.

[CR18] Desland, M. L., & Batey, R. G. (1992). A 12-month prospective comparison of court-diverted with self-referred heroin users. *Drug and Alcohol Review*, *11*(2), 121–129. 10.1080/09595239200185591 16840266 10.1080/09595239200185591

[CR19] Dickson-Gomez, J., Spector, A., Krechel, S., Li, J., Montaque, H. D. G., Ohlrich, J., & Weeks, M. (2022). Barriers to drug treatment in Police diversion programs and drug courts: A qualitative analysis. *American Journal of Orthopsychiatry*, *92*(6), 692–701. 10.1037/ort0000643Epub 2022 Oct 13. 36227322 10.1037/ort0000643 PMC9993933

[CR20] Eaton, G., & Mews, A. (2019). The impact of short custodial sentences, community orders and suspended sentence orders on reoffending. Retrieved from https://assets.publishing.service.gov.uk/media/5d1c732ee5274a08cdbe45c4/impact-short-custodial-sentences.pdf

[CR21] Eley, S., Malloch, M., McIvor, G., Yates, R., & Brown, A. (2002). *The Glasgow drug court in action: the first six months*. Retrieved from Scotland.

[CR22] Engstrom, R. (2023). *An analysis of binder County DWI court: A case study*. University of St. Thomas, Minnesota.

[CR23] Farhoudian, A., Razaghi, E., Hooshyari, Z., Noroozi, A., Pilevari, A., Mokri, A., & Malekinejad, M. (2022). Barriers And facilitators to substance use disorder treatment: An overview of systematic reviews. *Substance Abuse: Research and Treatment*, *16*, 11782218221118462. 36062252 10.1177/11782218221118462 PMC9434658

[CR24] Festinger, D. S., Dugosh, K. L., Kurth, A. E., & Metzger, D. S. (2016). Examining the efficacy of a computer facilitated HIV prevention tool in drug court. *Drug and Alcohol Dependence*, *162*, 44–50. 26971228 10.1016/j.drugalcdep.2016.02.026 PMC5824990

[CR25] Fischer, M., Geiger, B., & Hughes, M. E. (2007). Female recidivists speak about their experience in drug court while engaging in appreciative inquiry. *International Journal of Offender Therapy & Comparative Criminology*, *51*(6), 703–722. 10.1177/0306624X07299304Epub 2007 Jul 5. 17615439 10.1177/0306624X07299304

[CR26] France, E., Uny, I., Turley, R., Thomson, K., Noyes, J., Jordan, A., & Silveira Bianchim, M. (2023). A meta-ethnography of how children and young people with chronic non‐cancer pain and their families experience and understand their condition, pain services, and treatments. *Cochrane Database of Systematic Reviews*, (10). 10.1002/14651858.CD014873.pub2 10.1002/14651858.CD014873.pub2 PMC10552070 37795766

[CR27] Francis, T. R., & Abel, E. M. (2014). Redefining success: A qualitative investigation of therapeutic outcomes for noncompleting drug court clients. *Journal of Social Service Research*, *40*(3), 325–338. 10.1080/01488376.2013.875094

[CR28] Freeman, K. (2001). *Crime and justice bulletin. New South Wales drug court evaluation: Interim report on health and well-being of participants* (Vol. 53). NSW Bureau of Crime Statistics and Research.

[CR29] Fulkerson, A., Keena, L. D., & O’Brien, E. (2012). Understanding success and nonsuccess in the drug court. *International Journal of Offender Therapy & Comparative Criminology*, *57*(10), 1297–1316. 10.1177/0306624X12447774. Epub 2012 May 28. 22641858 10.1177/0306624X12447774

[CR30] Gallagher, J. R. (2014). Predicting criminal recidivism following drug court: Implications for drug court practice and policy advocacy. *Journal of Addictions & Offender Counseling*, *35*(1), 15–29. 10.1002/j.2161-1874.2014.00021.x

[CR33] Gallagher, J. R., & Wahler, E. A. (2018). Racial disparities in drug court graduation rates: The role of recovery support groups and environments. *Journal of Social Work Practice in the Addictions*, *18*, 113–127.

[CR32] Gallagher, J. R., Nordberg, A., & Lefebvre, E. (2017). Improving graduation rates in drug court: A qualitative study of participants’ lived experiences. *Criminology & Criminal Justice: an International Journal*, *17*(4), 468–484. 10.1177/1748895816682578

[CR31] Gallagher, J. R., Nordberg, A., & Dibley, A. R. (2019a). Improving graduation rates for African Americans in drug court: Importance of human relationships and barriers to gaining and sustaining employment. *Journal of Ethnicity in Substance Abuse*, *18*(3), 387–401. 10.1080/15332640.2017.1381661. Epub 2017 Nov 16. 29144881 10.1080/15332640.2017.1381661

[CR34] Gallagher, J. R., Wahler, E. A., Minasian, R. M., & Edwards, A. (2019b). Treating opioid use disorders in drug court: Participants’ views on using medication-assisted treatments (MATs) to support recovery. *International Criminal Justice Review*, *29*(3), 249–261. 10.1177/1057567719846227

[CR35] Garcia, R. A., Kenyon, K. H., Brolan, C. E., Coughlin, J., & Guedes, D. D. (2019). Court as A health intervention to advance canada’s achievement of the sustainable development goals: A multi-pronged analysis of vancouver’s downtown community court. *Global Health*, *15*(1), 80. 10.1186/s12992-019-0511-9 31847875 10.1186/s12992-019-0511-9 PMC6918572

[CR36] Glenton, C., Carlsen, B., Lewin, S., Munthe-Kaas, H., Colvin, C. J., Tuncalp, O., & Wainwright, M. (2018). Applying GRADE-CERQual to qualitative evidence synthesis findings-paper 5: How to assess adequacy of data. *Implement Sci*, *13*(Suppl 1), 14. 10.1186/s13012-017-0692-7 29384077 10.1186/s13012-017-0692-7 PMC5791045

[CR37] Goldkamp, J. S. (2003). The impact of drug courts. *Criminology & Public Policy*, *2*(2), 197–206.

[CR38] Gottfredson, D. C., & Exum, M. L. (2002). The Baltimore City drug treatment court: One year results from a randomized study. *Journal of Research in Crime and Delinquency*, *39*(3), 337–356.

[CR39] Green, M., & Rempel, M. (2012). Beyond crime and drug use: Do adult drug courts produce other psychosocial benefits. *Journal of Drug Issues*, *42*(2), 156–177. 10.1177/0022042612446592

[CR40] Hall, W., & Lucke, J. (2010a). Crime and Justice Bulletin. Legally coerced treatment for drug using offenders: ethical and policy issues.

[CR41] Hall, W., & Lucke, J. (2010b). *Legally coerced treatment for drug using offenders*. ethical and policy issues.

[CR42] Hamilton, L. (2019). *Health-related quality of life among community-based offenders: How ‘well-being’ affects substance abuse treatment engagement.* (Doctor of Philosophy). Temple University, Dissertation Abstracts International Section A: Humanities and Social Sciences.

[CR43] Harden, A., Thomas, J., Cargo, M., Harris, J., Pantoja, T., & Flemming, K. (2018). Cochrane qualitative and implementation Methods group guidance series—paper 5: Methods for integrating qualitative and implementation evidence within intervention effectiveness reviews. *Journal of Clinical Epidemiology*, *97*, 70–78. 29242095 10.1016/j.jclinepi.2017.11.029

[CR44] Harrell, A., Cavanagh, S., & Roman, J. (1998). *Findings from the evaluation of the D.C. Superior Court drug intervention program*. Retrieved from US.

[CR45] Harrell, A., Roman, J., & Sack, E. (2001). *Drug court services for female offenders, 1996–1999: Evaluation of the Brooklyn Treatment Court*. Retrieved from Washington, DC.

[CR46] Jones, C. G. A. (2013). Early-phase outcomes from a randomized trial of intensive judicial supervision in an Australian drug court. *Criminal Justice and Behavior*, *40*(4), 453–468.

[CR47] Kennedy-Hendricks, A., Bandara, S., Merritt, S., Barry, C. L., & Saloner, B. (2021). Structural and organizational factors shaping access to medication treatment for opioid use disorder in community supervision. *Drug and Alcohol Dependence*, *226*, 108881. 10.1016/j.drugalcdep.2021.108881. Epub 2021 Jun 26. 34218008 10.1016/j.drugalcdep.2021.108881

[CR48] Kerr, J., Tompkins, C., Tomaszewski, W., Dickens, S., Grimshaw, R., Wright, N., & Barnard, M. (2011). *The dedicated drug courts pilot evaluation process study*. Retrieved from Ministry of Justice, UK: www.justice.gov.uk/publications/research.htm

[CR49] Klein, A. (2020). Harm reduction works: Evidence and inclusion in drug policy and advocacy. *Health Care Analysis*, *28*(4), 404–414. 33079317 10.1007/s10728-020-00406-w

[CR50] Kouimtsidis, C., Reynolds, M., & Asamoah, V. (2007). Treatment or prison: Service user and staff experiences of drug treatment and testing orders. *Psychiatric Bulletin*, *31*(12), 463–466. 10.1192/pb.bp.107.014548

[CR51] Lewin, S., Bohren, M., Rashidian, A., Munthe-Kaas, H., Glenton, C., Colvin, C. J., & Carlsen, B. (2018a). Applying GRADE-CERQual to qualitative evidence synthesis findings-paper 2: How to make an overall cerqual assessment of confidence and create a summary of qualitative findings table. *Implement Sci*, *13*(Suppl 1), 10. 10.1186/s13012-017-0689-2 29384082 10.1186/s13012-017-0689-2 PMC5791047

[CR52] Lewin, S., Booth, A., Glenton, C., Munthe-Kaas, H., Rashidian, A., Wainwright, M., & Noyes, J. (2018b). Applying GRADE-CERQual to qualitative evidence synthesis findings: Introduction to the series. *Implement Sci*, *13*(Suppl 1), 2. 10.1186/s13012-017-0688-3 29384079 10.1186/s13012-017-0688-3 PMC5791040

[CR53] Lindenfeld, Z., Kim, S., & Chang, J. E. (2022). Assessing the effectiveness of problem-solving courts on the reduction of overdose deaths in the united states: A difference-in-difference study. *Drug Alcohol Depend Rep*, 2772–7246. 10.1016/j.dadr.2022.100088 10.1016/j.dadr.2022.100088 PMC9948897 36846580

[CR54] MacDonald, J. M., Morral, A. R., Raymond, B., & Eibner, C. (2007). The efficacy of the Rio Hondo DUI court: A 2-year field experiment. *Evaluation Review*, *31*(1), 4–23. 10.1177/0193841X06287189 17259573 10.1177/0193841X06287189

[CR55] Maddox, M. E. (2023). *The effectiveness of drug treatment court: Participants’ recommendations for improvement of the drug treatment court diversion program.* (Doctor in Psychology). William James College, Dissertation Abstracts International: Section B: The Sciences and Engineering.

[CR56] Manuel, J. I., Yuan, Y., Herman, D. B., Svikis, D. S., Nichols, O., Palmer, E., & Deren, S. (2017). Barriers and facilitators to successful transition from long-term residential substance abuse treatment. *Journal of Substance Abuse Treatment*, *74*, 16–22. 28132695 10.1016/j.jsat.2016.12.001 PMC5310811

[CR57] McGuinness, L. A., & Higgins, J. P. T. (2021). Risk-of-bias visualization (robvis): An R package and Shiny web app for visualizing risk-of-bias assessments. *Res Synth Methods*, *12*(1), 55–61. 10.1002/jrsm.1411 32336025 10.1002/jrsm.1411

[CR58] McIvor, G. (2009). Therapeutic jurisprudence and procedural justice in Scottish drug courts. *Criminology & Criminal Justice*, *9*(1), 29–49. 10.1177/1748895808099179

[CR59] McIvor, G., Barnsdale, L., Eley, S., Malloch, M., Yates, R., & Brown, A. (2006). *The operation and effectiveness of the Scottish drug court pilots*. Retrieved from Scotland.

[CR60] McKenna, N. C., & Holtfreter, K. (2021). Trauma-informed courts: A review and integration of justice perspectives and gender responsiveness. *Journal of Aggression Maltreatment & Trauma*, *30*(4), 450–470.

[CR61] Miller, W. R. (2006). Motivational factors in addictive behaviors. In W. R. Miller, & K. M. Carroll (Eds.), *Rethinking substance abuse: What the science shows, and what we should do about it* (pp. 134–150). The Guilford Press.

[CR62] Moore, K. A., Barongi, M. M., & Rigg, K. K. (2017). The experiences of young adult offenders who completed a drug court treatment program. *Qualitative Health Research*, *27*(5), 750–758. 10.1177/1049732316645782. Epub 2016 Jul 10. 27117958 10.1177/1049732316645782

[CR63] Morse, D. S., Cerulli, C., Bedell, P., Wilson, J. L., Thomas, K., Mittal, M., & Chin, N. (2014). Meeting health and psychological needs of women in drug treatment court. *Journal of Substance Abuse Treatment*, *46*(2), 150–157. Epub 2013 Sep 24. 24074850 10.1016/j.jsat.2013.08.017 PMC3860881

[CR64] Morse, D. S., Silverstein, J., Thomas, K., Bedel, P., & Cerulli, C. (2015). Finding the loopholes: A cross-sectional qualitative study of systemic barriers to treatment access for women drug court participants. *Health & Justice*, *3*, 12. 10.1186/s40352-015-0026-2Epub 2015 Jun 17. 26478853 10.1186/s40352-015-0026-2 PMC4607061

[CR65] Munabi-Babigumira, S., Glenton, C., Lewin, S., Fretheim, A., & Nabudere, H. (2017). Factors that influence the provision of intrapartum and postnatal care by skilled birth attendants in low‐and middle‐income countries: A qualitative evidence synthesis. *Cochrane Database of Systematic Reviews*(11). 10.1002/14651858.CD011558.pub2 PMC5721625 29148566

[CR66] Munthe-Kaas, H., Bohren, M. A., Glenton, C., Lewin, S., Noyes, J., Tuncalp, O., & Carlsen, B. (2018). Applying GRADE-CERQual to qualitative evidence synthesis findings-paper 3: How to assess methodological limitations. *Implement Sci*, *13*(Suppl 1), 9. 10.1186/s13012-017-0690-9 29384078 10.1186/s13012-017-0690-9 PMC5791044

[CR67] Murphy, J. (2011). Drug court as both a legal and medical authority. *Deviant Behavior*, *32*(3), 257–291.

[CR68] Narag, R. E., Maxwell, S. R., & Lee, B. (2013). A phenomenological approach to assessing a DUI/DWI program. *International Journal of Offender Therapy & Comparative Criminology*, *57*(2), 229–250. 10.1177/0306624X11431685 22297773 10.1177/0306624X11431685

[CR69] National Guideline Alliance UK. (2017). *Mental health of adults in contact with the criminal justice system: Identification and management of mental health problems and integration of care for adults in contact with the criminal justice system*. In. National Institute for Health and Care Excellence (NICE). 28350429

[CR73] Noyes, J., Hendry, M., Booth, A., Chandler, J., Lewin, S., Glenton, C., & Garside, R. (2016). Current use was established and Cochrane guidance on selection of social theories for systematic reviews of complex interventions was developed. *Journal of Clinical Epidemiology*, *75*, 78–92. 10.1016/j.jclinepi.2015.12.009Epub 2016 Jan 6. 26772607 10.1016/j.jclinepi.2015.12.009

[CR70] Noyes, J., Booth, A., Flemming, K., Garside, R., Harden, A., Lewin, S., & Thomas, J. (2018a). Cochrane qualitative and implementation Methods group guidance series-paper 3: Methods for assessing methodological limitations, data extraction and synthesis, and confidence in synthesized qualitative findings. *Journal of Clinical Epidemiology*, *97*, 49–58. 10.1016/j.jclinepi.2017.06.020Epub 2017 Dec 13. 29247700 10.1016/j.jclinepi.2017.06.020

[CR71] Noyes, J., Booth, A., Lewin, S., Carlsen, B., Glenton, C., Colvin, C. J., & Munthe-Kaas, H. (2018b). Applying GRADE-CERQual to qualitative evidence synthesis findings-paper 6: How to assess relevance of the data. *Implement Sci*, *13*(Suppl 1), 4. 10.1186/s13012-017-0693-6 29384080 10.1186/s13012-017-0693-6 PMC5791042

[CR72] Noyes, J., Booth, A., Moore, G., Flemming, K., Tunçalp, Ö., & Shakibazadeh, E. (2019). Synthesising quantitative and qualitative evidence to inform guidelines on complex interventions: Clarifying the purposes, designs and outlining some methods. *BMJ Global Health*, *4*, e000893. 10.1136/bmjgh-2018-000893 PMC6350750 30775016

[CR74] O’Shaughnessy, B. R., Mayock, P., & Kakar, A. (2024). The recovery experiences of homeless service users with substance use disorder: A systematic review and qualitative meta-synthesis. *International Journal of Drug Policy*, *130*, 104528. 39053034 10.1016/j.drugpo.2024.104528

[CR75] Office of National Statistics (2023). Drug-related deaths and suicide in offenders in the community, England and Wales: 2011 to 2021. Retrieved from https://www.ons.gov.uk/peoplepopulationandcommunity/birthsdeathsandmarriages/deaths/bulletins/drugrelateddeathsandsuicideinoffendersinthecommunityenglandandwales/2011to2021

[CR76] Official Statistics (2023). *Alcohol and drug treatment in secure settings 2021 to 2022: report*. Retrieved from https://www.gov.uk/government/statistics/substance-misuse-treatment-in-secure-settings-2021-to-2022/alcohol-and-drug-treatment-in-secure-settings-2021-to-2022-report

[CR77] Olmos-Vega, F. M., Stalmeijer, R. E., Varpio, L., & Kahlke, R. (2023). A practical guide to reflexivity in qualitative research: AMEE guide 149. *Medical Teacher*, *45*(3), 241–251. 10.1080/0142159X.2022.2057287 35389310

[CR78] Osborne, B., Larance, B., Ivers, R., Deane, F. P., Robinson, L. D., & Kelly, P. J. (2022). Systematic review of guidelines for managing physical health during treatment for substance use disorders: Implications for the alcohol and other drug workforce. *Drug and Alcohol Review*, *41*(6), 1367–1390. 35765725 10.1111/dar.13504 PMC9539873

[CR79] Osilla, K. C., Kulesza, M., & Miranda, J. (2017). Bringing alcohol treatment to driving under the influence programs: Perceptions from first-time offenders. *Alcoholism Treatment Quarterly*, *35*(2), 113–129. 10.1080/07347324.2017.1288484 28943712 10.1080/07347324.2017.1288484 PMC5606326

[CR80] Page, M. J., McKenzie, J. E., Bossuyt, P. M., Boutron, I., Hoffmann, T. C., Mulrow, C. D., & Moher, D. (2021). The PRISMA 2020 statement: An updated guideline for reporting systematic reviews. *Bmj*, *372*, n71. 10.1136/bmj.n71 33782057 10.1136/bmj.n71 PMC8005924

[CR81] Paquette, C. E., Daughters, S. B., & Witkiewitz, K. (2022). Expanding the continuum of substance use disorder treatment: Nonabstinence approaches. *Clinical Psychology Review*, *91*, 102110. 34864497 10.1016/j.cpr.2021.102110 PMC8815796

[CR82] Perkins, A., Livingston, W., Cairns, B., Dumbrell, J., Gardiner, K., Little, S., & Madoc-Jones, I. (2022). Understanding Substance Use and the Wider Support Needs of Scotland’s Prison Population. Retrieved from https://www.gov.scot/binaries/content/documents/govscot/publications/research-and-analysis/2022/09/understanding-substance-use-wider-support-needs-scotlands-prison-population/documents/understanding-substance-use-wider-support-needs-scotlands-prison-population/understanding-substance-use-wider-support-needs-scotlands-prison-population/govscot%3Adocument/understanding-substance-use-wider-support-needs-scotlands-prison-population.pdf

[CR83] Perry, A. E., Darwin, Z., Godfrey, C., McDougall, C., Lunn, J., Glanville, J., & Coulton, S. (2009). The effectiveness of interventions for drug-using offenders in the courts, secure establishments and the community: A systematic review. *Substance Use and Misuse*, *44*(3), 374–400. 10.1080/10826080802347560 19212928 10.1080/10826080802347560

[CR84] Phelps, M. S., Osman, I. H., Robertson, C. E., & Shlafer, R. J. (2022). Beyond pains and gains: Untangling the health consequences of probation. *Health & Justice*, *10*(1), 29. 36181641 10.1186/s40352-022-00193-7 PMC9525231

[CR85] Pollock, A., Campbell, P., Struthers, C., Synnot, A., Nunn, J., Hill, S., & Morley, R. (2019). Development of the ACTIVE framework to describe stakeholder involvement in systematic reviews. *Journal of Health Services Research & Policy*, *24*(4), 245–255. 30997859 10.1177/1355819619841647

[CR86] Powell, C. L. (2012). *Coerced drug treatment in England and wales: An evaluation of drug treatment and testing orders in one locality*. PhD Psychology). University of Leicester.

[CR87] Ricketts, T., Bliss, P., Murphy, K., & Brooker, C. (2005). Engagement with drug treatment and testing orders: A qualitative study. *Addiction Research & Theory*, *13*(1), 65–78. 10.1080/16066350512331328168

[CR88] Ritchie, J., & Spencer, L. (1994). Qualitative data analysis for applied policy research. In B. R. G. Bryman A (Ed.), *Analyzing qualitative data* (pp. 173–194). Routledge.

[CR89] Robertson, A. G. (2016). *NCT02978417. Feasibility study of extended-release Naltrexone (Vivitrol) in drug court settings*. Retrieved from https://clinicaltrials.gov/study/NCT02978417

[CR90] Rodriguez-Monguio, R., Montgomery, B., Drawbridge, D., Packer, I., & Vincent, G. M. (2021). Substance use treatment services utilization and outcomes among probationers in drug courts compared to a matched cohort of probationers in traditional courts. *The American Journal on Addictions / American Academy of Psychiatrists in Alcoholism and Addictions*, *30*, 505–513. 10.1111/ajad.13208 34414632

[CR91] Salzman, H. J. (2023). *Motherhood and substance use: An examination of societal pressures in the motivation to complete court-ordered drug treatment and to desist from future criminal activity and drug use.* (Doctor of Philosophy). University of Manchester, Dissertation Abstracts International: Section B: The Sciences and Engineering, UK.

[CR93] SAMHSA (2023). Results from the 2021 National Survey on Drug Use and Health: Volume 1. Summary of national findings. Retrieved from https://www.samhsa.gov/data/release/2021-national-survey-drug-use-and-health-nsduh-releases

[CR92] SAMHSA (2021). National Survey of Drug Use and Health (NSDUH) Releases. Retrieved from www.samhsa.gov.

[CR94] Sarmiento, E., Seear, K., & Fraser, S. (2019). Enacting alcohol and other drug (Testing)-related harms in an Australian drug court. *Contemporary Drug Problems*, *46*(3), 282–230.

[CR95] Savic, M., Best, D., Manning, V., & Lubman, D. I. (2017). Strategies to facilitate integrated care for people with alcohol and other drug problems: A systematic review. *Substance Abuse Treatment Prevention and Policy*, *12*, 1–12. 28388954 10.1186/s13011-017-0104-7 PMC5384147

[CR96] Schiff, R., & Waegemakers Schiff, J. (2010). Housing needs and preferences of relatively homeless aboriginal women with addiction. *Social Development Issues*, *32*(3), 65–76.

[CR97] Scottish Government (2023). Review of community sentencing options for people with substance use problems: A summary of key findings. Retrieved from https://www.gov.scot/binaries/content/documents/govscot/publications/research-and-analysis/2023/08/review-community-sentencing-options-people-substance-use-problems-summary-key-findings/documents/review-community-sentencing-options-people-substance-use-problems-summary-key-findings/review-community-sentencing-options-people-substance-use-problems-summary-key-findings/govscot%3Adocument/review-community-sentencing-options-people-substance-use-problems-summary-key-findings.pdf

[CR98] Setiawan, A., Sahar, J., Santoso, B., Mansyur, M., & Syamsir, S. B. (2024). Coping mechanisms utilized by individuals with drug addiction in overcoming challenges during the recovery process: A qualitative Meta-synthesis. *Journal of Preventive Medicine and Public Health*, *57*(3), 197. 38726579 10.3961/jpmph.24.042 PMC11164602

[CR99] Staniszewska, S., Brett, J., Simera, I., Seers, K., Mockford, C., Goodlad, S., & Denegri, S. (2017). GRIPP2 reporting checklists: Tools to improve reporting of patient and public involvement in research. *BMJ*, *358*. 10.1136/bmj.j3453 PMC5539518 28768629

[CR101] The SURE Collaboration (2011). SURE Guides for Preparing and Using Evidence-Based Policy Briefs: 5. Identifying and addressing barriers to implementing policy options. Version 2.1 [updated November 2011]. In. Available from https://evidence-impact.org/tools/implementation-considerations/SURE-Guide-5-identifying-and-addressing-barriers-to-implementing-policy-options

[CR100] The Epistemonikos Foundation, Consulting, M. W., & The Norwegian Institute of Public Health for the GRADE-CERQual Project Group. (2022). *GRADE-CERQual interactive summary of qualitative findings (iSoQ) [Computer program]*. Norwegian Institute of Public Health. Available at isoq.epistemonikos.org. Version 1.0 accessed [21/08/24].

[CR102] Tong, A., Flemming, K., McInnes, E., Oliver, S., & Craig, J. (2012). Enhancing transparency in reporting the synthesis of qualitative research: ENTREQ. *Bmc Medical Research Methodology*, *12*(1), 181. 10.1186/1471-2288-12-181 23185978 10.1186/1471-2288-12-181 PMC3552766

[CR103] Trebilcock, J. (2011). No winners: the reality of short term prison sentences. Retrieved from https://howardleague.org/wp-content/uploads/2016/03/No-Winners.pdf

[CR104] Trood, M. D., Spivak, B. L., & Ogloff, J. R. P. (2021). A systematic review and meta-analysis of the effects of judicial supervision on recidivism and well-being factors of criminal offenders. *Journal of Criminal Justice*, *74*, 1. 10.1016/j.jcrimjus.2021.101796

[CR105] Tsuda-McCaie, F., & Kotera, Y. (2022). A qualitative meta‐synthesis of pregnant women’s experiences of accessing and receiving treatment for opioid use disorder. *Drug and Alcohol Review*, *41*(4), 851–862. 35038366 10.1111/dar.13421

[CR106] United Nations (2022). United Nations: Office on Drugs and Crime. World Drug Report 2022. Retrieved from https://www.unodc.org/unodc/en/data-and-analysis/world-drug-report-2022.html

[CR2] Unopened after Thirty Years In C C. Spohn & P. K. Brennan (Eds.), *Handbook on sentencing policies and practices in the 21st century*. New York: Routledge.

[CR107] Veritas Health Innovation. (2022). *Covidence systematic review software*. Retrieved from Available at www.covidence.org.

[CR108] Vigdal, M. I., Moltu, C., Bjornestad, J., & Selseng, L. B. (2022). Social recovery in substance use disorder: A metasynthesis of qualitative studies. *Drug and Alcohol Review*, *41*(4), 974–987. 35104369 10.1111/dar.13434 PMC9306622

[CR109] Werb, D., Kamarulzaman, A., Meacham, M. C., Rafful, C., Fischer, B., Strathdee, S. A., & Wood, E. (2016). The effectiveness of compulsory drug treatment: A systematic review. *International Journal of Drug Policy*, *28*, 1–9. 26790691 10.1016/j.drugpo.2015.12.005 PMC4752879

[CR110] Wermink, H., Been, J., Schuyt, P., van Wijck, P., & Blokland, A. (2023). The price of retribution: evidence from the willingness to pay for short-term prison sentences compared to community service orders. *Journal of Experimental Criminology*, 1–32.

[CR111] Wexler, D. B., & Winick, B. J. (1992). Therapeutic jurisprudence: A new approach to mental health law. In J. R. P. Ogloff (Ed.), *The law and psychology: The broadening of the discipline* (pp. 211–240). Carolina: Springer.

[CR112] Yardley, L., Ainsworth, B., Arden-Close, E., & Muller, I. (2015). The person-based approach to enhancing the acceptability and feasibility of interventions. *Pilot and Feasibility Studies*, *1*, 1–7. 27965815 10.1186/s40814-015-0033-z PMC5153673

[CR113] Zanis, D. A., Mulvaney, F., Coviello, D., Alterman, A. I., Savitz, B., & Thompson, W. (2003). The effectiveness of early parole to substance abuse treatment facilities on 24-month criminal recidivism. *Journal of Drug Issues*, *33*(1), 223–235.

