# Supplementary material for: Understanding the health and well-being impacts and implementation barriers and facilitators of legally-mandated non-custodial drug and alcohol treatment for justice-involved adults: a qualitative evidence synthesis
Source: Health Justice. 2025 Oct 1;13:58. doi: 10.1186/s40352-025-00361-5 (PMC12487214; doi:10.1186/s40352-025-00361-5)
Supplement: Supplementary file 1 — Additional file 1. ENTREQ checklist. Description of data: reporting guideline checklist of reported items [file 40352_2025_361_MOESM1_ESM.docx]

# Additional file 1. ENTREQ checklist (Enhancing transparency in reporting the synthesis of qualitative research) *

| **No. Item** | **Guide and description** | **Reported on Page #** |
| --- | --- | --- |
| 1. Aim | State the research question the synthesis addresses | Page 7 |
| 2. Synthesis methodology | Identify the synthesis methodology or theoretical framework which underpins the synthesis, and describe the rationale for choice of methodology (e.g. meta- ethnography, thematic synthesis, critical interpretive synthesis, grounded theory synthesis, realist synthesis, meta-aggregation, meta-study, framework synthesis). | Page 8 |
| 3. Approach to searching | Indicate whether the search was pre-planned (comprehensive search strategies to seek all available studies) or iterative (to seek all available concepts until theoretical saturation is achieved). | Page 10 |
| 4. Inclusion criteria | Specify the inclusion/exclusion criteria (e.g. in terms of population, language, year limits, type of publication, study type). | Pages 9 |
| 5. Data sources | Describe the information sources used (e.g. electronic databases, grey literature databases (digital thesis, policy reports), relevant organisational websites, experts, information specialists, generic web searches (Google Scholar), hand searching, reference lists) and when the searches were conducted; provide the rationale for using the data sources. | Page 10 |
| 6. Electronic Search strategy | Describe the literature search (e.g. provide electronic search strategies with population terms, clinical or health topic terms, experiential or social phenomena related terms, filters for qualitative research and search limits). | Additional file 4 |
| 7. Study screening methods | Describe the process of study screening and sifting (e.g. title, abstract and full text review, number of independent reviewers who screened studies) | Pages 10-12 |
| 8. Study  characteristics | Present the characteristics of the included studies (e.g. year of publication, country, population, number of participants, data collection, methodology, analysis,  research questions). | Additional files 8 & 9; also pages 16-19 |
| 9. Study selection results | Identify the number of studies screened and provide reasons for study exclusion (e.g. for comprehensive searching, provide numbers of studies screened and reasons for exclusion indicated in a figure/flowchart; for iterative searching describe reasons for study exclusion and inclusion based on modifications the research question and/or contribution to theory development). | Figure 1 PRISMA flowchart; Additional file 5. |
| 10. Rationale for appraisal | Describe the rationale and approach used to appraise the included studies or selected findings (e.g. assessment of conduct (validity and robustness), assessment of reporting (transparency), assessment of content and utility of the findings). | Page 14-15 |
| 11. Appraisal items | State the tools, frameworks and criteria used to appraise the studies or selected findings (e.g. Existing tools: CASP, QARI, COREQ, Mays and Pope [25]; reviewer developed tools; describe the domains assessed: research team, study design, data analysis and interpretations, reporting). | Page 14 |
| 12. Appraisal process | Indicate whether the appraisal was conducted  independently by more than one reviewer and if consensus was required. | Page 14 |
| 13. Appraisal results | Present results of the quality assessment and indicate  which articles, if any, were weighted/excluded based on the assessment and give the rationale. | Pages 22, Figure 2, Additional file 10 |
| 14. Data extraction | Indicate which sections of the primary studies were analysed and how were the data extracted from the primary studies? (e.g. all text under the headings “results /conclusions” were extracted electronically and entered into a computer software). | Pages 12- 13 |
| 15. Software | State the computer software used, if any. | Pages 10 & 13 |
| 16. Number of reviewers | Identify who was involved in coding and analysis. | Pages 12-14 (anonymised) |
| 17. Coding | Describe the process for coding of data (e.g. line by  line coding to search for concepts). | Pages 12-13 |
| 18. Study  comparison | Describe how were comparisons made within and across studies (e.g. subsequent studies were coded into pre-existing concepts, and new concepts were  created when deemed necessary). | Page 13 |
| 19. Derivation of themes | Explain whether the process of deriving the themes or  constructs was inductive or deductive. | Pages 13 |
| 20. Quotations | Provide quotations from the primary studies to illustrate themes/constructs, and identify whether the quotations were participant quotations or the author’s interpretation | Findings section |
| 21. Synthesis output | Present rich, compelling and useful results that go beyond a summary of the primary studies (e.g. new interpretation, models of evidence, conceptual models, analytical framework, development of a new theory or construct). | Findings & discussion sections |

## Reference: Tong A, Flemming K, McInnes E, Oliver SA, Craig J. Enhancing transparency in reporting the synthesis of qualitative research: ENTREQ. BMC Medical Research Methodology 2012, 12:181.
