# Supplementary material for: Understanding the health and well-being impacts and implementation barriers and facilitators of legally-mandated non-custodial drug and alcohol treatment for justice-involved adults: a qualitative evidence synthesis
Source: Health Justice. 2025 Oct 1;13:58. doi: 10.1186/s40352-025-00361-5 (PMC12487214; doi:10.1186/s40352-025-00361-5)
Supplement: Supplementary file 2 — Additional file 2. Protocol amendments. Description of data: deviations from the original protocol [file 40352_2025_361_MOESM2_ESM.docx]

# Additional file 2. Protocol amendments

In the protocol, the qualitative evidence synthesis aim and review question were worded in reference to the sister quantitative review, therefore, we have re-phrased them for clarity. We searched an additional database the National Criminal Justice Reference Service (NCJRS) identified as important during supplementary searches. We did not conduct planned searches of the reference lists of included and secondary research studies, or forward citation searching using Citation Chaser software for included studies (Haddaway, Grainger, & Gray, 2021) due to the very high volume of eligible qualitative studies from which to sample studies for synthesis and the lack of need for an exhaustive sample. Instead, we searched reference lists of included trials in the quantitative review specifically to identify linked qualitative studies.

We had planned to exclude any study which did not use recognisable methods of data analysis. We made an exception in order to include the only qualitative study linked to a trial in the sister quantitative review (Harrell, Cavanagh, & Roman, 1998). The study authors had not described their qualitative data analysis methods but presented useful findings.

A second reviewer checked all extracted data, rather than just an initial subset. The reason was that accessing data extracted by one other reviewer was not possible in Covidence; some aspects of data extraction for the qualitative evidence synthesis only required one reviewer but other tasks (appraisal of methodological limitations and the quantitative review) required two independent reviewers. This meant that two reviewers must have extracted and approved all types of data before they could be viewed.

## References

Haddaway, N., Grainger, M., & Gray, C. (2021). Citationchaser: An R package and Shiny app for forward and backward citations chasing in academic searching. . Available at: <https://www.eshackathon.org/about.html> [Access date: 21/08/2024].

Harrell, A., Cavanagh, S., & Roman, J. (1998). *Findings from the evaluation of the D.C. Superior Court drug intervention program*. Retrieved from US:
