# Supplementary material for: Understanding the health and well-being impacts and implementation barriers and facilitators of legally-mandated non-custodial drug and alcohol treatment for justice-involved adults: a qualitative evidence synthesis
Source: Health Justice. 2025 Oct 1;13:58. doi: 10.1186/s40352-025-00361-5 (PMC12487214; doi:10.1186/s40352-025-00361-5)
Supplement: Supplementary file 3 — Additional file 3. List of abbreviations. Description of data: list of abbreviations and their meanings used throughout the article [file 40352_2025_361_MOESM3_ESM.docx]

# Additional file 3. List of abbreviations

[anonymised for review]

| **Abbreviation** | **Term** |
| --- | --- |
| AA | Alcoholics Anonymous |
| ACTIVE | Authors and Consumers Together Impacting on eVidencE |
| ASSIA | Applied Social Science Index and Abstracts |
| CASP | Critical Appraisal Skills Programme |
| CINAHL | Cumulative Index to Nursing and Allied Health |
| CERQual | Confidence in the Evidence from Reviews of Qualitative research |
| DUI | Driving under the influence |
| DWI | Driving while impaired |
| ENTREQ | Enhancing transparency in reporting the synthesis of qualitative research |
| GRADE | Grading of Recommendations Assessment, Development, and Evaluation |
| GRIPP2 | Guidance for Reporting Involvement of Patients and the Public Version 2 |
| IBSS | International Bibliography of Social Science |
| ICTRP | International Clinical Trials Registry Platform |
| iSoQ | Interactive Summary of Qualitative Findings |
| MAT | Medication-assisted treatment |
| NA | Narcotics Anonymous |
| NCJRS | National Criminal Justice Reference Service |
| NHS | National Health Service, UK |
| NICE | The National Institute for Health and Care Excellence, UK |
| PPI | Patient and Public Involvement |
| PRISMA | Preferred Reporting Items for Systematic Reviews and Meta-Analyses |
| UK | United Kingdom |
| US/USA | United States of America |
