# Supplementary material for: Understanding the health and well-being impacts and implementation barriers and facilitators of legally-mandated non-custodial drug and alcohol treatment for justice-involved adults: a qualitative evidence synthesis
Source: Health Justice. 2025 Oct 1;13:58. doi: 10.1186/s40352-025-00361-5 (PMC12487214; doi:10.1186/s40352-025-00361-5)
Supplement: Supplementary file 4 — Additional file 4. MEDLINE search strategy. Description of data: the terms used to search the MEDLINE database [file 40352_2025_361_MOESM4_ESM.docx]

Additional file 4. MEDLINE Search Strategy

| Source | Search strategy |
| --- | --- |
| 1. MEDLINE via OVID | 1 exp Substance-Related Disorders/pc, rh  2 (substance adj ("use" or misuse or abuse or problem* or disorder* or addiction* or dependen*)).ti,ab.  3 (Alcohol* adj ("use" or misuse or abuse or problem* or disorder* or addiction* or dependen*)).ti,ab.  4 (Drug* adj ("use" or misuse or abuse or problem* or disorder* or addiction* or dependen*)).ti,ab.  5 (Opioid* adj ("use" or misuse or abuse or problem* or disorder* or addiction* or dependen*)).ti,ab.  6 SUD.ti,ab.  7 "Alcoholic ketoacidosis".ti,ab.  8 or/1-7  9 "12 step programme*".ti,ab.  10 "alcohol treatment*".ti,ab.  11 "cognitive behavior* therapy".ti,ab.  12 "community based intervention*".ti,ab.  13 "community reinforcement".ti,ab.  14 detoxification.ti,ab.  15 "drug counselling".ti,ab.  16 "mandatory test*".ti,ab.  17 "medication assisted treatment*".ti,ab.  18 "non custodial".ti,ab.  19 "problem solving court*".ti,ab.  20 "random test*".ti,ab.  21 "regular test*".ti,ab.  22 "substance use treatment*".ti,ab.  23 "treatment order*".ti,ab.  24 "twelve step program*".ti,ab.  25 "recovery capital".ti,ab.  26 "therapeutic jurisprudence".ti,ab.  27 "Community payback order*".ti,ab.  28 or/9-27  29 exp Criminal Law/  30 exp Law Enforcement/mt  31 exp Driving Under the Influence/pc  32 "criminal justice".ti,ab.  33 Crime/pc  34 crime*.ti,ab.  35 criminal.ti,ab.  36 judicial*.ti,ab.  37 or/29-36  38 8 and 28 and 37  39 "Abstinence tag*".ti,ab.  40 "Addressing substance related offending".ti,ab.  41 "Adult treatment court*".ti,ab.  42 "Alcohol abstinence monitoring requirement*".ti,ab.  43 "Alcohol abstinence tag*".ti,ab.  44 "Alcohol court*".ti,ab.  45 "Alcohol monitoring tag*".ti,ab.  46 "Alcohol rehabilitation requirement*".ti,ab.  47 "Alcohol specified activity requirement".ti,ab.  48 "Alcohol tag*".ti,ab.  49 "Alcohol treatment requirement*".ti,ab.  50 "Driving under the influence court".ti,ab.  51 "Driving while intoxicated court*".ti,ab.  52 "Drug abstinence order*".ti,ab.  53 "Drug abstinence requirement*".ti,ab.  54 "Drug court*".ti,ab.  55 "Drug rehabilitation requirement*".ti,ab.  56 "Drug treatment and testing order*".ti,ab.  57 "Drug treatment court*".ti,ab.  58 "Electronic alcohol tag*".ti,ab.  59 "Family drug and alcohol court*".ti,ab.  60 "Low intensity alcohol program".ti,ab.  61 "mandatory alcohol treatment*".ti,ab.  62 "Sobriety court*".ti,ab.  63 "Sobriety project".ti,ab.  64 AAMR.ti,ab.  65 DTTO.ti,ab.  66 or/39-65  67 38 or 66  68 limit 67 to english language |
