# Supplementary material for: Understanding the health and well-being impacts and implementation barriers and facilitators of legally-mandated non-custodial drug and alcohol treatment for justice-involved adults: a qualitative evidence synthesis
Source: Health Justice. 2025 Oct 1;13:58. doi: 10.1186/s40352-025-00361-5 (PMC12487214; doi:10.1186/s40352-025-00361-5)
Supplement: Supplementary file 6 — Additional file 6. ACTIVE and GRIPP2 reporting checklists. Description of data: details of patient and public involvement following reporting guidelines [file 40352_2025_361_MOESM6_ESM.docx]

# Additional file 6. ACTIVE and GRIPP2 Reporting checklists

## Stakeholder/Patient and Public Involvement in two complementary reviews

This document reports patient and public involvement (PPI) and the stakeholder involvement by reporting:

1. GRIPP2 short form items (Table A)
2. ACTIVE framework descriptions of methods of involvement (Table B)
3. ACTIVE framework summary (Table C)

| **GRIPP2 short form** | | |
| --- | --- | --- |
| **Section and topic** | **Item** | **Report from this project** |
| 1. Aim | Report the aim of PPI in the study | The aim of stakeholder/PPI involvement was to advise relating to aspects of the review including writing the protocol (stakeholders only), search strategy, selection of studies for inclusion, to provide insight into review data extraction and analysis/interpretation of findings, review of final publication draft. |
| 2. Methods | Provide a clear description of the methods used for PPI in the study | We used two key modes of stakeholder/PPI engagement - online meetings and emails with:   1. Stakeholders (n= 5) who had professional knowledge and experience of the topic area. 2. People and family members with lived experience of the topic area (n=5).   NB: Our [anonymised] co-investigator member participated in both groups.  We ensured that enough time was given for people to respond to commenting on documents (e.g. protocol and search strategy).  See ACTIVE framework for detailed description of methods.* |
| 3. Study results | Outcomes—Report the results of PPI in the study, including both positive  and negative outcomes | See ACTIVE framework for detailed description of the outcomes of meetings. |
| 4. Discussion and conclusions | Outcomes—Comment on the extent to which PPI influenced the study overall. Describe positive and negative effects. | The response from stakeholders and PPI members was enthusiastic and extremely helpful throughout the review. Stakeholders’ professional knowledge and expertise was essential in conceptualising the reviews, writing and finalising the protocol, study selection and sampling decisions, analysis/interpretation of findings and dissemination. Our PPI group joined the review at the sampling stage and were helpful in determining the positive and negative aspects of treatment orders and the types of people they viewed as being important to include within the review. |
| 5. Reflections/critical perspective | Comment critically on the study, reflecting on the things that went well  and those that did not, so others can learn from this experience | The topic of substance use problems was potentially sensitive. The combined input of professionals with expertise and knowledge of the topic area and people with lived/family member experiences proved extremely valuable.  Despite concerted efforts we were unable to secure involvement from related charitable organisations and judicial personnel. Expert judicial input would have been very helpful, especially in explaining and understanding some judicial processes. PPI/stakeholder input assisted study sampling decisions, highlighted key findings and gaps in research and gave useful feedback on the research outputs. |
| **Table A: GRIPP2 short-form for stakeholder involvement** | | |

*See changes from protocol (below)

| ACTIVE framework construct | Description |
| --- | --- |
| Who is involved? | **Stakeholder group**: A closed group of five people including: three scientific / academic members involved in substance use research; one PhD student involved in substance use research; one PPI co-investigator.  **PPI Advisory Group:** three family members of people with substance use problems who had been through a treatment order, two people with lived experience of substance use and treatment orders, and one PPI Co-investigator. |
| How were people recruited? | **Stakeholder Group:** they originally conceived the review topic submitted to the [funder name] .  **PPI Advisory Group:** Recruited in March 2023 through the stakeholder group’s networks and contacts. |
| What happened?  When did it happen (stage & level)? | **Stakeholder Group**  Contributed to early-stage discussions involving the scope of the review, writing of the review protocol, qualitative study sampling framework wording/description/coding of the main themes from the review results.  They reviewed and gave written comments on draft versions of the protocol and final publication. The stakeholder group met online with members of the review team in October 2023, February 2024 and February 2025.  **Meeting 1 (October 2023):**  - Agreed group input into the review protocol including search strategy (deadline for submission of protocol 31.10.24).  - Discussed the PPI role of people with lived experience of substance use, and best ways of communicating and working together.  - Discussed contacts to invite potential stakeholders to advise and contribute from judiciary and 3^rd^ sector organisations.  **Meeting 2 (February 2024):**  - Update on review progress  - Discussed potential publications for inclusion  - Potential PPI contacts who are people with lived experience and family members.  - No progress with potential judicial or third sector members.  - Discussed data extraction.  **Meeting 3 (February 2025):**  - Discussed dissemination following completion and publication of the reviews.  **NB: There were no further meetings with the stakeholder group, but contact was maintained by email.**  **PPI Advisory Group meeting 1, April 2024**  The PPI group met online with members of the review team in April 2024, June 2024 and July 2024. The group was given an overview of the review and an update on the eligible studies, and topic areas. They provided their perspectives on the review content including the sampling framework and inclusion of studies.  - **Provided perspectives on positive aspects of Treatment Orders (TO)**   - Regular drug testing and group support within a TO can help - Some conditions of TOs are helpful (e.g. night-time curfew, although drugs can still be accessed during the day) - Being able to return to work is really important - Gaining a sense of responsibility is key to success of TOs - Community Psychiatric Nurse and social care can be helpful   - **Aspects of treatment orders that do not work well:**   - Community Psychiatric Nurse & social care support is often unreliable or unavailable - Poor communication between agencies - Lack of help with mental health problems (sometimes leading to risk of suicide) - General practitioners lack knowledge - Lack of specialised staff - Lack of available, specialised rehabilitation centres - Burden of care is on families and voluntary services - The root of addiction is not addressed - The danger of relapse is always present   The group also discussed **types of people** that they consider important to the review including people with both mental AND addiction problems and people from ethnic minorities.  **PPI Advisory Group meeting 2, June 2024**  The group were given an update on the review and we shared some initial findings.  **Summary of PPI Group feedback**   - The family are still largely ignored/avoided and rely on local community groups set up by family members. More research should be carried out about the needs of the family - Mental health issues are nearly always at the root of substance use problems, this should be further researched - There is a lot of controversy around the use of methadone. This should not be a long-term solution, but still is. - It was agreed that the relationship with the judge/sheriff is important. Glasgow provides some good examples of this but often the treatment order does not take into account the personal background and needs of the individual – tailoring of treatment orders would improve compliance - Possessing specific knowledge of treatment orders and the individual involved is important in gaining respect and trust.   **PPI Meeting 3, July 2024** (mainly dedicated to the quantitative review)  Co-authorship of the journal articles was discussed with the group, and how we could work together. They were considering being involved.  Two members are keen to be involved in co-authorship of the PPI section. One member is not keen, two members were not present.  See Table C for a summary, using descriptors from ACTIVE framework. |

**Table B: ACTIVE framework constructs for stakeholder involvement in two complementary reviews**

| **Table C Summary of PPI / stakeholder involvement in the reviews using the ACTIVE framework** | | | | | | | | | | | | | | |
| --- | --- | --- | --- | --- | --- | --- | --- | --- | --- | --- | --- | --- | --- | --- |
|  |  | **What happened?** | | **Stage and level of involvement**** | | | | | | | | | | |
| **Who was involved?** | **How were they recruited?** | **Approach** | **Method** | **1** | **2** | **3** | **4** | **5** | **6** | **7** | **8** | **9** | **10** | **11** |
| Stakeholder Group | Closed; existing group | Multiple-time closed event approach | Direct interaction | Con | Ctb | Ctb | Ctb |  | Ctb |  |  | (R) | I |  |
| PPI Advisory Group* | Closed; invitation only | Multiple-time closed event approach | Direct interaction |  |  |  |  |  | Ctb |  |  | R | I |  |

Patients*: patients, carers and/or their families; Con: controlling; I: influencing; Ctb: contributing; R: receiving. Blank cells in ‘Stage of involvement’ indicate that there was no stakeholder/PPI involvement.

Stage/level using Cochrane Collaboration 1. development of question; 2. Plan methods; 3. Write & publish protocol; 4. Develop search; 5. Run search; 6. Select studies; 7. Collect data; 8. Assess risk of bias; 9. Analyse data 10. Interpret findings; 11. Write/publish reviews
