# Supplementary material for: Understanding the health and well-being impacts and implementation barriers and facilitators of legally-mandated non-custodial drug and alcohol treatment for justice-involved adults: a qualitative evidence synthesis
Source: Health Justice. 2025 Oct 1;13:58. doi: 10.1186/s40352-025-00361-5 (PMC12487214; doi:10.1186/s40352-025-00361-5)
Supplement: Supplementary file 7 — Additional file 7. Coding framework. Description of data: the coding framework applied in NVivo software and the code definitions [file 40352_2025_361_MOESM7_ESM.docx]

Additional file 7. NVivo coding framework

| Name of code  Subcodes | Description |
| --- | --- |
| **Adverse Treatment Order (TO) consequences** | Any adverse or unintended consequences of a mandated treatment e.g., side effects of medication assisted treatment (MAT), damage to health, loss of job, stigma |
| **Family members** | Family members or significant others of TO recipients  - this is for coding things family members have directly said/done etc AND what other people indirectly report about family members |
| Aftercare or follow-up | Experiences, attitudes perceptions of aftercare or follow up after the TO has ended |
| Attitudes to TO | Attitudes to the TO/ intervention re acceptability, appropriateness, credibility |
| Health, mental health, trauma | Issues to do with Health / mental health /trauma -before during or after the TO |
| Knowledge & skills | Knowledge & skills that might affects success or failure of the TO |
| Motivation to change |  |
| **Legal staff mandating TO** | Legal staff mandating TO - includes any legal staff such as lawyers, judges, courtroom assistants |
| Aftercare or follow-up | Experiences, attitudes perceptions of aftercare or follow up after the TO has ended |
| Attitudes to TO | Attitudes to the TO/ intervention re acceptability, appropriateness, credibility |
| -Attitudes to MAT | legal staff attitudes and view of medication assisted treatment |
| -Attitudes to monitoring & sanctions | legal staff attitudes and view of monitoring & sanctions |
| Knowledge & skills | Knowledge & skills that might affect success or failure of the TO |
| Personal characteristics, qualities | Personal characteristics/qualities of the legal staff e.g. how they treat the recipient of the TO |
| Recipient motivation to change | Legal staff's views of the TO recipients' motivation to change particularly re substance use |
| Recipient relationships with family | What legal staff say about recipients' relationship with their own family |
| Relationships between staff | Relationships between staff - either with other legal staff or intervention/ TO providers |
| Relationships with recipients | Relationships with recipients of mandated treatment |
| **Legal System factors** | Legal System factors or constraints affecting the TO |
| Accessibility | The accessibility or not of the court/ legal premises for the TO |
| Bureaucracy | Administrative burden, bureaucratic procedures - any aspects of the court/ legal organisations which facilitate or impede the success of the TO |
| External communication | External communication by the court/ legal organisations which facilitate or impede the success of the TOs, e.g. comms with the treatment provider, which aid or impede the success of the TO |
| Financial resources | Financial resources of the of the court/ legal organisations which facilitate or impede the success of the TO |
| Human resources | Human resources of the court/ legal organisations which facilitate or impede the success of the TO |
| Ideology | Ideology of the court/ legal organisations that affects or underpins treatment/the TO e.g. abstinence is required, therapeutic jurisprudence, punishment is required |
| Internal communication | Internal communication within f of the court/ legal organisations which facilitate or impede the success of the TO |
| Management and leadership | Management and /or leadership f of the court/ legal organisations which facilitate or impede the success of the TO |
| **Other Implementation issues** | Anything that affects implementation that doesn't fit under other codes |
| **Other stakeholders involved in TO** | Anyone who is not a legal staff member or a provider delivering treatment |
| Aftercare or follow-up | Experiences, attitudes perceptions of aftercare or follow up after the TO has ended |
| Attitudes to TO | Attitudes to the TO/ intervention re acceptability, appropriateness, credibility |
| Knowledge & skills | Knowledge & skills that might affect success or failure of the TO |
| Personal characteristics, qualities | Personal characteristics/qualities of the stakeholders e.g., how they treat the recipient of the TO |
| Recipient motivation to change | other stakeholders' views of the TO recipients' motivation to change particularly re substance use |
| Recipient relationships with family | What other stakeholders say about recipients' relationships with their own family |
| Relationships with recipients | Relationships with recipients of mandated treatment |
| **People delivering TO** | people who are actually delivering the treatment for drug and alcohol - could be health professionals, counsellors, psychologists |
| Aftercare or follow-up | Experiences, attitudes perceptions of aftercare or follow up after the TO has ended |
| Attitudes to TO | Attitudes to the TO/ intervention re acceptability, appropriateness, credibility |
| -Attitudes to MAT | Attitudes of staff delivering TOs of medication assisted treatments such as methadone |
| -Attitudes to monitoring & sanctions | treatment staff attitudes to the treatment order monitoring and sanctions |
| Knowledge & skills | Knowledge & skills that might affect success or failure of the TO |
| Personal characteristics, qualities | Personal characteristics/qualities of the legal staff e.g. how they treat the recipient of the TO |
| Recipient motivation to change | views of the People delivering TOs of the TO recipients' motivation to change particularly re substance use |
| Recipient relationships with family | What people delivering the TO say about recipients’ relationships with their own family |
| Relationships between staff | Data on the relationships between different members of the treatment provider/co-ordinating team |
| Relationships with recipients | Relationships with recipients of mandated treatment |
| **Perceived impact of TO** | Perceived impact of TO on specific issues |
| Impact on employment, education | impact of TO/ intervention on employment e.g. get job, lose job, can't work, training, getting an education |
| Impact on family | e.g. how it affects parenting, children, child custody |
| Impact on finances | impact of TO/ intervention on finances |
| Impact on health | impact of TO/ intervention on health – physical or emotional /mental health |
| Impact on housing, homelessness | impact of TO/ intervention on housing issues or homelessness |
| Impact on substance use | impact of TO/ intervention on drug and or alcohol use either positive or negative |
| **Recipients of TO** | people who have committed a crime who have been legally mandated to participate in treatment order |
| Aftercare or follow-up | Experiences, attitudes perceptions of aftercare or follow up after the TO has ended |
| Attitudes to TO | Attitudes to the TO/ intervention re acceptability, appropriateness, credibility |
| -Attitudes to MAT | Attitudes of recipients specifically to medication assisted treatments |
| -Attitudes to monitoring | Attitudes to the treatment order/drug court monitoring processes of the recipients of the TO |
| -Attitudes to sanctions | Attitudes to treatment order/drug court sanctions if they get caught using substances etc during their TO |
| Experiences of court | how they perceived or experienced the actual experience of attending court |
| Health, mental health, trauma | Issues to do with Health / mental health /trauma -before during or after the TO |
| Knowledge & skills | Knowledge & skills that might affect success or failure of the TO |
| Motivation to change | What motivates the person to want to stop substance use |
| Other treatment - not TO | Data on other treatment or counselling or support such as AA or Narcotics anonymous, outside of their / in addition to treatment order for the people who use substances and commit crime |
| Relationships with family | Relationships with family or significant others affecting success or failure of TO |
| Relationships with friends | findings related to the recipient of the TO's relationship with friends and social networks other than family or staff |
| Relationships with staff | Relationships with staff - either legal staff or providers |
| Resources affecting TO | Resources affecting intervention access (e.g. financial, employment, housing) |
| **TO provider organisation factors** | Aspects, issues, constraints, factors etc of the treatment/ treatment order provider |
| Accessibility | The accessibility or not of the treatment organisation or service providing to treatment for the TO e.g. for reasons of disability, transport issues, for people with children /childcare issues |
| Bureaucracy | Administrative burden, bureaucratic procedures - any aspects of the court/ legal organisations which facilitate or impede the success of the TO |
| External communication | External communication by the health service or treatment provider organisation with other organisations e.g. the court which aid or impede the success of the TO |
| Financial resources | Financial resources of the treatment service or organisation related to delivering or providing the TO |
| Human resources | Human resources of the treatment service or organisation related to delivering or providing the TO |
| Ideology | Ideology of the court/ legal organisations that affects or underpins treatment/the TO e.g. abstinence is required, therapeutic jurisprudence, punishment is required |
| Internal communication | Internal communication within the court/ legal organisations which facilitate or impede the success of the TO |
| Management and leadership | Management and /or leadership within the health service or treatment provider organisation which aid or impede the success of the TO |

**Key: TO= treatment order**
