# Supplementary material for: Understanding the health and well-being impacts and implementation barriers and facilitators of legally-mandated non-custodial drug and alcohol treatment for justice-involved adults: a qualitative evidence synthesis
Source: Health Justice. 2025 Oct 1;13:58. doi: 10.1186/s40352-025-00361-5 (PMC12487214; doi:10.1186/s40352-025-00361-5)
Supplement: Supplementary file 8 — Additional file 8. Table of eligible qualitative studies. Description of data: characteristics of each eligible study in a table [file 40352_2025_361_MOESM8_ESM.docx]

# Additional file 8. Table of eligible qualitative studies

| **Author/**  **year** | **Country /region** | **Aim/s** | **Intervention name/Description of intervention** | **Research**  **Participants** | **Race/ethnicity of justice-involved research participants** | **Type of substance(s) used by justice-involved research participants** | **Research Design** | **Data collection methods** | **Data analysis methods** | **Sampled for inclusion in analysis** |
| --- | --- | --- | --- | --- | --- | --- | --- | --- | --- | --- |
| Abdul-Malik  2022 | USA,  New York | Explore the lived experiences of 12 African Americans treatment plans through drug courts in Duchess County, New York State. | Drug Court:  A mandated judicial supervision and addiction treatment alternative to incarceration for drug offenders, uses a drug treatment plan with approach for sequencing resources and activities and identifies benchmarks of progress to guide evaluation. | Justice-involved-men; Justice-involved women | Black, African, African-Caribbean, or African American | Drugs | Interview study | Semi-structured interviews, interviews | Data not extracted for eligible studies | No |
| Baker  2009 | USA,  2 cities in southwestern region | To take an in-depth look at two drug courts (firstly Oak County Court and then Plains County Court (pseudonyms) to further elaborate our understanding of team decision-making, and specifically how court staff conflict was resolved. | The “Oak County Drug Court” & “Plains County Court (pseudonyms):  Operates as a pre-adjudicative diversionary program. Eligible participants are non-violent felony-level drug offenders who agree to plea guilty to the offense. Their sentences are then suspended until completion of the program. Those participants who successfully complete the drug court program have the charges for the original crime dismissed and cleared from their criminal record. Those participants who fail to complete the program are ordered to complete the full jail sentence for the crime for which they plead guilty. The program is designed in three stages through which clients progress from high levels of supervision and little independence to low levels of supervision and  high levels of supervision (court reviews, meetings with case worker, therapy sessions, drug testing). The three stages reflect the program’s approach to addiction treatment.  The Plains County Court was very similar to the Oak County court in design. However, it had been operating for less than 6 months, clients were in the early stages of the programme. The main difference with Oak County was that Plains County staffs’ decision making was more collaborative and open to ‘modifying rules’ as they gained experience. | Justice-involved men; Justice-involved women; Judges/sheriffs; Other: case managers, social services coordinator, intake counsellors, administrative staff. | Black, African; African-Caribbean, or African American; Hispanic/Latinx; White; Mixed. | Drugs | Case study | Participant observation, semi-structured interviews, written documents e.g client pamphlets, articles written by staff about the court, websites, newspapers and television reporting including interviews with and references staff who work in this court. | Data not extracted for eligible studies | No |
| Bates 2009 | USA,  Salt Lake City, Utah | To examine drug court through the lens of social role theory and social role valorisation theory in order to understand its effectiveness as a rehabilitative effort for the substance-abusing female offender. | Salt Lake County Felony Drug Court:  A restorative justice alternative to incarceration of the substance-abusing offender. The focus is one of treatment, rehabilitation, potential for relapse, and restoration. The design, less punitive and non-adversarial, is more in harmony with rehabilitation and restoration. The Court team consists of judge, probation, defence counsel, prosecutor, substance abuse treatment provider in a non-adversarial setting. Judge plays central role in monitoring defendant's progress in substance abuse treatment. Formalised and structured treatment interventions are monitored by the drug court judge with frequent court appearances. Graduated sanctions are used to respond to relapses, which incorporate relapse prevention education. Drug courts allow for direct exchanges between the offender and the presiding judge. Drug court's mission is to stop the abuse of alcohol and other drugs and related criminal activity. Offenders submit to frequent and random drug testing, make regular appearances before the judge, are required to attend psychoeducational groups to develop appropriate coping skills and strategies, and are intensely supervised for program compliance. Graduated sanctions, including jail time, are imposed for any program noncompliance. | Justice-involved women | Asian (not specified); Hispanic / Latinx; White. | Alcohol; Drugs | Narrative research | Semi-structured interviews; interviews; Focus groups | Narrative analysis | Yes |
| Belenko 2011 | USA,  The Northeast, Southeast,Midwest, Southwest, and Pacific Northwest USA | To examine screening, selection and admission processes of six representative drug courts in detail, including identifying and analysing the structural and procedural components of the drug courts that relate to the court’s design, targeting, and recruitment process. | Drug court:  Core elements of drug courts include a dedicated case calendar incorporating a central role for the judge, cooperative rather than adversarial roles by prosecution and defence, team decision making, long-term substance abuse treatment, use of sanctions and incentives to manage participant behaviour and enforce court rules, judicial monitoring and direct interaction with the offender, case management and close supervision, and a rehabilitation focus. Six courts from different regions in the United States were selected using four criteria: adult caseload, operational for at least 3 years, had one prior performance evaluation, and program capacity or typical caseload. | Judges/sheriffs; Lawyers; Probation officers or equivalent; Other: Prosecutors; Drug court managers, coordinators or supervisors; Drug court staff; Service providers; Probation, supervision or law enforcement agents; Other informants e.g., faith partners, paralegals, medical staff, pretrial diversion staff. | Not applicable | Drugs | Not reported | Observation e.g. participant observation; Semi-structured interviews, interviews; Other: Written background material | Data not extracted for eligible studies | No |
| Bevli 2018 | USA,  Orange County, California | The purpose of this study was to determine why the implementation of The Substance Abuse, and Crime Prevention Act of 2000 (SACPA),[otherwise known as] Proposition 36 program, is not as successful with the Hispanic residents in Orange County, California. There are 3 research questions looking at supporting and hindering factors and effectiveness in preventing participants from recidivism.  RQ1: What are the factors that support Hispanic substance abusers in completing the Proposition 36 program in Orange County, California?  RQ2: What are the factors that hinder Hispanic substance abusers in completing the Proposition 36 program in Orange County, California?  RQ3: How effective is the Proposition 36 program in preventing Hispanic substance abusers from recidivism? | Proposition 36 program (The Substance Abuse, and Crime Prevention Act of 2000 (SACPA) within a Orange County Drug Court):  Under the program, adults who are convicted of nonviolent first and second time drug possession offenses are given court-supervised drug treatment in the community instead of being jailed. | Justice-involved men; Justice-involved women; Judges/sheriffs; Probation officers or equivalent; Other: Administrators, officers of the court. Justice-involved participants were divided into those who had completed and those who had not completed Proposition program 36. | Hispanic / Latinx | Alcohol; Drugs | Qualitative case study | Semi-structured interviews; interviews | Content analysis / Thematic analysis | Yes |
| Brook  2011 | USA,  New York | To examine the dynamics of drug treatment courts, focusing in part upon this linkage between treatment and punishment, and joining this exploration with an examination of broader structural trends that shape the nature and functioning of the drug court. | Drug court:  Drug courts are widely perceived to offer an alternative to the law-making trends of the 1980s when harsh penalties were established for crimes that were previously considered to be relatively minor in nature. Drug courts have increasingly gained support among many conservatives due in large part to the apparent efficacy of court-supervised treatment, both in terms of direct costs and lowered recidivism rates over incarceration. In most drug courts, “defendants” are required to plead guilty prior to being enrolled in the program, at which point many courts reclassify them as “participants” or even “clients,” a change of terminology that signifies new roles for both the enrollees as well as for the court. Defendants are required to plead guilty prior to being enrolled in the program, at which point many courts reclassify them as participants or even clients, a change of terminology that signifies new roles for both the enrollees as well as for the court. | Judges/sheriffs; Social workers or equivalent; Other: District and Defence attorneys; Court officials; program director; court-affiliated case managers; "court participants" | Not applicable | Drugs | Ethnomethodological | Observation e.g. participant observation; Semi-structured interviews, interviews | Data not extracted for eligible studies | No |
| Burns  2003 | USA, California | To contribute to the existing literature on stigma and stigma management by investigating the processes on how drug court judges and defendants interact to construct the defendant as a personally responsible and rehabilitative changed recovering person, or, alternatively, as an essentially addicted and deficient self. The research also explores broader links between the ways drug courts operate and the potential complications involved in mixing coerced treatment and voluntary participation. | California drug court treatment program:  Hybrid of therapeutic and criminal justice in which the judge becomes a kind of therapeutic administrator, in which the therapist-judge holds the threat of incarceration over the client. The program has three stages. Stage 1: stabilizing defendants, developing a comprehensive treatment plan of therapy, 12-step meetings, and frequent drug testing. Stage 2 continues intensive treatment/less frequent drug testing, vocational and educational support. Stage 3 prepares participants for exiting the program, work skills, education, requiring further meetings and drug tests, and demonstrating achievement of treatment goals. | Justice-involved men; Justice-involved women; Judges/sheriffs; Lawyers; Probation officers or equivalent. | Black, African, African-Caribbean, or African American; Hispanic / Latinx; White | Alcohol; Drugs | Ethnography | Observation e.g. participant observation; Semi-structured interviews, interviews; Other: Televised interviews | Data not extracted for eligible studies | No |
| Butler  2013 | Ireland,  Dublin | Looks at the Dublin pilot drug court as an example of policy transfer between countries and attempts to explore the complexities of policy transfer. | Dublin pilot drug court:  The drug court catered for offenders of 18 years or older who had pleaded guilty or been found guilty of non-violent, drug-related, criminal offences at district court level and who were liable to receive a custodial sentence. Participation in the drug court consisted of three phases lasting for up to 2 years:  Adherence to a rigorous treatment and rehabilitation regime and delivered by the healthcare and educational systems and monitored by the drug court team under the leadership of a designated judge | Judges/sheriffs; Other: senior manager from the Probation Service, consultant psychiatrist, senior civil servant in the Department of Justice, Courts Service Manager. | Not applicable | Drugs | Interview study | Semi-structured interviews, interviews. | Data not extracted for eligible studies | No |
| Crosson  2016 | USA,  Tennessee | This study attempts to explain the relationship that develops and evolves between a judge and offender during the phases of drug court programming. | Drug court program:  Drug court is facilitated by a trained judge and an interdisciplinary team that includes a prosecutor, defence counsel, supervision or probation officer, treatment provider, program co-ordinator and law enforcement representative. The drug court team meets regularly and makes recommendations to the judge on a course of action for each offender. Includes sanctioning program violations with jail time, community service, or additional time in treatment, or rewarding sanctioned behaviour by approving phase progression, open praise in court for an accomplishment, or a reduction in a portion of supervision. It is a non-adversarial environment. The program typically lasts from 12-24 months. | Justice-involved men; Justice-involved women. | Not reported | Alcohol; Drugs | Grounded Theory study | Observation e.g. participant observation; Semi-structured interviews, interviews; Other: Written documents. | Data not extracted for eligible studies | No |
| Csete 2013 | USA,  New York | The purpose of this paper is to investigate in another U.S. state, New York, the practice of drug courts with respect to Methadone Maintenance Therapy (MMT) and the impact of those practices on access to MMT. Few evaluations of drug courts have included the perspective of providers of treatment services, as this study has done. We sought to investigate treatment providers’ understanding of drug courts’ MMT policies and whether MMT providers were regularly consulted by drug court personnel in the disposition of cases of defendants with opioid dependence. | Methadone maintenance therapy (MMT):  Methadone maintenance therapy  (MMT) is recognized by the U.S. Institute of Medicine as well as by the World Health Organization (WHO) to reduce cravings for illicit opioids, reduce crime linked to  drug use, reduce deaths from overdose, reduce HIV risk behaviours, and help patients stabilize their lives and sustain productive activity. Since 2002, buprenorphine has also been authorized for treatment of opioid dependence in the U.S. and is in wide use. Methadone and buprenorphine are included for their therapeutic use on the WHO Model List of Essential Medicines. | Judges/sheriffs; Other: - Treatment providers: described as "directors of clinics" or "senior clinic staff members". Drug court co-ordinators. Methadone patients. | Not applicable | Drugs | Interview study | Semi-structured interviews, interviews; Open-ended survey questions. | Data not extracted for eligible studies | No |
| Datchi 2017 | USA, The Midwest | The aim of the reported analysis is to advance understanding of the unique concerns of diverse women drug treatment court (DTC) participants, in order to improve the problem-solving model of justice and the use of psychological theories in the courtroom. It evaluates the impact of drug treatment court (DTC) interventions. It uses qualitative data collected for a study of family involvement in DTC programming, the aim of which was to identify the legal and social processes that promoted and hindered the participation of family members in the treatment of DTC offenders. | Drug treatment Courts (DTCs):  DTCs adopt a non-adversarial, collaborative approach to justice, where judges, prosecutors, defence counsels, law enforcement, and mental health practitioners form a therapeutic team. They emphasize problem solving, rehabilitation, and accountability; monitor the behaviours and treatment of program participants in regular team meetings and status hearings; and use a system of rewards and sanctions to increase drug offenders’ motivation for change. They operate within a theoretical framework that integrates the concepts of deterrence, therapeutic jurisprudence, and abstinence with the view that addiction is a disease of the brain. DTCs are designed to resolve the underlying causes of crime, shape pro-social attitudes and behaviours, compel drug offenders to enter and stay in treatment, and thus reduce recidivism and promote public safety. | Justice-involved women | Black, African, African-Caribbean, or African American; White. | Alcohol; Drugs | Interview study | Semi-structured interviews, interviews | Critical methodology developed by Carspecken 1996. | Yes |
| Devall 2008 | USA, Midwestern city. | To examine the degree to which drug courts are therapeutic and, therefore, the degree to which they can meet client-participants' basic human needs. | Drug court program:  X County's (pseudonym) drug court has four "tracks" for drug court entry: diversion, sentenced, probation violation, and parole. Diversion track: felony charges pending, have pled guilty to the pending charge(s), but have not been convicted of any crime(s). Diversion track: unsuccessfully discharged from the program and scheduled for formal sentencing, will not have the pending felony charge(s) dismissed. Sentenced track: convicted of at least one felony offense and court-ordered to participate in the drug court program. Probation violation track: also convicted of at least one felony offense and have violated the terms of their probation in some way. Ordered to participate in drug court as a condition of the probation violation sentence. A small number of drug court client-participants participate in drug court as a condition of their release on parole.  X County's drug court: minimum fifteen month, 3-phase program. Phase I: minimum of ninety days. While in Phase I, bi-weekly meetings with case manager, submit to urine screens three times per week, attend individual and group substance abuse counselling (approx four months), complete the 12-step orientation class, attend at least three Alcoholic's Anonymous (AA) and/or Narcotic's Anonymous (NA) meetings per week, appear in court every two weeks, establish a payment plan for restitution (if applicable) and fee ($300). Phase II minimum of six months: attend a minimum of three AA/NA meetings per week, working with an AA or NA sponsor; attend case manager appointments; work at obtaining a high school diploma or GED, if applicable; actively seek employment and/or attend school; urine screens minimum twice per week; make regular payments toward restitution (if applicable) and the Drug Treatment Court fee. Phase II client-participants not employed and/or in school at least 30 hours per week must attend the bi-weekly court review hearings. Disabled clients must attend the bi-weekly court review sessions for the duration of their enrollment in the program, as they are not employed more than 30 hours per week. Advancement to Phase III, must remain clean/sober for a minimum of ninety days, obtain high school diploma/GED (if applicable), and be employed and/or attending school at least 30 hours p/w. If clients relapse in Phase II, are demoted to Phase I and must re-start the program. Phase III: minimum of three months, co same requirements as Phase II, all Phase III client-participants are employed and/or attending school at least 30 hours per week and have obtained their high school diploma/GED. Phase III client-participants must submit to urine screens at least one time per week. | Justice-involved men; Justice-involved women; Judges/sheriffs; Other: Drug court case managers. | Aboriginal / First Nations; Asian (not specified); Black, African, African-Caribbean, or African American; Hispanic / Latinx; White; Mixed; Other: Not specified. | Alcohol; Drugs. | Case study | Observation e.g. participant observation; Semi-structured interviews, interviews; Focus groups; Other: Drug court records (textual analysis). | Data not extracted for eligible studies | No |
| Dickson-Gomez 2022 | USA, Connecticut, Wisconsin and Kentucky | To compare factors that influence the effects of opioid-related laws and policies in Connecticut, Kentucky, and Wisconsin on the transitions from prescription opioids to heroin, fentanyl, and/or injection drug use. This paper uses data from key informants involved in programs to divert people who use drugs (PWUD) from criminal justice involvement, including police, lawyers, judges, and others who work in drug or community courts, and substance use disorder (SUD) treatment providers who received referrals from and worked with police diversion programs or drug courts. | The Sequential Intercept Model (SIM), Police diversion programs (voluntary – not described) and drug treatment courts:  Drug treatment courts are intended to intervene at the next point in the SIM model, when individuals have been arrested and charged. The purpose of these programs is to link non-violent criminal offenders to drug treatment instead of serving prison or jail sentences. | Other: Drug treatment providers, drug court personnel, police, first responders. | Not applicable | Drugs | Interview study | Semi-structured interviews, interviews | Constant comparative approach | Yes |
| Engstrom  2023 | USA,  Binder County | To identify the mechanisms, which a ‘Driving While Intoxicated Court’ within Binder county utilised to reduce recidivism. It also examined the elements of those mechanisms that can be adaptable and adoptable in other courts dealing with Driving While Intoxicated recidivism. | Binder County Driving While Intoxicated Court:  A nonpunitive approach in the criminal system for people with a substance use disorder which provides a comprehensive,  multidisciplinary response to the repeat offender that breaks the cycles of addiction and crime through accountability and improved access to services. | Judges/sheriffs; Other: Community members, Treatment Court Coordinator, Driving While Intoxicated Court Coordinator, Senator, Addiction Technician, Clinical Supervisor, Special Event Coordinator, Social Service Supervisor, Organizer or recovery centre, Director of Family and Spiritual Recovery for recovery centre. | Not applicable | Alcohol | Case study | Observation e.g. participant observation; Semi-structured interviews, interviews; Other: Field experiences, document analysis. | Data not extracted for eligible studies | No |
| Evans 2011 | USA,  Six California counties in four different geographical areas of the state (i.e., Central Valley, Central Coast, Bay Area, and Southern California) | To identify policies and practices that key stakeholders perceive to be most responsible for the successful delivery of court-supervised substance abuse treatment to offenders under Proposition 36. | Substance Abuse and Crime Prevention Act ('Proposition 36 program'):  A criminal justice diversion option that offers community-based substance abuse treatment to nonviolent offenders in lieu of incarceration. Implemented state-wide.  Treatment must be made available to all offenders who meet the conviction-based eligibility criteria despite motivation level or other indicators of program suitability. Proposition 36 specifies that probation supervision and court monitoring occur, but the type and level of supervision and monitoring are unspecified. Incarceration of Proposition 36 offenders for program noncompliance is prohibited. Upon treatment failure, Proposition 36 must provide up to three opportunities for offenders to try treatment again. | Other: Representatives from program administration, probation, the courts, prosecutors, public defenders, treatment providers, and other stakeholders (e.g., parole, program evaluator). | Not applicable | Alcohol; Drugs. | Focus Group study | Focus groups | Data not extracted for eligible studies | No |
| Farringer 2023 | USA, Cincinnati. | This article seeks to fill a gap in  understanding of the day-to-day communication and collaboration practices of the line staff who routinely work with drug court participants as part of the drug court team. Specifically, the present study seeks to (1) identify and describe (a) indicators of, (b)  challenges to, and (c) solutions for effective communication and collaboration among drug court team members and (2) to explore  how collaboration and communication might relate to program effectiveness. | Drug Court Team:  This drug court operates out of the county courthouse as both a pre- and postadjudication court for adults on felony probation. The drug court team is the gatekeeper and the decision-maker. The team is ultimately responsible for the day-to-day operations and enforcement of all other key components.  The team should be comprised of the judge, prosecutor, defense attorney, community supervision (i.e., probation) officer, court administrator, and treatment provider(s). Recommendations also include having precourt team meetings and ongoing training for all team members on issues relevant to the court clients. Most  importantly, however, teams are encouraged to share information and engage in shared decision-making. | Judges/sheriffs; Probation officers or equivalent; Other: Administrative director, defence attorneys, director of residential treatment program, clinical supervisor, clinical service providers. | Not applicable | Drugs | Interview study | Semi-structured interviews, interviews; Open-ended survey questions. | Data not extracted for eligible studies | No |
| Fischer 2007 (linked with Fischer 2009 | USA, Northern California | This study looked at current and past experiences of female drug-court participants to assess their program and envision future program innovations. (Not explicitly stated) | Northern California drug-court program:  After individual assessment by the  case manager, clients are provided with resources and referrals to various treatment facilities. Clients progress through three phases each lasting for 6 months, and after-care that lasts approximately 6 months. Participants are urine and Breathalyzer-tested twice a week, with the days of the test selected at random. Continuous  graduated intensive supervision and monitoring for compliance with treatment are made possible by the joint collaboration of a judge, three probation officers, 12 treatment providers, two case managers, and two counsellors. A mental-health specialist-court liaison plays the dual role of case manager and liaison, evaluating and reporting to the judge the client’s progress. The client appears in court three times a week in phase one, twice a week in phase two, and once a week in phase three. Satisfactory progress is symbolized by advancement through the phases. Compliance with the program requirements leads to a decrease in the intensity of supervision and to greater autonomy and individual choice. Graduated sanctions for noncompliance range from explanation and planning, to being dropped to a previous stage or remaining longer in a phase, to being sent to jail. | Justice-involved women | Not reported | Drugs | Action research - appreciative collaborative inquiry. | Semi-structured interviews, interviews. | Content analysis | Yes |
| Fischer 2009 | USA, North Carolina | The purpose of this study was to measure the current levels of moral development  and examine and discover the phenomenological essences of the experiences that occur as attorney members of the drug court become involved as therapeutic agents in the drug court process.  RQs:  1. What are the stages of moral development of attorneys practicing in drug treatment court?  2. What are the perceptions, attitudes, emotions, and beliefs about the court and the criminal justice system of attorneys practicing in adult drug court?  3. What cognitive meaning do attorneys practicing in adult drug court give to their perceptions, attitudes, emotions, and beliefs about the court and the  criminal justice system? | Drug Court:  Specifically, the primary role and moral development of attorneys working within drug courts.  Drug courts: Through a rigorous process involving regular court appearances, random drug testing, required treatment, monitored activities, and attendance at recognized recovery programs, over a period of 12-18 months, the court applies a therapeutic adjudication to break the cycle of addiction and criminal behaviour. The process involves a hands-on judiciary that becomes involved in all aspects of a client’s journey through the  program (Goldkamp 2000). | Other: Attorneys (prosecuting and defence). | Not applicable | Drugs | Interview study | Semi-structured interviews, interviews. | Data not extracted for eligible studies | No |
| Francis 2014 | USA, Florida | The overarching question for the study was: Do non completers of drug court programs report any benefits from the intervention? Additional questions include: Does the drug court experience provide both successful and unsuccessful clients with future positive alternatives? and Are non completers functioning better compared with before they entered the drug court program? | Drug Court programme:  Two programmes were researched, "Pretrial Intervention" (PTI) and the post-plea "Expanded and Enhanced Substance Abuse Treatment" (EESAT).  Offenders involved in the criminal  justice system with admitted substance abuse problems are often referred to drug court where they are offered drug treatment and judicial monitoring in lieu of incarceration or traditional probation.  The Pretrial Intervention (PTI) program uses dismissal of criminal charges to entice individuals to participate in drug court.  The "Expanded and Enhanced Substance Abuse Treatment" (EESAT) program uses threat of incarceration as leverage to influence people to attend the program.  Though drug court programs demonstrate positive results overall in the primary goals of reductions in reoffending and drug use, typically only successful client outcomes are tracked. As a result, little is known about the results of so-called “unsuccessful” clients. | Justice-involved men; Justice-involved women; Other: Only the majority category of sample reported. | White (only majority category of sample reported). | Alcohol; Drugs | Grounded theory study | Semi-structured interviews, interviews. | Grounded theory | Yes |
| Fulkerson 2012 | USA, Arkansas | This article is a qualitative study on the perceptions of the drug court program of those drug court participants who have graduated or have been terminated from an Arkansas drug court program. The comparison will allow the court to determine whether any of the drug court programs and practices should be modified, expanded, or eliminated (aim not explicitly stated). | Greene County Drug Court:  The drug court follows a team approach that includes the drug court judge, prosecuting attorney, defence attorneys, probation officer, counsellor, and administrative assistant as members of the team. After acceptance into the program, participants meet the probation officer on a regular basis, take part in individual and group counselling, and attend mandatory alcoholic anonymous (AA) meetings. The participants are also subjected to frequent random drug screening.  The Greene County Drug Court also uses a phased program that requires 15 to 18 months to complete. The program has four identifiable phases. During each phase, participants are required to take part in individual and group counselling sessions, attend 12-step program meetings, submit to random, but regular urinalysis, and appear before the drug court for progress reports (Greene County Drug Court, 2006). | Other: 'Drug court participants' (graduates and non completers). Gender of participants was not reported. | Not reported | Alcohol; Drugs | Phenomenological study | Semi-structured interviews, interviews | Phenomenology e.g.IPA | Yes |
| Gallagher 2013 | USA, Fort Worth, Texas | To explore the factors that might contribute to racial disparities in drug court outcomes and discuss implications for social policy advocacy, future social science research, and social work practice.  Research Question: How do African American participants in the D.I.R.E.C.T. (Drug Impact Rehabilitation Enhanced Comprehensive Treatment) drug court program view this program in what they regard as the most helpful aspects of the program, the challenges they face in participating in the program, and how the program can be improved? | Drug Impact Rehabilitation Enhanced Comprehensive Treatment  (D.I.R.E.C.T.) program within an adult drug court:  The D.I.R.E.C.T. program provides services to males and females,  17 years of age and older, who have been arrested for a misdemeanor or felony that is related to substance abuse. Once a participant graduates from the D.I.R.E.C.T. program, his or her criminal case is dismissed. The length of the program ranges from 6 to 24 months based on whether  the criminal offense is a misdemeanor or felony and a participant’s progress on meeting his or her individualized goals. | Justice-involved men; Justice-involved women | Black, African, African-Caribbean, or African American | Alcohol; Drugs | Interview study | Semi-structured interviews, interviews | Data not extracted for eligible studies | No |
| Gallagher, Marlowe & Minasian 2019  (linked to Gallagher, Estreet, Nordberg, Zongrone, Minasian and Symanowski 2023; Gallagher, Wahler, Minasian, & Edwards 2019; Gallagher, Nordberg, Francis, Menon, Canada& Minasian 2021). | USA, Midwestern state | Medication Assisted Treatment (MAT) may not elicit incremental benefits beyond the intensive backdrop of services or may require substantial modifications to work effectively in the drug court environment. The current study sought to shed light on this issue by interviewing drug court participants with opioid use disorders about their experiences with MAT and other services in the program, and their impressions as to why MAT might not be achieving its potential in drug courts. | Medication Assisted Treatment in a Drug Court:  Medication-assisted treatment (MAT) using three Food and Drug Administration-approved addiction medications—methadone, buprenorphine, and naltrexone—has been demonstrated to improve outcomes for persons with opioid use disorders on probation or parole and in traditional substance use treatment programs. | Justice-involved men; Justice-involved women | White; Other (not specified) | Drugs | Focus Group study | Focus groups | Data not extracted for eligible studies | No |
| Gallagher, Wahler, Minasian & Edwards 2019 (linked to  Gallagher, Marlowe & Minasian 2019) | USA, Midwestern State | Research question: What are drug court participants’ perceptions on the most helpful aspects of drug court in treating their opioid use disorders, how the drug court could be more helpful in treating their opioid use disorders, and their thoughts and experiences on the use of medication-assisted treatments (MATs) to support recovery? | Drug Court:  The drug court for this study treats individuals who have been arrested for nonviolent offenses and have substance use disorders. Drug court participants are, most commonly, arrested for possession of a controlled substance, possession of drug paraphernalia, and acquiring possession of a controlled substance by fraud and theft. Additionally, participants are accepted into drug court based on evidence that their drug use, or substance use disorder, was associated with their criminal activity (e.g., forging prescriptions to gain access to opioids for personal use). A key aspect of drug court that differs from traditional criminal justice interventions (e.g., probation) is that drug courts employ a multidisciplinary judicial team.  With MATs, opioid agonist medications are prescribed and closely monitored to control the level of medication ingested, thereby reducing drug-seeking behaviours and intense highs and withdrawal periods associated with illicit drug use. | Justice-involved men; Justice-involved women | White; Other: ‘Non-white.’ | Drugs | Phenomenological | Focus groups | Phenomenology e.g.IPA | Yes |
| Gallagher, Estreet, Nordberg, Zongrone, Minasian, Szymanowski, S 2023  (linked to  Gallagher, Marlowe & Minasian 2019) | USA, (region not reported) | Research Question (aim not stated): What are women’s thoughts, opinions, and experiences in drug court, in regard to the quality of treatment they received for their opioid use disorders and to the use of MAT in drug court programming to treat opioid use disorders? | Drug courts and medication assisted treatment:  Drug courts promote recovery from substance use disorders, offering substance use disorder treatment, and other supportive interventions as an alternative to incarceration and reducing criminal recidivism rates. Medications are often combined with substance use disorder counselling and are commonly called medication-assisted treatment. There is strong evidence that MAT is an effective  intervention in treating opioid use disorders for those who are involved in the criminal  justice system, including parolees and probationers (Gordon et al., 2015). | Justice-involved women | Not reported | Drugs | Focus Group study | Focus groups | Data not extracted for eligible studies | No |
| Gallagher 2021  (linked to  Gallagher, Marlowe & Minasian 2019; Gallagher, Wahler, Minasian, Edwards 2019; Gallagher, Estreet, Nordberg, Zongrone, Minasian, Szymanowski, 2023). | USA Baltimore | Research questions: -What are drug court team members’ perceptions on how drug court treats participants who have opioid use disorders?  -What are drug court team members’ perceptions on how drug court utilizes medication-assisted treatment (MAT) to treat participants who have opioid use disorders? | Medication-assisted treatment (MAT):  MAT is the combination of counselling and medications approved by the Federal Drug Administration (FDA) to treat opioid use disorders. Currently, the FDA has approved three medications to treat opioid use disorders, including methadone, buprenorphine, and naltrexone. Research on MAT has demonstrated that the combination of counselling and medication significantly reduces opioid use and associated risks, including overdose, death, infectious disease transmission, and other diseases associated with injection drug use (Substance Abuse and Mental Health Services Administration, 2018). | Judges/sheriffs; Social workers or equivalent; Probation officers or equivalent; Other: Prosecuting attorney, drug court coordinator, addictionologist, sober living case manager, recovery coach, defence attorney, treatment providers, drug court case managers. | Not applicable | Drugs | Focus Group study | Focus groups | Data not extracted for eligible studies | No |
| Gallagher & Wahler 2018  (linked to Gallagher, Nordberg, Dibley 2019; Gallagher, Nordberg, Deranek, Minasian, 2019). | USA, Midwestern State | To develop an in-depth understanding of factors that might contribute to racial disparities in drug court outcomes. | Drug Court:  Drug courts are a unique criminal  justice intervention that focus on rehabilitation for individuals who have a substance use disorder, while also balancing public safety. Drug courts are conceptualized by their 10 key components, which are designed to enhance motivation for change through a strengths-based, non-adversarial approach. | Justice-involved men; Justice-involved women | Black, African, African-Caribbean, or African American. | Alcohol; Drugs | Phenomenological | Open-ended survey questions | Phenomenology e.g. IPA | Yes |
| Gallagher, Nordberg, Deranek & Minasian 2019 (linked to Gallagher & Wahler 2018; and Gallagher, Nordberg, Dibley 2019). | USA, Midwestern State | Research question: What are African American women’s views on the most helpful aspects of drug court and how drug court could be more helpful in supporting them in graduating the program? | Drug Court:  Description not reported. | Justice-involved women | Black, African; African-Caribbean; African American. | Alcohol; Drugs | Evaluation | Open-ended survey questions | Data not extracted for eligible studies | No |
| Gallagher, Nordberg & Dibley 2019  (Linked to Gallagher & Wahler 2018; Gallagher, Nordberg, Deranek, Minasian 2019) | USA,  Midwestern State | This study adds to the knowledge base by using qualitative methods to explore the factors that may contribute to racial disparities in drug court outcomes. | Drug Court:  Description not reported. | Justice-involved men; Justice-involved women. | Black, African, African-Caribbean, or African American. | Not reported. | Phenomenological. | Open-ended survey questions. | Phenomenology e.g. IPA | Yes |
| Gallagher, Nordberg & Lefebvre 2017 (linked with Gallagher, Nordberg, & Gallagher 2018) | USA, Midwestern State | Research question: How do drug court participants view the program, in regards to the most helpful aspects that support them in graduating the program and how the program could be more helpful in supporting them in graduating the program? | Drug Court:  Drug courts focus heavily on utilizing the criminal justice system as an avenue to treat substance use disorders, relying on coercive treatments and having treatment providers in courts, probation offices, and prisons, for instance. | Justice-involved men; Justice-involved women | Black, African, African-Caribbean, or African American; Hispanic / Latinx; White. | Alcohol; Drugs | phenomenological | Open-ended survey questions | Data not extracted for eligible studies | No |
| Gallagher, Nordberg & Gallagher 2018 (linked with Gallagher, Nordberg, Lefebvre 2017) | USA, Midwestern State | Research questions: - How do participants of a drug court view the program in regard to the most helpful aspects that support them in graduating from the program?  - How could the program be more helpful in supporting them in graduating from the program? | Drug Court:  Drug courts utilize interdisciplinary teams of judges, attorneys, probation officers, and clinicians to oversee substance abuse treatment. The model reduces the adversarial nature of traditional criminal courts yet still relies on monitoring of abstinence and the issuance of sanctions and rewards. | Justice-involved men; Justice-involved women. | Black, African, African-Caribbean, or African American; Hispanic / Latinx; Mixed; White. | Alcohol; Drugs | Phenomenological | Open-ended survey questions | Data not extracted for eligible studies | No |
| Gallagher & Nordberg 2017 | USA, Midwestern state | To learn the lived experiences of women in a Midwestern drug court program with a particular focus on the aspects of the program that support them in graduating and how the program can be improved. | Drug Court:  Drug courts are designed to help those with substance use disorders and to reduce criminal recidivism associated with illicit drug use. They are judicially supervised court dockets that usually offer treatment to non-violent drug-addicted offender. | Justice-involved women | Black, African, African-Caribbean, or African American; Hispanic / Latinx; White. | Drugs | Qualitative | Semi-structured interviews, interviews | Data not extracted for eligible studies | No |
| Gallagher, Nordberg & Gallagher 2017 | USA, Midwestern State | To develop an in-depth understanding of a Midwestern veterans treatment court (VTC) by asking participants their views on the most helpful aspects of the program and how the program could be more helpful to support the in graduating from the VTC program | Veteran’s Treatment Court:  Rehabilitative approach to criminal justice, focusing on treatment for mental health and substance use disorders. | Veterans who use drugs &/or alcohol & have committed a crime. | Aboriginal/ First Nations; Black, African, African-Caribbean, or African American; White. | Alcohol; Drugs | Not reported | Open-ended survey questions | Data not extracted for eligible studies | No |
| Gallagher 2015 | USA, region not reported | Research question: How do drug court participants view the program, regarding the quality of substance abuse counselling they receive, the supportiveness of the drug court team, the effectiveness of  sanctions and incentives, the effectiveness of frequent contact with the judge, and the effectiveness of frequent and random drug tests? | Drug Court:  This study used the Drug Court 10 Key Components (Table 1, p66) to describe the drug court but stress that there are many variations on the key components (National Association of Drug Court Professionals (1997). | Justice-involved men; Justice-involved women | Black, African, African-Caribbean, or African American; Hispanic / Latinx; White. | Alcohol; Drugs | Phenomenological | Semi-structured interviews, interviews | Data not extracted for eligible studies | No |
| Gallagher, Nordberg, Szymanowski & Malone 2019 | USA, Indiana | Research question: How do drug court participants view the program, regarding the quality of substance abuse counselling they receive, the supportiveness of the drug court team, the effectiveness of sanctions and incentives, the effectiveness of frequent contact with the judge, and the effectiveness of frequent and random drug tests? | Monroe County Drug Court:  The (first drug court) Miami drug court diverted people before the court away from the traditional judicial route and into the court-centred, rehabilitative treatment paradigm. By accepting this diversion, drug court participants agreed to plead guilty, remain drug-free, participate in periodic drug testing, follow treatment recommendations, and report to drug court for supervision. | Justice-involved men; Justice-involved women | Hispanic / Latinx; White | Alcohol; Drugs | Phenomen-ological | Open-ended survey questions | Data not extracted for eligible studies | No |
| Garcia 2019 | Canada, Vancouver | To examine the ways health and the social determinants of health are engaged and framed externally with regard to Vancouver’s Downtown Community Court functioning, as well as internally by Downtown Community Court actors. | Vancouver’s Downtown Community Court:  In September 2008, Canada’s first community court, the Downtown Community Court (DCC), was launched to address individuals’ needs and circumstances leading to criminal behaviour. The Court was created through a partnership between Canada’s Ministry of Justice, the Provincial Court of British Columbia, and 14 health and social services agencies subsequently co-located in the courthouse. The DCC was created as a purpose-designed, community based problem-solving court that uses an intersectoral, multi-stakeholder approach to address the needs and circumstances of the individuals (‘clients’) who appear before it. Where deemed appropriate, judges employ diverted sentencing including community service alongside a personalized plan that may include housing support, social service access, employment counselling and links to culturally appropriate services. | Justice-involved men; Justice-involved women | Not reported | Drugs | Case study | Observation e.g. participant observation; Other: Analysing court evaluation reports and newsletters, examining press media representation of the court. | Thematic; Other: Content analysis of documents and print media; thematic coding of court observations. | Yes |
| Goldberg 2019 | USA, region not reported. | The aim of this study is to gain a deeper understanding of the intersection of family relationships with motivation of women in Drug Treatment Court (DTC) to attain their health goals (from Abstract). | The WISH (Women Initiative Supporting Health) intervention Drug Treatment Court:  Included approximately biweekly 1 hour manualized sessions. The purpose of the sessions was to address women’s health, defined broadly to include physical health, mental health, substance use, and safety from IPV (Intimate Partner Violence). | Justice-involved women | Not reported | Alcohol; Drugs | Interview study | Semi-structured interviews, interviews | Data not extracted for eligible studies | No |
| Hamilton 2019 | USA, Philadelphia | To elaborate on the quantitative findings (of this study) to provide context for how and why participants were or were not engaging with their mandatory treatment | Philadelphia Treatment Court and Philadelphia’s Adult Probation and Parole Department:  A pre-trial diversion program operating within the Philadelphia Municipal Court and Court of Common Pleas. To participate in the Philadelphia Treatment Court, offenders are required to plead 'nolo contendre' and have their plea held in abeyance until they successfully complete the requirements placed on them by the Treatment Court. These requirements always include drug treatment. | Justice-involved men; Justice-involved women | Black, African, African-Caribbean, or African American; Hispanic / Latinx; White; Mixed. | Alcohol; Drugs | Mixed methods - quantitative survey and subsequent qualitative research | Semi-structured interviews, interviews | Thematic; Other: Deductive thematic analysis using a structured a priori coding scheme. | Yes |
| Hardy 2005 | USA, California | To gain an in-depth understanding of stakeholders' experience with Substance Abuse and Crime Prevention Act and examine issues that arose during the first year of implementation. | Substance Abuse and Crime Prevention Act:  Criminal justice policy toward community-based, court-monitored treatment for drug-involved offenders. Proposition 36 was enacted into law as the Substance Abuse and Crime Prevention Act (SACPA). Adult defendants, probationers, and parolees convicted of simple drug possession or drug-use offences and otherwise eligible for SACPA could now be sentenced to probation with drug treatment instead of either probation without treatment or incarceration. After completion of treatment, defendants may petition the sentencing court to set aside the conviction. Probationers or parolees who violate drug-related conditions of their release may also receive treatment instead of incarceration. | Judges/sheriffs; Lawyers; Social workers or equivalent; Probation officers or equivalent; Other: Representative of program administration | Not applicable | Alcohol; Drugs | Evaluation including focus groups and a survey. | Focus groups | Data not extracted for eligible studies | No |
| Harrell 1998 | USA, Washington | To examine the use of judicial monitoring, frequent drug testing, treatment and graduated sanctions, and early intervention with drug involved defendants in Washington, D.C. by assessing (through process evaluation) services and interventions, policies, practices, and procedures, how well the programmes performed, role of the judge/judicial personnel, views and opinions of defendants, and an impact evaluation that assessed 1) reduced drug use and criminal activity, 2) increased voluntary participation in drug treatment or aftercare following program participation, and 3) improved the economic and social functioning of participants in the year after the program. | Superior Court Drug Intervention Program:  The Superior Court Drug Intervention Program (SCDIP), was an experimental court-based intervention for drug-involved defendants, based on a comparison of the drug use and criminal activity of drug felony defendants who were randomly assigned to one of three dockets. The sanctions docket offered drug-involved defendants a program of graduated sanctions with weekly drug testing, referrals to community-based treatment, and judicial monitoring of drug use. The treatment docket offered drug-involved defendants weekly drug testing and an intensive court-based day treatment program. The standard docket offered drug-involved defendants weekly drug testing, judicial monitoring, and encouragement to seek community-based treatment programs. Key features of the SCDIP include 1) early intervention, 2) judicial involvement in monitoring defendant progress in the program, 3) frequent drug testing, and 4) immediate access to information on defendant drug use. | Justice-involved men; Justice-involved women; Judges (one per docket) | Not reported | Drugs | Process & Impact evaluation | Focus groups; semi-structured interviews | Not reported | Yes |
| Hennessy 2023 | USA, region not reported | To learn about the process of adapting and integrating a standardized recovery capital measurement and recovery planning intervention with two drug treatment courts in the United States | REC-CAP system:  Recovery-capital oriented system of measurement, planning, and engagement in two drug treatment courts. The REC-CAP uses the Assessment of Recovery Capital (ARC) scale consisting of sub-scales to measure personal recovery capital and social recovery capital, indicators of well-being, including commitment to sobriety; community recovery capital, barriers to recovery, perceived level of support, and quality of life. | Other: Court staff | Not applicable | Alcohol; Drugs | Case study | Semi-structured interviews, interviews; Other: Adapted Texas Christian University Work Experience and Workshop Evaluation Surveys. | Data not extracted for eligible studies | No |
| Horowitz 2023 | USA, Selby | To demonstrates how gender and race create differentiated and unequal rehabilitative projects. | The Selby Drug Court:  Program of four phases strongly shaped by alcoholics anonymous understanding of recovery. Treatment includes alcoholics or narcotics anonymous meetings and drug testing. Violations or unsatisfactory participation often sent clients back to earlier phases. | Justice-involved men; Justice-involved women; Judges/sheriffs; Lawyers; Social workers or equivalent; Probation officers or equivalent | Black, African African-Caribbean, or African American; White. | Alcohol; Drugs | Ethnography | Observation e.g. participant observation; Semi-structured interviews, interviews | Data not extracted for eligible studies | No |
| Horrocks 2004 | UK, region not reported | To explore the recently introduced initiative of coercive treatment for drug misuse in the UK. | Drug treatment and testing order:  A community based sentence introduced in the Crime and Disorder Act 1998. Provides the court with the power to impose cooperation with a treatment programme. Offenders choose between a custodial sentence or a community sentence with enforced treatment. Requires the offender to undergo treatment for a set period of between 6 months and 3 years, tested regularly for drug use, monitor treatment compliance and attend court review hearings where progress is evaluated. | Justice-involved men; Justice-involved women | Not reported | Drugs | Not reported | Semi-structured interviews, interviews | Data not extracted for eligible studies | No |
| Kahn 2023 | USA, New York | The present study explores how individual drug treatment court team members responded to COVID-19 mitigation requirements and adapted and incorporated remote technology into their day-to-day operations. | Drug Court (using virtual technology during COVID 19 pandemic):  To reduce/eliminate face-to-face sessions and meetings drug courts used videoconferencing software and other forms of electronic communications (e.g., e-mail, text) for judicial processing and provision of services to clients. | Judges/sheriffs; Other: Case managers, counsellors, agency managers, court coordinator, peer recovery specialists (people in recovery from substance use disorder who help clients by providing supports and linking them to services). | Not applicable | Not reported | Interview study | Semi-structured interviews, interviews; Other: Interviews structured using constructs from Normalisation Process Theory. | Data not extracted for eligible studies | No |
| Kennedy-Hendricks 2021 | USA, Urban, rural, and suburban settings | To explore the perceptions on the structural and organizational factors shaping access and use of medication treatment for opioid use disorder in community supervision. | Medication assisted treatment for opioid use disorder:  Substance use treatment like buprenorphine, and methadone, for individuals under community supervision with opioid use disorder to reduce overdose risk and facilitate recovery. | Probation officers or equivalent; Other: Addiction treatment officials. | Not applicable | Drugs | Interview study | Semi-structured interviews, interviews | Thematic | Yes |
| Kerr 2011 | UK, England; Other: Wales | The process study had two aims. Firstly, to map the implementation, operation and core elements of the Dedicated Drug Court (DDC) model. This included identifying and exploring any variations between the model operating at the six different DDC sites in England and Wales.  The second aim was to identify the factors affecting the perceived impact of the DDC model by exploring the  influences that underpinned its potential to reduce drug use and associated offending.  However, this study was not aiming to measure impact in any way. | Dedicated Drug Court (DDC):  DDCs were initially based on 4 features (first initiated in the USA):  -Increasing the offender’s likelihood of successful rehabilitation through early, continuous, and intense judicially supervised treatment.  -Mandatory periodic drug testing.  -Community supervision.  -The use of appropriate sanctions and other rehabilitation services.  Figure 1.1 (p. 2) provides overview of DCC from arrest to completion. | Justice-involved men; Justice-involved women; Probation officers or equivalent; Other: Court staff, judiciary, treatment provider. | White; ‘Other' | Alcohol; Drugs | Interviews and focus group study. | Semi-structured interviews, interviews; Focus groups | Thematic | Yes |
| Kouimtsidis  2007 | England, Hertfordshire | To generate data that will explore the dilemma of treatment or prison from the service user and staff perspectives. | Drug treatment and testing order:  Since October 2000 drug treatment and testing order services have been implemented throughout the UK. In 2005 the drug treatment and testing order was replaced by the drug rehabilitation requirement. Another multi-agency service, the drug intervention programme, was implemented the same year. The drug treatment and testing order concept of either treatment or prison is designed to facilitate treatment access for individuals trapped in the vicious circle of crime to fund drug use. | Justice-involved men; Justice-involved women; Probation officers or equivalent; Other: Drug counsellors, consultant psychiatrist | Not reported | Drugs | Other: Qualitative study | Semi-structured interviews, interviews; Other: Unstructured group interviews | Grounded Theory | Yes |
| Larsen 2014 | USA, Central Carolina | This study aims to answer the question: do pre-deployment, combat, and  post-deployment traumas predict veteran's symptom levels at intake to a Veteran's Treatment Court (VTC)? | Veteran Treatment Court:  VTCs are a hybrid of drug and mental health courts, which promote sobriety, recovery, and stability through a coordination of responses involving community collaboration with service providers, the Department of Veterans Affairs health care networks, the Veterans Benefits Administration, State Departments of Veterans Affairs, volunteer veteran advocates, and veterans family support organizations. | Justice-involved men; Justice-involved women | Black, African African-Caribbean; African American; Hispanic / Latinx; White; Other: American Indian. | Alcohol; Drugs | A quantitative descriptive, ex post facto design study. It focuses  on between-group differences between participants who self-identified veterans and non-veterans. | Open-ended survey questions; Other: Measures of depression and PTSD, alcohol and drug abuse severity, employment problems, post-deployment trauma. | Data not extracted for eligible studies | No |
| Lutze 2014 | USA, Benton & Franklin counties, Washington. | The current study expands upon existing process evaluations by giving a detailed description and analyses of the product resulting from implementing the Ten Key Components in a successful adult drug court (not explicitly stated). | The Benton and Franklin Counties Adult Drug Court (BFADC):  Participants were held accountable for their behaviour and drug court compliance through the following practices: Weekly court appearances, randomized drug testing, confirmation by providers of participant’s treatment compliance, confirmation and monitoring by case managers, trackers, and the police of participant’s compliance to court imposed requirements. Immediate sanctions for noncompliance such as jail, work crew, etc. | Justice-involved men; Justice-involved women. | Black, African, African-Caribbean, or African American; Hispanic / Latinx; White. | Alcohol; Drugs | Process evaluation | Other: data were collected for each drug court participant for phase duration, treatment participation, incentives and sanctions, drug testing, and payment of court fees. | Data not extracted for eligible studies | No |
| Lyons 2014 | Canada, Ottowa | To explore how participants in drug treatment orders are constructed as addicted subjects. | Drug Treatment Court:  Drug treatment court participants must undertake a comprehensive treatment programme and numerous bail conditions such as observing curfews, abstaining from drugs and alcohol, submitting to random urinalysis tests and participating in treatment to the satisfaction of the treatment providers. | Justice-involved men; Justice-involved women | Not reported | Not reported | Critical ethnography | Observation e.g. participant observation; Semi-structured interviews, interviews; Other: informal conversation and textual analysis of policy documents, | Data not extracted for eligible studies | No |
| Mackinem 2007 | USA, south eastern state | To explore how staff members react to clients’ responses when confronted with positive tests for illicit drug use (Not explicitly stated). | Drug Court:  Drug court is composed of three elements: treatment services, drug testing, and judicial supervision. Treatment services include drug counselling, self-help meetings, such as Narcotics Anonymous, and other services, such as vocational and educational counselling. Staff members test clients for drugs, often several times per week. In drug-court sessions, the staff reviews with the drug-court judge the clients’ performance, reporting on the clients’ drug-test results, attitude, and attendance at counselling and self-help meetings. | Judges/sheriffs; Other: Public defender, drug counsellor, program coordinator, recovering addict. | Not applicable | Drugs | Not reported | Other: Secondary data from interviews, field notes, court sessions and "a wide variety of documents". | Data not extracted for eligible studies | No |
| Mackinem 2010 | USA, Metropolitan region of a state capital in a south-eastern state: one in an urban county, another in a suburban county, and the third in a farming county. | To examine how staff contributes to the operations of an adult drug court and how staff produces client failures through their decisions, conversations, and actions. | Drug Court:  Drug court offers treatment services (counselling, self help, drug testing, judicial supervision). Judges may punish noncompliant clients (advised by court team) with sanctions that range from community service to jail time. | Judges/sheriffs; Lawyers; Social workers or equivalent; Other: Program coordinators. | Not applicable | Drugs | Ethnography | Observation e.g. participant observation; Semi-structured interviews, interviews; Other: Drug court documents, applications for drug court, and court notes from counsellors. | Data not extracted for eligible studies | No |
| Maddox 2023 | USA, Northeast states | To understand how participants in the diversion court program feel about their experiences. The purpose of the study is to analyse responses from drug treatment court participants on their recommendations to improve drug treatment court. | Drug Treatment Court:  Key components: integrated alcohol and other drug treatment services while their justice case is processing. A non-adversarial approach. Identifying participants that are eligible for drug treatment court as early as possible. Provide continual substance use treatment and other related services. Frequent substance testing. Addiction is treated as an ongoing problem, where relapsing is common. Monitoring and evaluating the participant’s achievement. Partnerships of drug courts and various organizations associated with cession of drug use. | Justice-involved men; Justice-involved women. | Black, African, African-Caribbean, or African American; Hispanic / Latinx; Pacific Islander; White; Mixed; Other: Cape Verdean. | Drugs | Secondary qualitative analysis | Other: Secondary analysis of semi-structured interviews | Grounded theory; Other: secondary analysis | Yes |
| Eley 2002  (linked with McIvor 2006 & 2009) | UK, Scotland,  Glasgow | To assess how effective the Drug Court is in reducing the level of drug related offending and reducing or eliminating offenders’ dependence on or propensity to use drugs; and to determine whether the operation of the Drug Court is viable within the Scottish context. | Glasgow Drug Court:  The Drug Court can impose Drug Treatment and Testing Orders (DTTOs), Probation Orders with a Condition of Drug Treatment, concurrent DTTOs and Conditional Probation Orders, and deferred sentences.  The objectives of the Drug Court are to:  - reduce the level of drug-related offending behaviour;  - reduce or eliminate offenders’ dependence on or propensity to use drugs; and  - examine the viability and usefulness of a Drug Court in Scotland using existing  legislation, and to demonstrate where legislative and practical improvements might be important.  The proposed target group for the Drug Court is offenders aged 21 years or older of both sexes, in respect of whom there is an established relationship between a pattern of serious drug misuse and offending and whose drug misuse is susceptible to treatment. Offenders referred to the Drug Court must otherwise have been facing prosecution in the Sheriff Summary Court and should normally first appear before the summary court from custody. | Justice-involved men; Justice-involved women; Judges/ sheriffs; social workers or equivalent; Other: Drug Court Co-ordinator and the Drug Court Procurator Fiscal. Police officers. Defence agents. Drug Court Supervision and Treatment Team: -addiction workers, including a supervisor. Social work managers, two nurses, a medical officer and a senior medical officer. | Not reported. | Drugs | Formative and process evaluation | Observation e.g. participant observation; Semi-structured interviews, interviews; Other: Drug court records. | Thematic analysis | Yes |
| McIvor 2009 | UK Scotland, Glasgow & Fife | The focus of this study is upon the role of judicial  involvement in the ongoing review of Drug Court participants’ progress, specifically the involvement of Sheriffs in overseeing the progress of offenders made subject to orders. | Scottish Drug Courts:  Sentences imposed can include drug treatment and testing orders, probation orders (with or without additional requirements) and deferred sentences. The majority of orders made in the first two years of the pilot were drug treatment and testing orders which involved regular drug testing, emphasised the case management role of the supervising officer, who would be responsible for co-ordinating service provision, and included provision for sentencers to take an active role in reviewing the progress of offenders. | Justice-involved men; Justice-involved women; Judges/sheriffs; social workers or equivalent; Other: Addiction workers, nurse and medical officers and, in Glasgow, the Drug Court Co-ordinator and Procurator Fiscal. | Not reported | Drugs | Not reported | Observation e.g. participant observation; Semi-structured interviews, interviews. | Thematic | Yes |
| McIvor 2006 | UK Scotland, Glasgow & Fife | To assess how effective the Drug Courts are in reducing the level of drug-related offending and reducing or eliminating offenders’ dependence on or propensity to use drugs; and determine whether the operation of the Drug Courts is viable within the Scottish context. | Glasgow Drug Court and Fife Drug Court:  The drug courts aim to reduce the level of drug-related offending behaviour, to reduce or eliminate offenders’ dependence on or propensity to use drugs and to examine the viability and usefulness of a Drug Court in Scotland. All Orders made by the Drug Court Probation Orders or Drug Treatment and Testing Orders (DTTOs) are subject to drug testing (urinalysis) and regular (at least monthly) review. The services included counselling, prescribing, access to day programmes and primary medical care. Substitute prescribing (using methadone) constituted the core element of the treatment service in practice. | Justice-involved men; Justice-involved women; Judges/sheriffs; Social workers or equivalent; Other: Procurators Fiscal; defence agents; police officers; addiction workers; social work managers; nurses; a medical officer; social work assistants; administrative/secretarial staff; Drug Court Co-ordinator; the designated Sheriff Clerk. | Not reported | Alcohol; Drugs | Outcome evaluation | Observation e.g. participant observation; Semi-structured interviews, interviews; Other: informal conversation and textual analysis of policy documents. | Not reported | Yes |
| McPherson 2013 | Not reported (pseudonym used) | To investigate how actors from different institutional and professional backgrounds employ logical frameworks - the logics of criminal punishment, rehabilitation, community accountability, and efficiency - in their micro-level interactions and thus how logics affect day-to-day organizational activity. | Drug Court:  Alternative judicial process for legal offenses related to drug use. | Judges/sheriffs; Lawyers; Social workers or equivalent; Probation officers or equivalent. | Not applicable | Not reported | Ethnography | Observation e.g. participant observation; Semi-structured interviews, interviews; Other: Informal discussions | Data not extracted for eligible studies | No |
| Messer 2016 | USA, California | To expand life course theory and demonstrate how drug courts should be considered a facilitator of ˜turning points” for previous criminal offenders. | Foothill County Drug court:  A post-conviction court, where participants enter as a condition of formal probation or after a violation of probation from an existing criminal case. The underlying conviction need not be for a drug or drug-related offense, but if it is established the participant has a drug or alcohol dependency, then the individual can be referred to the drug court. The designed length of program is 24 months. There are four phases and the following tasks need completing: earn a general educational development certificate, attend drunken driving awareness courses, attend AIDS awareness courses, pay victim restitution, pay court fees, attend 12-step meetings, provide clean urine samples, attend individual or group drug treatment sessions, live in residential drug treatment, maintain employment or actively seek employment, and attend individual and/or group counselling sessions. | Justice-involved men; Justice-involved women | Not reported | Not reported | Interview study | Interview study | Data not extracted for eligible studies | No |
| Metzner 2017 | USA, North-eastern United States | To study how the drug courts distil meanings of responsibility:  The author aimed to make recommendations that address how drug court practitioners (within their role) struggle to meet the demands of competing ethical principles and priorities of justice and care. They aim to question assumptions about the therapeutic uses of urine drug tests and questions the assumptions made around the conceptualization of addiction. Instead of seeking to filter the good, recovering, and responsible addicts from the bad, drug court practitioners should work creatively and comprehensively to maximize success of all defendants. (Aim not explicitly stated). | Drug Court:  Drug treatment courts are specialized criminal courts that offer defendants, whose “non-violent” crimes are deemed to be related to problematic drug use, the chance to plead guilty and undergo intensive judicial monitoring of their drug treatment for a minimum of one year in advance of their sentencing. If they comply and succeed in treatment, their guilty pleas are withdrawn and their charges generally dismissed. If they are not able to comply, often after they are given several “chances”, they must generally serve a minimum one year sentence in state prison. | Justice-involved men; Justice-involved women; Judges/sheriffs; Probation officers or equivalent; Other: Assistant district attorney; statewide coordinator; treatment resource coordinator. | Not reported | Alcohol; Drugs. | Ethnography | Observation e.g. participant observation; Semi-structured interviews, interviews; Focus groups. | Data not extracted for eligible studies | No |
| Miller 2020 | USA, Nevada | Whereas the USA has numerous problem solving courts (e.g., drug courts, mental health courts), other countries do not have such courts, have altered versions of these courts, or have problem solving courts for other social issues. This qualitative research develops hypotheses regarding the reasons for such diversity. "Ultimately, this inquiry focused on the research question: What factors encouraged or discouraged the development of problem solving courts in various countries around the world? | Drug Court:  Addresses differences in drug courts internationally and therefore there are variety of descriptions of the intervention e.g. USA drug courts are specific to these offenders; "the prevailing belief is that the role of a judge and the legal system is to adjudicate consequences for wrongdoing, not coordinate services to help the wrongdoers", but in France, the Netherlands, Italy, and Spain Judges are hands-on in the traditional inquisitorial system and thus play a major role in providing services to the offender but not considered special problem solving courts. | Judges/sheriffs; Other: graduate law students or professors; attendees of a European criminology conference. | Not applicable | Drugs | Interview study | Observation e.g. participant observation; Semi-structured interviews, interviews. | Data not extracted for eligible studies | No |
| Moore 2014 | Canada, region not reported | We draw on a field study of three drug treatment courts to show that responsibilisation strategies create a paradox of bulimic exclusion and empowerment for individual subjects. By theorizing three different subjectivities emerging from our research sites (outcasts, performers and true believers), we show how subjects of intervention actively work to negotiate their own experiences of responsibilization (not explicitly stated - taken from abstract). | Drug Treatment Courts:  judicially supervised addiction treatment programmes for individuals facing custodial sentences for non-violent offences who also have a demonstrated addiction to opiates, crack/cocaine or methamphetamines. Those enrolled in the drug treatment court programme attend daily treatment sessions as well as twice weekly court appearances to ˜check in” with the supervising judge about treatment progress. | Justice-involved men; Justice-involved women; Other: Described as "individuals who have participated in the DTC programme." | Not reported | Drugs | Interview study | Semi-structured interviews, interviews | Data not extracted for eligible studies | No |
| Moore 2017 | USA, Florida | The goal of this study was to address this gap in the literature [lack of qualitative studies on drug courts] by learning more about the experiences of men and women within the Pinellas County Adult Drug Court treatment program. | Pinellas County Adult Drug Court treatment program: YouCan (Youthful Offenders Uplifted to Cope With Addictions to Narcotics):  The Pinellas County Adult Drug Court program is available to non-violent felony offenders (who are also not sex offenders), who are 18 years of age or older, and for whom substance abuse issues have been identified. The program lasts approximately 24 months and requires monthly appearances before a judge, substance abuse treatment, community supervision (24 months of probation; 18 months of probation for those participating in pretrial intervention), and twice-per-week random urine analyses for drug screening. | Justice-involved men; Justice-involved women (Specifically "Young adult offenders" (18-26 years). | Not reported | Alcohol; Drugs | Grounded Theory study | Semi-structured interviews, interviews | Grounded Theory | Yes |
| Morse 2014 [linked to Morse 2015] | USA, An upstate New York city | Explores healthcare-related needs and motivations of women participants in Drug Treatment Court within the wider social context of their lives. Goals were 1) to explore the needs and barriers for vital healthcare services among women Drug Treatment Court participants from stakeholder perspectives of the women themselves, providers, and court staff; and 2) to integrate Self-Determination Theory concepts of autonomy, relatedness, and competence across the levels of the Socio-Ecological Model. | Drug Treatment Court offers community court-mandated treatment programs in lieu of incarceration, significantly reducing relapse and recidivism rates. Drug Treatment Court incorporates intensive judicial supervision and community-based treatment, facilitating use of substance abuse resources. | Justice-involved women; Other: Staff were in 2 groups 1.Court staff. 2. providers from community agencies, one serving medical and social needs of HIV patients, and one serving survivors of intimate partner violence. | Black, African, African-Caribbean, or African American; White | Alcohol; Drugs | Focus Group Study | Focus Groups | Other: community-based participatory research. | Yes |
| Morse 2015 [linked to Morse 2014] | USA, a moderately large upstate city in the state of New York | The study purpose is to explore the systemic issues faced by women participants in drug treatment court from multiple perspectives to understand how these issues may relate to their health and wellness. | Drug Treatment Court:  Drug treatment court is designed to help a substance-abusing justice-involved individual link with care and stop abusing drugs. To achieve these goals, DTCs engage with community partners that provide services targeting the behaviour the legal system hopes to change with the use of “legal leverage” - a court process which can restrict freedom in order to improve adherence to a particular treatment plan as an alternative to long-term incarceration. | Justice-involved women; Other: Court staff, community providers | Black, African, African-Caribbean, or African American; White | Not reported | Focus Group study | Focus groups | Other: community-based participatory research | Yes |
| Murphy 2008 | USA, Philadelphia | To explore how a “medicalized” concept of addiction (as in, a disease that needs to be treated) is articulated and reinforced within a criminal justice setting (Chapter 4). | Philadelphia Drug Court:  Described in 4 Phases:  1. Remain drug-free for 30 days, attend treatment sessions, meet with case manager, have no convictions;  2. Remain drug-free for a further 3 months and complete as Phase 1;  3. As above for a further 30 days and pay all court costs/fines;  4. Another four months of proper attendance, no new convictions, and being drug-free for 120 days. Then follows a graduation ceremony. | Justice-involved men; Justice-involved women; Other: Assistant public defender; evaluator; case management supervisor; court coordinator;counsellors; clinical/program supervisors. | Not reported | Drugs | Interview study | Observation e.g. participant observation; Semi-structured interviews, interviews | Data not extracted for eligible studies | No |
| Murphy 2011 | USA, A large north-eastern city. | To explore how addiction is conceptualised in a drug court program in terms of how the philosophy and practice of addiction treatment is integrated into a criminal justice setting (differently stated in abstract & introduction). | Drug Court program:  Integration of alcohol and drug treatment services with case processing, the promotion of abstinence through frequent alcohol and drug testing, identifying eligible participants early, using a non-adversarial approach, ongoing judicial interaction with each participant, and close monitoring of the participant's progress in achieving set goals. | Social workers or equivalent; Other: Court staff (the court evaluator, public defender, and the program evaluator). | Not applicable | Alcohol; Drugs | Ethnography | Observation e.g. participant observation; Semi-structured interviews, interviews. | Other: Ethnographic research | Yes |
| Narag 2013 | USA, midwestern city | To understand the lived experiences of participants who underwent a Driving Under the Influence/ Driving While Impaired program as part of a process evaluation. | Driving Under the Influence or Driving While  Impaired (DUI/DWI) Court program:  Eligible participants: two or more drunk-driving offences but not been convicted of a violent offence. Participants volunteer for a 36-week program in exchange for a suspension of their prison sentence. Program elements include drug/alcohol monitoring, support groups, counselling, and extensive supervision. | Justice-involved men; Justice-involved women | Not reported | Alcohol | Interview study | Semi-structured interviews, interviews | Data not extracted for eligible studies | No |
| Nicholson 2015 | USA, County of Orange, California | Purpose of the study: "The current study sought to understand the lived experiences of participants taking part in Adult Drug Court in the Superior Court of California, County of Orange. It examined the essays written by each participant at each of the phases of treatment. The essays provided a unique vantage point into each participant’s experience while in the drug court program and gave rich information about what they found contributed to their success in the program. It is hoped that this information can help to better inform the justice system, alcohol and drug counsellors, and mental health clinicians as they work with substance abuse issues. While it will be useful  information for drug courts in general, it is hoped that the findings will inform mental health services regarding the treatment of substance abuse on a broader context." | The Orange County Drug Court:  The Orange County Drug Court program consists of nine rules, which all participants are required to adhere to (1) Do not use, or possess, any drugs or alcohol; (2) Attend all ordered treatment meetings; (3) Report to the Probation Office as directed; (4) Submit to drug testing: a participant must give a urine sample at each visit to HCA (Health Care Agency’s Division of Alcohol and Drug Abuse Services); (5) Be on time for all appointments including court appearances; (6) Participants are not to make threats toward other participants or staff, or behave in a violent or threatening manner; (7) Participants are to dress appropriately for court and treatment sessions; (8) While in court, remain seated and quiet at all times; (9) Police informant activity: the drug court is attempting to help participants remove themselves from drug culture and are not able to participate as a police informant.  Sanctions and treatment details are also provided here. | Justice-involved men; Justice-involved women. | Asian (not specified); Hispanic / Latinx; White. | Alcohol; Drugs | Grounded Theory study | Other: Archived sets of drug court participant essays (comprising up to 5 written essays for each participant). | Data not extracted for eligible studies | No |
| Nolan 2002 | USA, study covered a total of 21 states and the District of Columbia.. | This article examines the application of the disease model to drug and non-drug-related crimes in the context of drug treatment courts, and considers the significance of these developments as it concerns the meaning of criminal justice. | The drug treatment court offers the drug offender a therapeutic alternative to the traditional adjudication process. With the promise that successful completion will result in the dismissal of a charge or the expungement of an arrest, offenders are offered court-monitored-therapy instead of incarceration. | Judges/sheriffs; Other: District attorneys, public defenders, treatment counsellors, private attorneys, program coordinators, evaluators, and acupuncturists. | Not applicable | Alcohol; Drugs | Interview study | Observation e.g. participant observation; Semi-structured interviews, interviews. | Data not extracted for eligible studies | No |
| Osilla 2017 | USA, Los Angeles County | To evaluate the acceptability of cognitive behavioural treatment for alcohol use disorders adapted for driving under the influence programs. | Cognitive behavioural treatment for driving under the influence programs:  Most individuals arrested for driving under the influence are mandated to attend a driving under the influence alcohol education program to reinstate their driver's license and/or satisfy court sentencing. These programs consist of education classes and process groups that may be didactic in nature (e.g. lectures or films about the consequences of drinking and driving). The current study adapts an existing, evidence-based, manualized cognitive behavioural therapy treatment for alcohol use disorders. | Justice-involved men; Justice-involved women. | Black; African; African Caribbean; African American; Hispanic / Latinx; White; Mixed. | Alcohol | Not reported | Focus groups | Data not extracted for eligible studies | No |
| Pivovarova 2023 | USA, North-eastern US state | To identify facilitators and barriers from drug court staffs' perspectives in collaborating with Medication for Opioid Use Disorder (MOUD) providers in referring and retaining drug court participants in treatment. | Drug court program-substance use treatment:  Medication for opioid use disorder (MOUD), including three types of pharmacotherapies: methadone, buprenorphine, and naltrexone. | Judges/sheriffs; Lawyers; Social workers or equivalent; Probation officers or equivalent. | Not applicable | Drugs | Not reported | Semi-structured interviews, interviews | Data not extracted for eligible studies | No |
| Powell 2012 | UK, England (region not reported) | 1) to sample Drug Treatment and Testing Order staff’s opinion about the overall impact of the orders;  2) to sample other agency workers and court staff’s opinions about the overall impact of the orders (Study 3) | Drug treatment and testing orders:  Drug Treatment and Testing Orders (DTTOs) were aimed at dependent users of heroin or other opiates, cocaine and amphetamines who were convicted of acquisitive crimes (burglary, robbery, theft including shoplifting) as an alternative to custodial sentence. Assessment was on four main criteria: type and seriousness of the offence; seriousness of drug problem and susceptibility to treatment; motivation to change; and volume of drug related offending. DTTO involved drug testing, monthly reviews, and one to one and work sessions for a minimum of 15 hours/week. | Judges/sheriffs; Probation officers or equivalent; Other: Staff from NHS drug treatment services; Local Arrest Referral scheme; Community Care Assessors; magistrates; commissioners; local voluntary housing agency; case managers. | Not applicable | Drugs | Not reported | Semi-structured interviews, interviews | Other: template organising style and analysis (Crabtree & Miller, 1999). | Yes |
| Ramey 2023 | USA, Mid-Atlantic state. | To explore the experiences of team members to answer the following research questions: What is the purpose and value of drug court? How does drug court influence public safety, procedural justice, and therapeutic jurisprudence? | Drug Treatment Court:  Problem-solving courts (drug treatment court) follow 10 key concepts: integrated addiction treatment, nonadversarial approach, identification of participant eligibility, provision of a continuum of treatment services, frequent drug screenings, provision of rewards and sanctions, ongoing judicial involvement, monitoring and evaluation of the program, continuing education for drug court team, and community partnerships. | Judges/sheriffs; Lawyers; Probation officers or equivalent; Other: Drug court coordinator, prosecutor, peer recovery specialists, clinical supervisors, mitigation specialist, and treatment providers. | Not applicable | Alcohol; Drugs | Interview study | Semi-structured interviews, interviews | Data not extracted for eligible studies | No |
| Ricketts 2005 | UK, England, South Yorkshire | The current study sought to enhance understanding of the experience of the UK offenders participating in DTTO programmes through the development of a theoretical framework grounded in the reports of offenders. A qualitative approach was used to develop a detailed understanding of the following questions:  What are the reported experiences of offenders in engaging with the South Yorkshire DTTO programmes? What processes are common to successful engagement and how do they differ in an unsuccessful engagement? | Drug Treatment and Testing Orders (DTTOs):  DTTO is a community sentence under the 1998 UK Crime and Disorder Act intended for drug users who have a significant record of drug-related offending. It involves joint working between criminal justice, health and voluntary sector providers to deliver assessment, treatment and testing. DTTO usually requires a high level of involvement in treatment, with an initial attendance of 20 hours per week. Contact with the drug treatment service includes twice weekly supervised collection and testing of urine for drug use. Offenders are reviewed monthly by the sentencing court at the outset of the Order. | Justice-involved men; Justice-involved women. | Asian (not specified); White; Other: Black Asian. | Drugs | Grounded Theory study | Semi-structured interviews, interviews | Other: constant comparative | Yes |
| Robertson 2012 | USA, Mississippi | Research Questions: Is there a need to address HIV/STI risk-reduction within drug courts? What challenges might arise in attempting to deliver an HIV risk reduction intervention within the drug court setting? Finally, we wanted to know the following: What relationship might exist between length of participation in the drug court program and engaging in sexual risk behaviours associated with HIV/STI transmission? | Drug Courts:  Programs varied in length from 2 to 5 years and can append aftercare services or supervised probation upon program completion. All programs have multiple phases in which the frequency of court appearances, NA/AA meeting attendance, and drug testing progressively decrease with advancement to the next phase. All drug court participants are expected to complete substance abuse treatment during the first phase of the program and thereafter to be clean and sober, employed or performing community service unless physically disabled, and to obtain a General Educational Development (GED) if they have not graduated from high school. All drug court clients receive HIV risk assessments. | Justice-involved men; Justice-involved women; Other: Program coordinators | Black, African, African-Caribbean, or African American; White; Other: Other. | Alcohol; Drugs | Interview study | Semi-structured interviews, interviews; Other: Justice-involved participants completed survey relating to HIV/STD knowledge and attitudes (not open-ended). | Data not extracted for eligible studies | No |
| Salzman 2023 | USA, Sarasota, Florida | To examine the interaction of competing pressures as experienced by criminal-Justice-involved pregnant women and mothers and how this impacts motivation to desist from illicit drug use and crime. | First Step Addiction Recovery, Mother and Infants (FSMI) program:  Operating as a residential substance-abuse treatment facility, women reside on-site for up to twelve months. It combines group and individual counselling with parenting, nutrition and life management skills classes. Women participating in this program do not pay for the counselling or accommodation they receive during their stay. They also receive free pre-natal care through the Sarasota County Health Department. Women who attend can be court-ordered or self-referred. | Justice-involved women. | Hispanic / Latinx; White | Drugs | Qualitative | Semi-structured interviews, interviews | Thematic | Yes |
| Sarmiento 2019 | Australia, Victoria | Study aims to analyse the way one drug court (Victoria) conceptualizes the problem of alcohol and other drug “dependence” and seeks to respond to it through the administration of an onerous drug-testing regime. It focuses on the first, fourth, and fifth questions of Bacchi’s (2009) "What’s the Problem Represented to be?” (WPR) approach:  1. What is the “problem” represented to be in a specific policy? 2. What presuppositions (background knowledge) or assumptions (about the world) underlie the representation of this “problem”? 3. How has this representation of the “problem” come about? 4. What is left unproblematic in the “problem” representation? Where are the silences?  5. What effects are produced by this representation of the “problem”  (not explicitly stated). | Drug Court of Victoria:  There are 3 Phases of the drug court testing program. Specifically urine testing involves:  Phase 1: participants are required to undergo a urine test 3 times a week.  Phases 2 & 3: participants are required to undergo a urine test twice a week.  Drug classes tested are amphetamines, opiates, benzodiazepines, cocaine, cannabinoids, and alcohol. At the special request of the court, testing can confirm drugs such as methadone, suboxone, naltrexone, and buprenorphine. Other substances may be tested for whether, during the clinical assessment, the court deems the participant “dependent” on them or suspects they are using them while in the program. | Justice-involved men; Justice-involved women; Other: Members of drug court team. | Other: - Culturally and linguistically diverse (CALD) / Turkish born.  - Anglo-Australian.  - CALD / Australian born/Turkish background.  - CALD / Australian born / Greek background.  - CALD / Pacific Islander background  - CALD / Australian born / Spanish background.  - CALD / Indian born. | Drugs | Ethnography | Observation e.g. participant observation; Semi-structured interviews, interviews. | Thematic | Yes |
| Schiff 2010 | Canada, Regina, Saskatchewan | To explore the housing needs and preferences of five Aboriginal women involved with a drug treatment court. | Regina drug treatment court:  To provide offenders with an option to undertake extensive community-based treatment instead of being incarcerated. | Justice-involved women. | Aboriginal / First Nations; Mixed; Other: Métis (of mixed First Nations and French-Canadian ancestry). | Drugs | Interview study | Semi-structured interviews, interviews | Other: Deductive categorisation of data which were coded topically and axially. | Yes |
| Schrubbe 2020 | USA, region reported as “a rural town.” | The purpose of this study was to explore the overall performance of a drug court treatment program by answering the research question: What is the experience of participants who have successfully  graduated from a drug court treatment program? | Drug Court:  Drug courts consist of four elements: drug treatment, social support, regular reporting to the court, and regular urine screening. Drug relapses are expected and accepted as part of the recovery process, with a system of graduated sanctions and rewards delivered in response to compliance with the program (p33 "Elements of Drug Court”). NB: the Drug court used in this study is not explicitly described. | Justice-involved men; Justice-involved women. | Not reported | Alcohol; Drugs | Interview study | Semi-structured interviews, interviews. | Data not extracted for eligible studies | No |
| Scofield 2016 | USA, Hawaii | To identify cultural components that may affect motivation toward program advancement and graduation. | Drug Court Programs:  Most drug court programmes function in similar fashion with similar requirements, phases, incentives, and sanctions, and staffing components. The average time spent in the program ranges from 12 to 18 months. As is consistent with nationwide trends some participants may remain in program beyond 18 months because of program and treatment progress. There have been participants who have remained in the program for close to five years and have been encouraged to finish/graduate or the outcome would be probation revocation. | Justice-involved men; Justice-involved women. | Other: Mixed Hawaiian, Japanese, Native Hawaiian, Filipino | Alcohol; Drugs | Grounded Theory study | Semi-structured interviews, interviews | Data not extracted for eligible studies | No |
| Shannon 2017 | USA, Kentucky, Jefferson county, Hardin County | To examine implementation and performance indicator data for two Kentucky VTCs to better understand the unique accomplishments and challenges associated with program development, as well as key features of VTC implementation. | Kentucky veteran treatment court:  Offer tailored rehabilitation for non-violent veterans charged with drug or drug-related crimes. It consists of three phases and an after care component, which can be completed in a minimum of 18 months. | Veterans who use drugs &/or alcohol & have committed a crime; Judges/sheriffs; Lawyers; Social workers or equivalent; Probation officers or equivalent; Other: Administration. | Not reported | Drugs | Not reported | Semi-structured interviews, interviews | Data not extracted for eligible studies | No |
| Sousa 2021 | USA, Western State | This study aims to investigate the ways in which drug treatment courts go about the task of modifying participants’ behaviours with the twin goals of reducing recidivism and treating criminal offenders with severe drug addictions. | Western County Drug Court:  The Western County Drug Court has five distinct phases:  1 Orientation (30 days minimum) including drug/alcohol assessment, twice weekly meetings with probation officer, group activities, attend biweekly court review proceedings.  2 (60 days minimum) - As phase 1 but reduced meetings with probation officer, urine testing, address any health issues, secure employment. 60 days sobriety mandatory.  3 (90 days minimum) - As above, maintaining sobriety and compliance.  4. (90 days minimum) - If participant has succeeded with above phases, they may be placed on monthly checks.  5 Graduation & maintenance (180 days minimum) - complete treatment, mentor clients in Phases 1&2, voluntary community activities. | Judges/sheriffs; Other: Public defender, graduates of the drug court program, directors of a therapeutic community, a manager of a sober living facility, group treatment support facilitator. | Not applicable | Alcohol; Drugs | Case study | Observation e.g. participant observation; Semi-structured interviews, interviews | Data not extracted for eligible studies | No |
| Stahl 2018 | USA, Atlantic County | To understand the behaviors, interventions, and other deterrence factors that Atlantic county drug court probation officers perceived to impact drug court participants’ success or failure in the drug court program. | The Atlantic County drug court:  Drug court program consist in four stages. Phases 1A and 1B require a minimum engagement of three months, while phases 2 through 4 require six months each before advancing to the next phase or graduation. Advancement depends on meeting criteria such as staying drug and alcohol-free, avoiding new legal issues, adhering to treatment, maintaining employment or school activities, attending self-help meetings, and complying with legal and probation requirements. | Probation officers or equivalent. | Not applicable | Alcohol; Drugs | Case study | Semi-structured interviews, interviews | Data not extracted for eligible studies | No |
| Staton 2001 | USA, Kentucky | To gain a better understanding of employment needs of Drug Court clients in order to target and refine the enhanced employment intervention. To develop and evaluate an enhanced Drug Court employment intervention with the goals of improving drug treatment retention and reducing recidivism. | Enhanced Drug Court employment intervention:  An enhanced employment intervention, which is designed to coincide with the three phases of Drug Court treatment. Treatment includes outpatient groups, Alcoholics Anonymous/Narcotics Anonymous groups, and individual sessions focusing on addiction as well as criminal thinking and takes the average client 18 months to complete. Treatment will be complemented by job skill training, social skill training, strengths based case management, and motivational interviewing. | Justice-involved men; Justice-involved women | Not reported | Not reported | Focus Group study | Focus groups | Data not extracted for eligible studies | No |
| Thom 2018 | Australia (region not reported) | To explore how the court teams’ work for the court differs to their practice as usual; how they define and understand their use of therapeutic principles and how the requirements of the courts shape their existing professional understandings of therapeutic discourse. | Alcohol and other drug treatment court (AODT):  Alternative, non-adversarial approach for responding to criminal offending where it is driven, by a dependency on alcohol or other drugs. Three-phased drug court program. Phase 1: intensive treatment and rehabilitation, random drug testing and frequent appearances in court for judicial monitoring. Phases 2 and 3 continue to include treatment and rehabilitation, inclusive of trauma counselling and behavioural modification programs, and drug testing, but with increasing intervals between court appearances focus on longer-term solutions. Phase 3 includes preparations for transitioning into living in the community in a relatively stable state of recovery. | Judges/sheriffs; Social workers or equivalent; Other: Cultural advisor, case manager, court coordinator, counsel, police prosecutor. | Not applicable | Alcohol; Drugs | Ethnography | Observation e.g. participant observation; Semi-structured interviews, interviews; Focus groups; Other: AODT Court handbooks and United-States based best practice documents | Data not extracted for eligible studies | No |
| Tiger 2009 | USA, New York | Research Questions:  (1) How are the seemingly contradictory approaches to dug use - therapeutic and punitive - merged in the concept of drug courts?  (2) What knowledge do drug court advocates draw upon to reconstruct the problem of addiction and articulate a role for the courts, in solving social problems?  (3) What theories about addiction, treatment, and the problem-solving role of the criminal justice system do drug court advocates construct to justify and expand their institution’s scope? | Drug Court:  The individual is arrested for drug-related offenses and mandated to drug treatment instead of prison. Looks at the specific aspect of drug court treatment, the effect and role of coercion in treating offenders with addiction from the perspective of drug court advocates (a broad description of people working within drug courts to protect identity). | Other: drug court advocates, proponents. | Not applicable | Alcohol; Drugs | Grounded Theory study | Semi-structured interviews, interviews | Data not extracted for eligible studies | No |
| Tiger 2011 | USA, (region not reported) | (1) To discuss how punitive, therapeutic, and medical approaches are merged to enlarge the scope of activities used to manage individuals deemed both criminal and sick and (2) to examine the ways the logic of coerced treatment reflects both the rehabilitative and repressive approaches that have characterized U.S. criminal justice practice and policy. | Drug Court:  Drug courts leverage the coercive power of the criminal justice system to achieve abstinence and alter criminal behaviour. The structure of drug courts differs but they share three main common features: use of “legal and external pressure” through the power of the judge to mandate people to drug treatment, heavy monitoring of progress, use of sanctions to punish noncompliance and incentives to reward progress. | Other: Drug court representatives | Not applicable | Not reported | Not reported | Semi-structured interviews, interviews; Other: Documents generated by the advocacy organizations, governmental agencies, and research centers concerned with drug courts and their expansion. These documents include professional newsletters, practitioner fact sheets, The National Drug Court Institute Review journal, training materials, and evaluation research. | Data not extracted for eligible studies | No |
| Ward 2016 | USA, (region not reported) | To explore the culture, roles, and operating rules of a drug court’s workgroup using seminal concepts derived from studies of traditional courtroom workgroups; the focal concerns of the members of that workgroup; and the way in which this drug court implements public policy regarding the re-entry and re-integration of offenders under supervised release for drug-and-drug-related crimes. | Drug Court program:  The program follows the key components of drug courts. Participants follow a program of supervision and drug testing for a period of 12 to 18 months. In order to successfully complete the program, participants must achieve the stated program goals of being sober, law-abiding, and employed. | Judges/sheriffs; Lawyers; Social workers or equivalent; Probation officers or equivalent. | Not applicable | Alcohol; Drugs | Phenomenology | Observation e.g. participant observation; Semi-structured interviews, interviews | Data not extracted for eligible studies | No |
| Waterman 2018 | USA, Texas | To utilise a narrative, music-informed approach to document the stories of women in early recovery as they describe their (a) past experiences in active addiction, (b) transition into recovery, (c) current recovery life, and (d) hopes for the future. The study will also explore the significance of drug court in participants’ recovery experiences. | A Musical Chronology and the Emerging Life Song:  A counselling intervention that offers music as a therapeutic tool that clients can use to gain clarity around a significant life experience. Four phases: (1) Clients make a list of meaningful songs related to the phase of life they are interested in processing, and arrange those songs in chronological order; (2) Clients listen to the songs in the presence of a counsellor, and describe any thoughts or emotions they experience; (3) Clients identify which songs specifically describe their current emotional state, which provides the counsellor with insight into their present frame of mind; (4) Clients then provide a song or songs to the counsellor that represents their life aspirations. The fourth musical selection expresses their hopes for the future, and serves as the client’s goals for counselling. | Justice-involved women; | Not reported | Alcohol; Drugs | Music informed narrative inquiry | Semi-structured interviews, interviews; Other: Musical chronology | Data not extracted for eligible studies | No |
| Wolfer 2008 | USA, a medium sized city in the eastern half of Pennsylvania | To examine how drug courts work from the clients' perspective and looks for any themes that would resonate with criminological or sociological theory. | Drug Court:  Program designed to last 12-18 months, during which defendants progress through four phases in which individual and group treatment sessions are provided, in a fifth phase the original charges are expunged if the person is not re-arrested for a drug related offense one year after graduation. | Justice-involved men; Justice-involved women; | White; Other: Racial minority (two of seventy) | Alcohol; Drugs | Not reported | Semi-structured interviews, interviews | Data not extracted for eligible studies | No |
| Yang 2019 | USA, Midwestern metropolitan area. | The current study used a mixed-method design to examine the influence of client- and counsellor-level factors on 90-day treatment retention, satisfaction, and progress for justice-involved individuals referred to medication-assisted treatment. | Community based medication assisted treatment program:  Provides options for buprenorphine and depot naltrexone. In addition to the medication, clients visited the treatment facility three to five times per week for a combination of group and individual counselling. | Justice-involved men; Other: Substance use counsellors. | Black, African, African-Caribbean, or African American; White; Other | Drugs | Interview study | Semi-structured interviews, interviews | Data not extracted for eligible studies | No |
| Zaller 2016 | USA, Arkansas: Mississippi Delta Region and Pulaski Country | To explore the relationships between ongoing involvement in the criminal justice system and continued drug use in a population of urban and rural African American cocaine users in a Southern State. | Criminal justice system (incarceration, drug treatment):  No detailed description provided. | Justice-involved men; Justice-involved women; | Black, African, African-Caribbean, or African American | Drugs | Part of a sequential mixed-methods study. | Semi-structured interviews, interviews | Data not extracted for eligible studies | No |

(Abdul-Malik, 2022; Ahmed et al., 2022; Baker, 2009; Bates, 2009; Belenko, Fabrikant, & Wolff, 2011; Bevli, 2018; Brook, 2011; Burns & Peyrot, 2003; Butler, 2013; Crosson, 2016; Csete & Catania, 2013; Datchi & Ancis, 2017; Devall, 2008; Dickson-Gomez et al., 2022; Eley, Malloch, McIvor, Yates, & Brown, 2002; Engstrom, 2023; Evans, Anglin, Urada, & Yang, 2011; Farringer & Manchak, 2023; Fischer, Geiger, & Hughes, 2007; Fisher, 2009; Francis & Abel, 2014; Fulkerson, Keena, & O'Brien, 2012; Gallagher, 2013; Gallagher et al., 2023; Gallagher & et, 2021; Gallagher, Marlowe, & Minasian, 2019; Gallagher & Nordberg, 2017; Gallagher, Nordberg, Deranek, & Minasian, 2019; Gallagher, Nordberg, & Dibley, 2019; Gallagher, Nordberg, & Gallagher, 2017, 2018; Gallagher, Nordberg, & Kennard, 2015; Gallagher, Nordberg, & Lefebvre, 2017; Gallagher & Wahler, 2018; Gallagher, Wahler, Minasian, & Edwards, 2019; Garcia, Kenyon, Brolan, Coughlin, & Guedes, 2019; Goldberg, Chin, Alio, Williams, & Morse, 2019; Hamilton, 2019; Hardy, Teruya, Longshore, & Hser, 2005; Harrell, Cavanagh, & Roman, 1998; Hennessy, Krasnoff, & Best, 2023; Horowitz & Gowan, 2023; Horrocks, Barker, Kelly, & Robinson, 2004; Kahn, Brimmer, Berdine, Lawson, & Homish, 2023; Kennedy-Hendricks, Bandara, Merritt, Barry, & Saloner, 2021; Kerr et al., 2011; Kouimtsidis, Reynolds, & Asamoah, 2007; Larsen, 2014; Lutze & van Wormer, 2014; Lyons, 2014; Mackinem & Higgins, 2007, 2010; Maddox, 2023; McIvor, 2009; McIvor et al., 2006; McPherson & Sauder, 2013; Messer, Patten, & Candela, 2016; Metzner, 2017; Miller, 2020; D. Moore & Hirai, 2014; K. A. Moore, Barongi, & Rigg, 2017; Morse et al., 2014; Morse, Silverstein, Thomas, Bedel, & Cerulli, 2015; Jennifer Murphy, 2008; J. Murphy, 2011; Narag, Maxwell, & Lee, 2013; Nicholson, 2015; Nolan, 2002; Osilla, Kulesza, & Miranda, 2017; Pivovarova et al., 2023; Powell, 2012; Ramey, Volk, Milacci, & Kelley, 2023; Ricketts, Bliss, Murphy, & Brooker, 2005; Robertson, St, & McCluskey, 2012; Salzman, 2023; Sarmiento, Seear, & Fraser, 2019; Schiff & Waegemakers Schiff, 2010; Schrubbe, 2020; Scofield, 2016; Shannon et al., 2017; Sousa, 2021; Stahl, 2018; Staton et al., 2001; Thom & Black, 2018; Tiger, 2009, 2011; Ward, 2016; Waterman, 2018; Wolfer & Roberts, 2008; Yang, Gray, Joe, Flynn, & Knight, 2019; Zaller, Cheney, Curran, Booth, & Borders, 2016)

## References

Abdul-Malik, H. (2022). *Experiences of African Americans regarding treatment plans offered by drug courts: A transcendental phenomenological study.* University of Phoenix, Phoenix, Arizona.

Ahmed, F. Z., Andraka-Christou, B., Clark, M. H., Totaram, R., Atkins, D. N., & Del Pozo, B. (2022). Barriers to medications for opioid use disorder in the court system: provider availability, provider "trustworthiness," and cost. *Health & Justice, 10*(1), 24. doi:/10.1186/s40352-022-00188-4

Baker, K. M. (2009). *"I'm just not sure what we are going to do with her": Ambivalence and conflict in drug court decision-making.* The University of Texas at Austin, Austin, Texas.

Bates, T. J. (2009). *Drug court: Breaking the black magic spell of drug addiction for women: A qualitative study.* (Doctor of Philosophy). The University of Utah, Dissertation Abstracts International Section A: Humanities and Social Sciences.

Belenko, S., Fabrikant, N., & Wolff, N. (2011). The long road to treatment: Models of screening and admission into drug courts. *Criminal Justice and Behavior, 38*(12), 1222-1243. doi:/10.1177/0093854811424690

Bevli, S. (2018). *Effectiveness of the substance abuse and crime prevention act: the experiences of Hispanic residents.* (Doctor of Psychology). University of the Rockies, Dissertation Abstracts International: Section B: The Sciences and Engineering.

Brook, K. K. (2011). *Drug courts and the treatment of addiction: Therapeutic jurisprudence and neoliberal governance.* (Doctor of Philosophy). New York University, New York.

Burns, S. L., & Peyrot, M. (2003). Tough love: nurturing and coercing responsibility and recovery in California drug courts. *Social Problems, 50*(3), 416-438. doi:/10.1525/sp.2003.50.3.416

Butler, S. (2013). The symbolic politics of the Dublin drug court: The complexities of policy transfer. *Drugs: Education, Prevention & Policy, 20*(1), 5-14. doi:10.3109/09687637.2012.740524

Crosson, F. (2016). *The evolving relationship between drug court judge and offender.* (Doctor of Philosophy). Capella University,

Csete, J., & Catania, H. (2013). Methadone treatment providers' views of drug court policy and practice: a case study of New York State. *Harm Reduction Journal, 10*( 35).

Datchi, C. C., & Ancis, J. R. (2017). Women and adult drug treatment courts: Surveillance, social conformity, and the exercise of agency. In J. R. Ancis (Ed.), *Gender, psychology, and justice: The mental health of women and girls in the legal system* (pp. 101-126). New York, NY: New York University Press; US.

Devall, K. E. (2008). *The theory and practice of drug courts: Wolves in sheep clothing.* (Doctor of Philosophy). Western Michigan University Kalamazoo, Michigan

Dickson-Gomez, J., Spector, A., Krechel, S., Li, J., Montaque, H. D. G., Ohlrich, J., . . . Weeks, M. (2022). Barriers to drug treatment in police diversion programs and drug courts: A qualitative analysis. *Am J Orthopsychiatry, 92*(6), 692-701. doi:10.1037/ort0000643

10.1037/ort0000643. Epub 2022 Oct 13.

Eley, S., Malloch, M., McIvor, G., Yates, R., & Brown, A. (2002). *The Glasgow drug court in action: the first six months*. Retrieved from Scotland:

Engstrom, R. (2023). *An analysis of Binder County DWI Court: A case study.* University of St. Thomas, Minnesota, Minnesota.

Evans, E., Anglin, M. D., Urada, D., & Yang, J. (2011). Promising practices for delivery of court-supervised substance abuse treatment: Perspectives from six high-performing California counties operating Proposition 36. *Evaluation and Program Planning, 34*(2), 134.

Farringer, A. J., & Manchak, S. M. (2023). Communication and collaboration in a drug court team. *Psychological services, 20*, 929-940. doi:/10.1037/ser0000735

Fischer, M., Geiger, B., & Hughes, M. E. (2007). Female recidivists speak about their experience in drug court while engaging in appreciative inquiry. *International Journal of Offender Therapy & Comparative Criminology, 51*(6), 703-722. doi:10.1177/0306624X07299304

10.1177/0306624X07299304. Epub 2007 Jul 5.

Fisher, R. D., Jr. (2009). *The phenomenological essences of the experiences of drug court attorneys: An examination of moral development.* (Doctor of Philosophy ). Capella University

Francis, T. R., & Abel, E. M. (2014). Redefining success: A qualitative investigation of therapeutic outcomes for noncompleting drug court clients. *Journal of Social Service Research, 40*(3), 325-338. doi:10.1080/01488376.2013.875094

Fulkerson, A., Keena, L. D., & O'Brien, E. (2012). Understanding success and nonsuccess in the drug court. *International Journal of Offender Therapy & Comparative Criminology, 57*(10), 1297-1316. doi:10.1177/0306624X12447774

10.1177/0306624X12447774. Epub 2012 May 28.

Gallagher, J. R. (2013). African American participants’ views on racial disparities in drug court outcomes. *Journal of Social Work Practice in the Addictions, 13*(2), 143-162. doi:10.1080/1533256X.2013.784689

Gallagher, J. R., Estreet, A., Nordberg, A., Zongrone, C., Minasian, R. M., & Szymanowski, S. (2023). The interplay between women, opioid use disorder, medication-assisted treatment (MAT), and drug court: A qualitative study. *Journal of Human Behavior in the Social Environment, 33*(5), 647-662. doi:/10.1080/10911359.2022.2077500

Gallagher, J. R., & et, a. (2021). A focus group analysis with a drug court team: opioid use disorders and the role of Medication-Assisted Treatment (MAT) in programming. *Journal of Social Work Practice in the Addictions, 21*(2), 139-148. doi:10.1080/1533256X.2021.1912964

Gallagher, J. R., Marlowe, D., & Minasian, R. (2019). Participant perspectives on medication-assisted treatment for opioid use disorders in drug court. *Journal for Advancing Justice, 2*, 39-54.

Gallagher, J. R., & Nordberg, A. (2017). A phenomenological and grounded theory study of women's experiences in drug court: Informing practice through a gendered lens. *Women & Criminal Justice, 27*(5), 340.

Gallagher, J. R., Nordberg, A., Deranek, M. S., & Minasian, R. M. (2019). Drug court through the lenses of African American women: improving graduation rates with gender-responsive interventions. *Women & Criminal Justice, 29*(6), 337.

Gallagher, J. R., Nordberg, A., & Dibley, A. R. (2019). Improving graduation rates for African Americans in drug court: Importance of human relationships and barriers to gaining and sustaining employment. *J Ethn Subst Abuse, 18*(3), 387-401. doi:10.1080/15332640.2017.1381661

10.1080/15332640.2017.1381661. Epub 2017 Nov 16.

Gallagher, J. R., Nordberg, A., & Gallagher, J. M. (2017). A qualitative investigation into military veterans' experiences in a problem-solving court: Factors that impact graduation rates. *Social Work in Mental Health, 15*(5), 487-499. doi:/10.1080/15332985.2016.1237925

Gallagher, J. R., Nordberg, A., & Gallagher, J. M. (2018). Participants' views on the strengths and limitations of drug court: Recommendations to enhance assessment and treatment of mental illnesses. *Social Work in Mental Health, 16*(4), 436-450. doi:10.1080/15332985.2017.1419536

Gallagher, J. R., Nordberg, A., & Kennard, T. (2015). A qualitative study assessing the effectiveness of the key components of a drug court. *Alcoholism Treatment Quarterly, 33*(1), 64-81. doi:/10.1080/07347324.2015.982453

Gallagher, J. R., Nordberg, A., & Lefebvre, E. (2017). Improving graduation rates in drug court: A qualitative study of participants' lived experiences. *Criminology & Criminal Justice: An International Journal, 17*(4), 468-484. doi:/10.1177/1748895816682578

Gallagher, J. R., & Wahler, E. A. (2018). Racial Disparities in Drug Court Graduation Rates: The Role of Recovery Support Groups and Environments. *Journal of Social Work Practice in the Addictions, 18*, 113-127.

Gallagher, J. R., Wahler, E. A., Minasian, R. M., & Edwards, A. (2019). Treating opioid use disorders in drug court: participants’ views on using medication-assisted treatments (MATs) to support recovery. *International Criminal Justice Review, 29*(3), 249-261. doi:10.1177/1057567719846227

Garcia, R. A., Kenyon, K. H., Brolan, C. E., Coughlin, J., & Guedes, D. D. (2019). Court as a health intervention to advance Canada's achievement of the sustainable development goals : a multi-pronged analysis of Vancouver's Downtown Community Court. *Global Health, 15*(1), 80. doi:10.1186/s12992-019-0511-9

10.1186/s12992-019-0511-9.

Goldberg, Z. E., Chin, N. P., Alio, A., Williams, G., & Morse, D. S. (2019). A qualitative analysis of family dynamics and motivation in sessions with 15 women in drug treatment court. *Substance Abuse, 4*(13).

Hamilton, L. (2019). *Health-related quality of life among community-based offenders: How 'well-being' affects substance abuse treatment engagement.* (Doctor of Philosophy). Temple University, Dissertation Abstracts International Section A: Humanities and Social Sciences.

Hardy, M., Teruya, C., Longshore, D., & Hser, Y.-I. (2005). Initial implementation of California's Substance Abuse and Crime Prevention Act: Findings from focus groups in ten counties. *Evaluation and Program Planning, 28*(2), 221-232. doi:/10.1016/j.evalprogplan.2004.03.001

Harrell, A., Cavanagh, S., & Roman, J. (1998). *Findings from the evaluation of the D.C. Superior Court drug intervention program*. Retrieved from US:

Hennessy, E. A., Krasnoff, P., & Best, D. (2023). Implementing a recovery capital model into therapeutic courts: Case study and lessons learned. *International Journal of Offender Therapy and Comparative Criminology*, Online ahead of print. doi:/10.1177/0306624X231198810

Horowitz, V., & Gowan, T. (2023). Feminized need and racialized danger: Punitive therapeutics and historical addict tropes in a Midwestern drug court. *Theoretical Criminology, 27*(1), 23-47. doi:/10.1177/13624806211060867

Horrocks, C., Barker, V., Kelly, N., & Robinson, D. (2004). Coercive treatment for drug misuse: A dialogical juncture. *Journal of Community & Applied Social Psychology, 14*(5), 345-355. doi:/10.1002/casp.797

Kahn, L. S., Brimmer, M. J., Berdine, D. E., Lawson, S. C., & Homish, D. L. (2023). Impacts of COVID-19 on drug treatment court operations: Lessons learned through normalization process theory. *Victims & Offenders, 18*, 1474–1497.

Kennedy-Hendricks, A., Bandara, S., Merritt, S., Barry, C. L., & Saloner, B. (2021). Structural and organizational factors shaping access to medication treatment for opioid use disorder in community supervision. *Drug Alcohol Depend, 226*, 108881. doi:10.1016/j.drugalcdep.2021.108881

10.1016/j.drugalcdep.2021.108881. Epub 2021 Jun 26.

Kerr, J., Tompkins, C., Tomaszewski, W., Dickens, S., Grimshaw, R., Wright, N., & Barnard, M. (2011). *The dedicated drug courts pilot evaluation process study*. Retrieved from Ministry of Justice, UK: [www.justice.gov.uk/publications/research.htm](https://stir-my.sharepoint.com/personal/eff2_stir_ac_uk/Documents/NESSIE%20NIHR%20Evidence%20Synthesis%20Group%20Todhunter%202022/Treatment%20order%20review%202023/TO%20Qual%20Review%20Article/Anonymised%20additional%20files%20for%20submisison/www.justice.gov.uk/publications/research.htm)

Kouimtsidis, C., Reynolds, M., & Asamoah, V. (2007). Treatment or prison: service user and staff experiences of drug treatment and testing orders. *Psychiatric Bulletin, 31*(12), 463-466. doi:10.1192/pb.bp.107.014548

Larsen, J. L. (2014). *Trauma and the justice-involved veteran.* (Doctor of Philosophy). University of California, Santa Barbara.

Lutze, F. E., & van Wormer, J. (2014). The reality of practicing the ten key components in adult drug court. *Journal of Offender Rehabilitation, 53*(5), 351-383. doi:/10.1080/10509674.2014.922155

Lyons, T. (2014). Simultaneously treatable and punishable: Implications of the production of addicted subjects in a drug treatment court. *Addiction Research & Theory, 22*(4), 293.

Mackinem, M. B., & Higgins, P. (2007). Tell me about the test: The construction of truth and lies in drug court. *Journal of Contemporary Ethnography, 36*(3), 223-251. doi:/10.1177/0891241606287417

Mackinem, M. B., & Higgins, P. (2010). Losing hope: The production of failure in drug court. In M. Peyrot & S. Lee Burns (Eds.), *New approaches to social problems treatment* (Vol. 17, pp. 179-204): Emerald Group Publishing Limited.

Maddox, M. E. (2023). *The effectiveness of drug treatment court: Participants' recommendations for improvement of the drug treatment court diversion program.* (Doctor in Psychology). William James College, Dissertation Abstracts International: Section B: The Sciences and Engineering.

McIvor, G. (2009). Therapeutic jurisprudence and procedural justice in Scottish Drug Courts. *Criminology & Criminal Justice, 9*(1), 29–49. doi:10.1177/1748895808099179

McIvor, G., Barnsdale, L., Eley, S., Malloch, M., Yates, R., & Brown, A. (2006). *The operation and effectiveness of the Scottish drug court pilots*. Retrieved from Scotland:

McPherson, C. M., & Sauder, M. (2013). Logics in action: Managing institutional complexity in a drug court. *Administrative Science Quarterly, 58*(2), 165-196. doi:10.1177/0001839213486447

Messer, S., Patten, R., & Candela, K. (2016). Drug courts and the facilitation of turning points: An expansion of life course theory. *Contemporary Drug Problems, 43*(1), 6-24. doi:10.1177/0091450916632545

Metzner, E. M. (2017). *Care beyond justice: The conflicted ethics of drug treatment courts.* University of Illinois at Urbana-Champaign, Urbana, Illinois.

Miller, M. K. (2020). A qualitative analysis and eleven-factor typology of hypothesized factors encouraging or discouraging the development of problem solving courts in various countries. *Journal of Experimental Criminology, 16*(1), 100.

Moore, D., & Hirai, H. (2014). Outcasts, performers and true believers: Responsibilized subjects of criminal justice. *Theoretical Criminology, 18*(1), 5-19. doi:/10.1177/1362480613519287

Moore, K. A., Barongi, M. M., & Rigg, K. K. (2017). The Experiences of Young Adult Offenders Who Completed a Drug Court Treatment Program. *Qual Health Res, 27*(5), 750-758. doi:10.1177/1049732316645782

10.1177/1049732316645782. Epub 2016 Jul 10.

Morse, D. S., Cerulli, C., Bedell, P., Wilson, J. L., Thomas, K., Mittal, M., . . . Chin, N. (2014). Meeting health and psychological needs of women in drug treatment court. *J Subst Abuse Treat, 46*(2), 150-157. doi:10.1016/j.jsat.2013.08.017

10.1016/j.jsat.2013.08.017. Epub 2013 Sep 24.

Morse, D. S., Silverstein, J., Thomas, K., Bedel, P., & Cerulli, C. (2015). Finding the loopholes: a cross-sectional qualitative study of systemic barriers to treatment access for women drug court participants. *Health & Justice, 3*, 12. doi:10.1186/s40352-015-0026-2

10.1186/s40352-015-0026-2. Epub 2015 Jun 17.

Murphy, J. (2008). *Therapy and punishment: Negotiating authority in the management of drug addiction.* (Doctor of Philosophy ). Temple University, Philadelphia, Pennsylvania

Murphy, J. (2011). Drug court as both a legal and medical authority. *Deviant Behavior, 32*(3), 257-291.

Narag, R. E., Maxwell, S. R., & Lee, B. (2013). A phenomenological approach to assessing a DUI/DWI program. *International Journal of Offender Therapy & Comparative Criminology, 57*(2), 229-250. doi:/10.1177/0306624X11431685

Nicholson, S. R. (2015). *Themes from the essays of participants who completed the Orange County Drug Court Program: A qualitative study.* (Doctor of Psychology). Alliant International University, San Diego San Diego.

Nolan, J. L., Jr. (2002). Drug treatment courts and the disease paradigm. *Substance Use & Misuse, 37*(12-13), 1723-1750.

Osilla, K. C., Kulesza, M., & Miranda, J. (2017). Bringing alcohol treatment to driving under the influence programs: Perceptions from first-time offenders. *Alcoholism Treatment Quarterly, 35*(2), 113-129. doi:/10.1080/07347324.2017.1288484

Pivovarova, E., Taxman, F. S., Boland, A. K., Smelson, D. A., Lemon, S. C., & Friedmann, P. D. (2023). Facilitators and barriers to collaboration between drug courts and community-based medication for opioid use disorder providers. *Journal of Substance Use and Addiction Treatment, 147*, 208950. doi:/10.1016/j.josat.2022.208950

Powell, C. L. (2012). *Coerced drug treatment in England and Wales: An evaluation of Drug Treatment and Testing Orders in one locality.* (PhD Psychology). University of Leicester, UK.

Ramey, J. S., Volk, F., Milacci, F., & Kelley, B. (2023). "If not you, then who?": a qualitative case study of a drug treatment court. *Journal of Social Work Practice in the Addictions* 1–17.

Ricketts, T., Bliss, P., Murphy, K., & Brooker, C. (2005). Engagement with drug treatment and testing orders: A qualitative study. *Addiction Research & Theory, 13*(1), 65-78. doi:10.1080/16066350512331328168

Robertson, A. A., St, & McCluskey, D. (2012). HIV/STI risk behavior of drug court participants. *Journal of Offender Rehabilitation, 51*(7), 453-473. doi:/10.1080/10509674.2012.702715

Salzman, H. J. (2023). *Motherhood and substance use: An examination of societal pressures in the motivation to complete court-ordered drug treatment and to desist from future criminal activity and drug use.* (Doctor of Philosophy). University of Manchester, Dissertation Abstracts International: Section B: The Sciences and Engineering, UK.

Sarmiento, E., Seear, K., & Fraser, S. (2019). Enacting alcohol and other drug (Testing)-related harms in an Australian drug court. *Contemporary Drug Problems, 46*(3), 282-230.

Schiff, R., & Waegemakers Schiff, J. (2010). Housing needs and preferences of relatively homeless Aboriginal women with addiction. *Social Development Issues, 32*(3), 65-76.

Schrubbe, M. (2020). *The experience of drug court participants: A generic qualitative inquiry.* Capella University

Scofield, M. S. (2016). *Culturally expanded perspectives: A grounded theoretical study of a drug court program.* (Doctor of Philosophy). Capella University

Shannon, L. M., Birdwhistell, S., Hulbig, S. K., Jones, A. J., Newell, J., & Payne, C. (2017). Examining implementation and preliminary performance indicators of veterans treatment courts: The Kentucky experience. *Evaluation & Program Planning, 63*, 54-66. doi:/10.1016/j.evalprogplan.2017.03.003

Sousa, M. D. (2021). *Therapeutic discipline: The governance of addicted criminal offenders in adult treatment court.* University of Colorado Boulder, Colorado

Stahl, J. H. (2018). *Probation officers' perceptions of factors that increase and decrease drug court participants' success: A qualitative exploratory case study.* University of the Rockies Denver, Colorado.

Staton, M., Mateyoke, A., Leukefeld, C., Cole, J., Hopper, H., Logan, T., & Minton, L. (2001). Employment issues among drug court participants. *Journal of Offender Rehabilitation, 33*(4), 73-85. doi:/10.1300/J076v33n04_05

Thom, K., & Black, S. (2018). Nga Whenu Raranga/Weaving strands in the alcohol and other drug treatment court of Aotearoa/New Zealand. *Journal of Law & Medicine, 25*(3), 727-740.

Tiger, R. (2009). *Drug courts and coerced treatment: The social construction of "enlightened coercion".* Erasmus University Rotterdam, Rotterdam.

Tiger, R. (2011). Drug courts and the logic of coerced treatment. *Sociological Forum, 26*(1), 169-182. doi:/10.1111/j.1573-7861.2010.01229.x

Ward, K. (2016). *Exploring the culture and concerns of the problem-solving court's workgroup in a federal, re-entry drug court and its impact on public policy: A phenomenological study.* Northeastern University, Boston, Massachusetts

Waterman, S. P. (2018). *Exploring the stories of women in early recovery participating in a drug court program: A narrative study using a musical chronology and the emerging life song.* (Doctor of Philosophy). The University of Texas at San Antonio, San Antonio.

Wolfer, L., & Roberts, J. C. (2008). A theoretical exploration of a drug court program based on client experiences. *Contemporary Drug Problems: An Interdisciplinary Quarterly, 35*(2-3), 481-507. doi:/10.1177/009145090803500213

Yang, Y., Gray, J., Joe, G. W., Flynn, P. M., & Knight, K. (2019). Treatment retention satisfaction, and therapeutic progress for justice-involved individuals referred to community-based medication-assisted treatment. *Substance Use & Misuse, 54*(9), 1461-1474. doi:10.1080/10826084.2019.1586949

Zaller, N., Cheney, A. M., Curran, G. M., Booth, B. M., & Borders, T. F. (2016). The criminal justice experience of African American cocaine users in Arkansas. *Substance Use & Misuse, 51*(12), 1566-1576.
