# Supplementary material for: Understanding the health and well-being impacts and implementation barriers and facilitators of legally-mandated non-custodial drug and alcohol treatment for justice-involved adults: a qualitative evidence synthesis
Source: Health Justice. 2025 Oct 1;13:58. doi: 10.1186/s40352-025-00361-5 (PMC12487214; doi:10.1186/s40352-025-00361-5)
Supplement: Supplementary file 10 — Additional file 10. Methodological limitations for methodological domains assessed using Critical Appraisal Skills Programme (CASP). Description of data: a table showing the judgements for each CASP methodological domain for each included study [file 40352_2025_361_MOESM10_ESM.docx]

## Additional file 10. Methodological limitations for methodological domains assessed using Critical Appraisal Skills Programme (CASP)

| **Study** | **1. Clear statement of aims of the research?** | **2. Qualitative method-ology approp-riate?** | **3. Research design appropriate to address research aims?** | **4. Recruitment strategy appropriate to aims of the research?** | **5. Data collected in a way that addressed the research issue?** | **6. Relationship between researcher & participants adequately considered?** | **7. Ethical issues considered?** | **8. Data analysis sufficiently rigorous?** | **9. Clear statement of findings?** | **10. Overall assessment of method-ological limitations** | **Explanatory comments** |
| --- | --- | --- | --- | --- | --- | --- | --- | --- | --- | --- | --- |
| Bates 2009 | Yes | Yes | Yes | Yes | Yes | Yes | Yes | Yes | Yes | Low | This thesis addressed key domains - data collection, analysis and ethical considerations are described in detail. Further consideration to recruitment methods and sampling could have been addressed but we had only minor concerns. |
| Bevli 2018 | Yes | Yes | Yes | No | Yes | Yes | Yes | Can't tell | Yes | Moderate | There are concerns about two key domains- the recruitment strategy and analysis of the data. A key concern was that recruitment seemed to exclude key individuals who should have participated, as it only included Hispanic people who were proficient in English. |
| Datchi 2017 | Yes | Yes | Can't tell | Can't tell | Can't tell | Can't tell | Can't tell | No | No | Moderate | There was no detail provided on the analysis process. There was an absence of reporting of methods in this book chapter so it was difficult to assess methodological limitations, meaning we assessed it as 'moderate limitations' overall. |
| Dickson-Gomez 2022 | Yes | Yes | Can't tell | Yes | Yes | No | Yes | Yes | Yes | Low | We had no or minor concerns about recruitment, ethics, data collection and analysis, and findings. There was not enough detail to assess limitations in terms of the choice of research design and a lack of consideration of researcher-participant relationships. low limitations are probably mainly due to lack of / poor reporting. |
| Eley 2002** | Yes | Yes | Can't tell | Can't tell | Can't tell | Can't tell | Can't tell | Can't tell | Yes | Moderate | There is a lack of detailed reporting of methods given this is a report rather than an academic publication meaning it was hard to judge the limitations. All domains, except for findings, lacked information on which to base our assessments. Same study as McIvor 2006, 2009 |
| McIvor 2006** | Yes | Yes | Can't tell | Can't tell | Can't tell | Can't tell | Can't tell | Can't tell | Yes | Moderate | There was unclear reporting of all domains except findings. Because there is an absence of reporting of most domains we have chosen moderate rather than high limitations since it was difficult to assess. Same study as Eley 2002, McIvor 2009 |
| McIvor 2009** | Yes | Yes | Can't tell | Can't tell | Can't tell | Can't tell | Can't tell | Can't tell | Yes | Moderate | There are concerns around recruitment, and lack of detailed reporting of, or justification for, methods, and researcher potential bias. There is no evidence of ethical considerations being addressed. Overall a lack of detailed reporting made assessing methodological limitations difficult. Same study as Eley 2002, McIvor 2006 |
| Fischer 2007 | Yes | Yes | Yes | Yes | Yes | No | Can't tell | Yes | No | HIgh | We had no or minor concerns about the research design, recruitment, data collection and analysis. A major concern was the statement of findings was not clear e.g. theme descriptions were thinly described, no discussion of credibility. There was a lack of detail in terms of ethical considerations. The relationship between researcher and participants was not adequately considered. |
| Francis 2014 | Yes | Yes | Yes | Yes | Yes | Can't tell | No | Yes | Yes | Low | All domains except ethical issues had no or low concerns. There was enough information to assess limitations, except for participant and researcher relationships and reflexivity as these were not reported. We had concerns over the ethical issues of the researcher reviewing drug court client charts containing sensitive information to locate potential participants and then phoning all eligible clients- the ethical justification for this approach is not explained. |
| Fulkerson 2012 | Yes | Yes | Can't tell | Yes | Yes | Yes | Yes | Yes | Yes | Low | We had no or low concerns for all key domains except there was limited discussion of the research design. The authors do not report clear aims and objectives but the research appears robust and was methodologically justified. There is no evidence of ethical approval being sought but there is evidence of ethical considerations within the methods. |
| Gallagher & Wahler 2018* | Yes | Yes | Yes | Yes | Yes | Yes | No | Can't tell | Yes | Moderate | We had no or minor concerns about the research design, recruitment, data collection and findings. There was a lack of information in terms of ethical considerations (consent etc) and a lack of detail in terms of analysis. Same study as Gallagher, Nordberg & Dibley 2019 |
| Gallagher, Nordberg & Dibley 2019* | Yes | Yes | Yes | Yes | Yes | Yes | No | Can't tell | Yes | Moderate | We had no or low concerns regarding the research design, recruitment, researcher-participant relationships, data collection, and findings. Research methods were overall written in brief with scant attention to ethical considerations. Data analysis lacked detail and the thematic analysis had only two themes. Same study as Gallagher & Wahler 2018 |
| Gallagher, Wahler, Minasian & Edwards 2019 | Yes | Yes | Yes | Can't tell | Yes | No | No | Yes | Yes | Moderate | We had no or minor concerns about the research design, data collection and analysis and findings. There was a lack of information about recruitment. The researcher-participant relationship was not adequately considered. We had concerns about ethics - ethical approval was granted but ethical issues related to recruitment, informed consent and procedures were not explained or justified e.g. justice-involved people were approached directly after appearing in court and took part in a focus group straight away after agreement and they somehow selected people with a diagnosed substance use disorder. |
| Garcia 2019 | Yes | Yes | Can't tell | Yes | Yes | No | Yes | Can't tell | Yes | Moderate | We had no or minor concerns about recruitment, ethics, data collection and findings. There was insufficient reporting of the data analysis to assess limitations. Authors did not give a rationale for their research design. The relationship between researcher and participants was not considered. |
| Hamilton 2019 | Yes | Yes | Can't tell | Yes | Yes | No | Yes | Yes | Yes | Low | There were no or minor concerns about recruitment, ethics, data collection and analysis, findings. There was a lack of detail about the chosen study design but overall the methodological approach was strong. The relationship between researcher and participants was not adequately considered. |
| Harrell 1998 | Yes | Yes | Yes | Can't tell | Can't tell | No | Yes | Can't tell | No | High | We had no or minor concerns about the design and ethics. There was a lack of detail on the recruitment strategy information, data collection and data analysis which made assessing limitations difficult. A limited overview of findings (main focus on quantitative findings) - direct quotes and narrative of participant views and opinions but no clear qualitative findings. The relationship between researcher and participants was not adequately considered. |
| Kennedy-Hendricks 2021 | Yes | Yes | Can't tell | No | Yes | No | No | Can't tell | No | High | We had concerns regarding recruitment, findings, ethics, and the relationship between researcher and participants. A lack of detail meant we could not assess the appropriateness of the research design or assess the data analysis. We were unconvinced of the rigour of this study. |
| Kerr 2011 | Yes | Yes | Yes | Yes | Yes | Can't tell | Yes | Can't tell | Yes | Low | Use of appropriate research design and justification for this, detailed data collection methods and qualitative analysis. Low or minor concerns regarding recruitment methods and findings (omission of validation of findings). No exploration of relationship between researcher and participants. |
| Kouimtsidis 2007 | Yes | Yes | Yes | Can't tell | Yes | No | Can't tell | Can't tell | Yes | Moderate | We had no concerns over research design, data collection or statement of findings. Insufficient information to assess ethical study conduct and limitations of data analysis (no in-depth description of the analysis process). Relationship between researcher and participants not adequately considered. Lack of detailed reporting throughout made assessing some domains difficult. |
| Maddox 2023 | Yes | Can't tell | Can't tell | Can't tell | Can't tell | No | No | No | No | High | Very little detail and justification of methods. The research design, data analysis, findings and ethics raised concerns. |
| Moore 2017 | Yes | Yes | Can't tell | Yes | Yes | No | Yes | Yes | Yes | Low | No or minor concerns in most domains. There was lack of detail about the research design and no discussion of the researcher-participant relationship, but otherwise clear information was given. |
| Morse 2014*** | Yes | Yes | Can't tell | Yes | Yes | No | No | Yes | Yes | Moderate | No or low concerns regarding recruitment strategy, data collection and analysis and the statement of findings. Ethical considerations were not fully detailed; exploration of the relationship between researcher and participants was missing. There was insufficient detail to assess limitations of the research design. Limited details of ethical issues for a vulnerable population, but consent was not sought raising ethical concerns. |
| Morse 2015*** | Yes | Yes | Can't tell | Yes | Yes | No | No | Yes | Yes | Moderate | No or low concerns regarding recruitment strategy, data collection and analysis and the statement of findings. The authors did not justify the choice and appropriateness of the research design. Limited details of ethical issues for a vulnerable population, but consent was not sought raising ethical concerns. Relationship between researchers and participants not adequately considered. |
| Murphy 2011 | Yes | Yes | Yes | Can't tell | Can't tell | No | No | No | No | High | We identified limitations or a lack of information for most domains. There were concerns over lack of ethical discussions, a lack of detail to assess limitations of data collection and analysis and findings. It is not mentioned how the participants were selected, no exploration of the relationship between researcher and participants. |
| Powell 2012 | Yes | Yes | Can't tell | Can't tell | Yes | Yes | Can't tell | No | Yes | Moderate | Methodology and design were poorly described with little justification. Ethical issues, while taken into consideration, did not have any kind of formal institutional approval (once NHS ethics was deemed inappropriate), nor was informed consent detailed, although participation was shown to be voluntary and confidential. Method of analysis is unfamiliar and thinly explained. There are issues with some of the key areas in terms of recruitment strategy, data analysis and researcher bias as detail is missing. |
| Ricketts 2005 | Yes | Yes | Can't tell | Can't tell | Yes | Yes | No | Can't tell | Yes | Moderate | There is a lack of detail in terms of data analysis and ethics. The authors talk about grounded theory but it is not clear that this is the approach that has actually been taken. |
| Salzman 2023 | Yes | Yes | Can't tell | Yes | Yes | Can't tell | Yes | Can't tell | Yes | Moderate | There was a lack of information to assess limitations of research design, researcher bias and data analysis. No or minor concerns about other domains. |
| Sarmiento 2019 | Yes | Yes | Yes | Yes | Yes | No | No | Can't tell | No | High | There was not a clear statement of findings (research questions not answered, little evidence of opposing arguments, difficult to identify study findings and differentiate from literature) and researcher bias/reflexivity was not discussed. There was a lack of detail with regards to how data analysis was undertaken and so we could not determine if it was appropriate/undertaken well. We had ethical concerns about paying drug using clients after asking them about drug use - this was not justified by the authors. |
| Schiff 2010 | No | Yes | Yes | Can't tell | Can't tell | No | No | No | Yes | High | Methodologically this study lacks key aspects - limited justification for methods and study design, no ethical considerations are provided or potential researcher influences or analysis. Recruitment and data collection lacked detail so limitations were difficult to assess. |

Key* Publications reporting the same study

** Publications reporting the same study

*** Publications reporting the same study

## References

Bates, T. J. (2009). *Drug court: Breaking the black magic spell of drug addiction for women: A qualitative study.* (Doctor of Philosophy). The University of Utah, Dissertation Abstracts International Section A: Humanities and Social Sciences.

Bevli, S. (2018). *Effectiveness of the substance abuse and crime prevention act: the experiences of Hispanic residents.* (Doctor of Psychology). University of the Rockies, Dissertation Abstracts International: Section B: The Sciences and Engineering.

Datchi, C. C., & Ancis, J. R. (2017). Women and adult drug treatment courts: Surveillance, social conformity, and the exercise of agency. In J. R. Ancis (Ed.), *Gender, psychology, and justice: The mental health of women and girls in the legal system* (pp. 101-126). New York, NY: New York University Press; US.

Dickson-Gomez, J., Spector, A., Krechel, S., Li, J., Montaque, H. D. G., Ohlrich, J., . . . Weeks, M. (2022). Barriers to drug treatment in police diversion programs and drug courts: A qualitative analysis. *Am J Orthopsychiatry, 92*(6), 692-701. doi:10.1037/ort0000643

10.1037/ort0000643. Epub 2022 Oct 13.

Eley, S., Malloch, M., McIvor, G., Yates, R., & Brown, A. (2002). *The Glasgow drug court in action: the first six months*. Retrieved from Scotland:

Fischer, M., Geiger, B., & Hughes, M. E. (2007). Female recidivists speak about their experience in drug court while engaging in appreciative inquiry. *International Journal of Offender Therapy & Comparative Criminology, 51*(6), 703-722. doi:10.1177/0306624X07299304

10.1177/0306624X07299304. Epub 2007 Jul 5.

Francis, T. R., & Abel, E. M. (2014). Redefining success: A qualitative investigation of therapeutic outcomes for noncompleting drug court clients. *Journal of Social Service Research, 40*(3), 325-338. doi:10.1080/01488376.2013.875094

Fulkerson, A., Keena, L. D., & O'Brien, E. (2013). Understanding success and nonsuccess in the drug court. *International Journal of Offender Therapy & Comparative Criminology, 57*(10), 1297-1316. doi:10.1177/0306624X12447774

10.1177/0306624X12447774. Epub 2012 May 28.

Gallagher, J. R., Nordberg, A., & Dibley, A. R. (2019). Improving graduation rates for African Americans in drug court: Importance of human relationships and barriers to gaining and sustaining employment. *J Ethn Subst Abuse, 18*(3), 387-401. doi:10.1080/15332640.2017.1381661

10.1080/15332640.2017.1381661. Epub 2017 Nov 16.

Gallagher, J. R., & Wahler, E. A. (2018). Racial Disparities in Drug Court Graduation Rates: The Role of Recovery Support Groups and Environments. *Journal of Social Work Practice in the Addictions, 18*, 113-127.

Gallagher, J. R., Wahler, E. A., Minasian, R. M., & Edwards, A. (2019). Treating opioid use disorders in drug court: participants’ views on using medication-assisted treatments (MATs) to support recovery. *International Criminal Justice Review, 29*(3), 249-261. doi:10.1177/1057567719846227

Garcia, R. A., Kenyon, K. H., Brolan, C. E., Coughlin, J., & Guedes, D. D. (2019). Court as a health intervention to advance Canada's achievement of the sustainable development goals : a multi-pronged analysis of Vancouver's Downtown Community Court. *Global Health, 15*(1), 80. doi:10.1186/s12992-019-0511-9

10.1186/s12992-019-0511-9.

Hamilton, L. (2019). *Health-related quality of life among community-based offenders: How 'well-being' affects substance abuse treatment engagement.* (Doctor of Philosophy). Temple University, Dissertation Abstracts International Section A: Humanities and Social Sciences.

Harrell, A., Cavanagh, S., & Roman, J. (1998). *Findings from the evaluation of the D.C. Superior Court drug intervention program*. Retrieved from US:

Kennedy-Hendricks, A., Bandara, S., Merritt, S., Barry, C. L., & Saloner, B. (2021). Structural and organizational factors shaping access to medication treatment for opioid use disorder in community supervision. *Drug Alcohol Depend, 226*, 108881. doi:10.1016/j.drugalcdep.2021.108881

10.1016/j.drugalcdep.2021.108881. Epub 2021 Jun 26.

Kerr, J., Tompkins, C., Tomaszewski, W., Dickens, S., Grimshaw, R., Wright, N., & Barnard, M. (2011). *The dedicated drug courts pilot evaluation process study*. Retrieved from Ministry of Justice, UK: <www.justice.gov.uk/publications/research.htm>

Kouimtsidis, C., Reynolds, M., & Asamoah, V. (2007). Treatment or prison: service user and staff experiences of drug treatment and testing orders. *Psychiatric Bulletin, 31*(12), 463-466. doi:10.1192/pb.bp.107.014548

Maddox, M. E. (2023). *The effectiveness of drug treatment court: Participants' recommendations for improvement of the drug treatment court diversion program.* (Doctor in Psychology). William James College, Dissertation Abstracts International: Section B: The Sciences and Engineering.

McIvor, G. (2009). Therapeutic jurisprudence and procedural justice in Scottish Drug Courts. *Criminology & Criminal Justice, 9*(1), 29–49. doi:10.1177/1748895808099179

McIvor, G., Barnsdale, L., Eley, S., Malloch, M., Yates, R., & Brown, A. (2006). *The operation and effectiveness of the Scottish drug court pilots*. Retrieved from Scotland:

Moore, K. A., Barongi, M. M., & Rigg, K. K. (2017). The Experiences of Young Adult Offenders Who Completed a Drug Court Treatment Program. *Qual Health Res, 27*(5), 750-758. doi:10.1177/1049732316645782

10.1177/1049732316645782. Epub 2016 Jul 10.

Morse, D. S., Cerulli, C., Bedell, P., Wilson, J. L., Thomas, K., Mittal, M., . . . Chin, N. (2014). Meeting health and psychological needs of women in drug treatment court. *J Subst Abuse Treat, 46*(2), 150-157. doi:10.1016/j.jsat.2013.08.017

10.1016/j.jsat.2013.08.017. Epub 2013 Sep 24.

Morse, D. S., Silverstein, J., Thomas, K., Bedel, P., & Cerulli, C. (2015). Finding the loopholes: a cross-sectional qualitative study of systemic barriers to treatment access for women drug court participants. *Health & Justice, 3*, 12. doi:10.1186/s40352-015-0026-2

10.1186/s40352-015-0026-2. Epub 2015 Jun 17.

Murphy, J. (2011). Drug court as both a legal and medical authority. *Deviant Behavior, 32*(3), 257-291.

Powell, C. L. (2012). *Coerced drug treatment in England and Wales: An evaluation of Drug Treatment and Testing Orders in one locality.* (PhD Psychology). University of Leicester, UK.

Ricketts, T., Bliss, P., Murphy, K., & Brooker, C. (2005). Engagement with drug treatment and testing orders: A qualitative study. *Addiction Research & Theory, 13*(1), 65-78. doi:10.1080/16066350512331328168

Salzman, H. J. (2023). *Motherhood and substance use: An examination of societal pressures in the motivation to complete court-ordered drug treatment and to desist from future criminal activity and drug use.* (Doctor of Philosophy). University of Manchester, Dissertation Abstracts International: Section B: The Sciences and Engineering, UK.

Sarmiento, E., Seear, K., & Fraser, S. (2019). Enacting alcohol and other drug (Testing)-related harms in an Australian drug court. *Contemporary Drug Problems, 46*(3), 282-230.

Schiff, R., & Waegemakers Schiff, J. (2010). Housing needs and preferences of relatively homeless Aboriginal women with addiction. *Social Development Issues, 32*(3), 65-76.
