# Supplementary material for: Understanding the health and well-being impacts and implementation barriers and facilitators of legally-mandated non-custodial drug and alcohol treatment for justice-involved adults: a qualitative evidence synthesis
Source: Health Justice. 2025 Oct 1;13:58. doi: 10.1186/s40352-025-00361-5 (PMC12487214; doi:10.1186/s40352-025-00361-5)
Supplement: Supplementary file 11 — Additional file 11. Studies contributing to categories. Description of data: details of studies, and their key characteristics, that contributed to each of the overarching categories under which synthesis findings are organised [file 40352_2025_361_MOESM11_ESM.docx]

# Additional file 11. Included studies contributing to overarching categories

| **Thematic category** | **Contributing studies & key study characteristics** |
| --- | --- |
| Impact of treatment orders on health and well-being | The data supporting this category came from 9 publications representing 8 studies published between 2002 and 2023 (Bates 2009; Datchi 2017; Eley 2002; Fischer 2007; Hamilton 2019; Moore 2017; Morse 2014; Morse 2015; Salzman 2023). The studies were conducted in the USA (n=7) and Scotland (n=1). |
| Impact of treatment orders on substance use | The data supporting this category came from 13 publications representing 11 studies published between 2002 and 2023 (Bates 2009; Bevli 2018; Eley 2002; Francis 2014; Gallagher 2018; Gallagher, Nordberg & Dibley 2019; Gallagher, Wahler et al 2019; McIvor 2006; Moore 2017; Powell 2012; Ricketts 2005; Salzman 2023; Schiff 2010). The studies were conducted in the USA (n=5), Scotland (n=1), England (n=2) and Canada (n=1). Data focused mainly on the recipients of treatment orders with very little data reported from legal staff (n=2) or treatment providers (n=1). |
| Motivation to stop substance use | 18 publications representing 17 studies published between 1998 and 2023 provided insight into some of the motivations to engage with the mandated treatment (Bates 2009; Bevli 2018; Datchi 2017; Eley 2002; Fischer 2007; Francis 2014; Fulkerson 2013; Gallagher, Nordberg & Dibley 2019; Harrell 1998; Kerr 2011; Kouimtsidis 2007; Maddox 2023; McIvor 2006; Moore 2017; Morse 2014; Powell 2012; Ricketts 2005; Salzman 2023;). There was no discussion by staff who were actively delivering a Treatment Order staff (for example health professionals' social workers) in any of the articles. Only three of the articles (one from Scotland and two from USA) provided any discussion of the views of the legal staff in relation to motivation to change and their views were similar (Bevli 2018; Eley 2002; Fisher 2007). |
| Life context of justice-involved adults who use substances | The data supporting this category came from 17 publications representing 17 studies published between 2002 and 2023 (Bates 2009; Bevli 2018; Datchi 2017; Dickson-Gomez 2022; Eley 2002; Fischer 2007; Francis 2014; Gallagher, Nordberg et al 2019; Garcia 2019; Hamilton 2019; Maddox 2023; Moore 2017; Morse 2014; Murphy 2011; Powell 2012; Salzman 2023; Schiff 2010). The studies were conducted in the USA, Canada, Scotland, and England. The data mainly reported the views of justice-involved adults, with a small amount of data from legal staff and treatment providers. |
| Ideology and defining treatment success | The data supporting this category came from 12 publications representing 11 studies published between 2002 and 2022 (Datchi 2017; Dickson-Gomez 2022; Eley 2002; Fischer 2007; Gallagher, Wahler et al 2019; Kennedy-Hendricks 2021; Kerr 2011; Kouimtsidis 2007; McIvor 2006; Morse 2014; Murphy 2011; Powell 2012). The studies were conducted in the USA, Scotland, and England. The data reported the views of mainly legal staff, but also justice-involved adults, and treatment provider staff. |
| Tension between punishment and therapeutic treatment | The data supporting this category came from 12 publications representing 11 studies published between 1998 and 2023 (Bates 2009; Bevli 2018; Datchi 2017; Eley 2002; Fulkerson 2012; Fischer 2007; Harrell 1998; Kouimitsdis 2007; Maddox 2023; McIvor 2006; Murphy 2011; Salzman 2023). The studies were conducted in USA, Scotland, and England. The data reported mainly the views of justice-involved adults, with less data on legal staff and treatment provider staff. |
| Attitudes to treatments | The data supporting this category come from nine publications representing eight?studies published between 1998 and 2023 (Eley 2002; Kennedy-Hendricks 2021; Fischer 2007; Gallagher 2018; Gallagher, Wahler et al 2019; Harrell 1998; McIvor 2006; Maddox 2023; Powell 2012). The studies were conducted in USA and Scotland. The data mainly reported the views of justice-involved adults, and treatment provider staff with some data from legal staff. |
| Relationships between justice-involved adults and staff | The data supporting this category came from 13 publications representing 11 studies published between 2002 and 2023 (Bates 2009; Bevli 2018; Eley 2002; Fischer 2007; Francis 2014; Fulkerson 2013; Gallagher, Nordberg & Dibley 2019; Kerr 2011; Maddox 2023; McIvor 2006; McIvor 2009; Moore 2017; Ricketts 2005 ). The studies were conducted in the USA, England, and Scotland. The data reported the views of mainly justice-involved men and women, with scant views from judicial staff and treatment provider staff. |
| Relationships with family and friends | The data supporting this category come from 17 publications representing 14 studies published between 1998 and 2023 (Bates 2009; Bevli 2018; Datchi 2017; Eley 2002; Fischer 2007; Fulkerson 2013; Garcia 2019; Gallagher, Nordberg & Dibley 2019; Hamilton 2019; Harrell 1998; Maddox 2023; McIvor 2006; McIvor 2009; Moore 2017; Morse 2014; Morse 2015; Ricketts 2005). The studies were conducted in Scotland, England, the USA, and Canada. The data mainly reported the views of justice-involved adults with some data from legal staff, and very little from treatment provider staff. Fifteen papers had some discussion by justice-involved individuals in relation to family relationships. The relationship between family members and the justice-involved individual was not discussed by any of the non-judicial legal staff (e.g. police officers, probation officers) or other stakeholders (for example: unpaid peers, volunteers, mentors). Only three publications from the USA which focused on justice-involved females provided some views from treatment providers of the TO in relation to family relationships (Morse 2014 & 2015; Garcia 2019). |
| Organisational and community barriers and facilitators | The data supporting this category come from 15 publications representing 14 studies published between 2002 and 2023 (Eley 2002; Fischer 2007; Fulkerson 2012; Garcia 2019; Harrell 1998; Hamilton 2019; Kennedy-Hendricks 2021; Kerr 2011; Kouimtsidis 2007; Maddox 2023; McIvor 2006; Morse 2014; Ricketts 2005; Salzman 2023; Schiff 2010). The studies were conducted in the USA, Canada, Scotland and England. The data reported the views of justice-involved adults, legal staff, and treatment provider staff. |
| Homelessness, housing, employment, and training | The data supporting this category come from 17 publications representing 16 studies published between 1998 and 2023 (Bevli 2018; Dickson-Gomez 2022; Eley 2002; Fischer 2007; Francis 2014; Fulkerson 2012, Gallagher, Nordberg & Dibley 2019; Hamilton 2019 ; Harrell 1998; Maddox 2023; Morse 2014 & 2015; Moore 2017; Murphy 2011; Powell 2012; Salzman 2023; Schiff 2010). The studies were conducted in the USA, Scotland, England, and Canada. |
| Ethnicity/race | The data supporting this category come from three publications representing three studies published between 2017 and 2019 (Bevli 2018; Datchi 2017; Gallagher, Nordberg et al., 2019; Gallagher 2018). |

(Bates, 2009; Bevli, 2018; Datchi & Ancis, 2017; Dickson-Gomez et al., 2022; Eley, Malloch, McIvor, Yates, & Brown, 2002; Fischer, Geiger, & Hughes, 2007; Francis & Abel, 2014; Fulkerson, Keena, & O'Brien, 2012; Gallagher, Nordberg, & Dibley, 2019; Gallagher & Wahler, 2018; Gallagher, Wahler, Minasian, & Edwards, 2019; Garcia, Kenyon, Brolan, Coughlin, & Guedes, 2019; Hamilton, 2019; Harrell, Cavanagh, & Roman, 1998; Kennedy-Hendricks, Bandara, Merritt, Barry, & Saloner, 2021; Kerr et al., 2011; Kouimtsidis, Reynolds, & Asamoah, 2007; Maddox, 2023; McIvor, 2009; McIvor et al., 2006; Moore, Barongi, & Rigg, 2017; Morse et al., 2014; Morse, Silverstein, Thomas, Bedel, & Cerulli, 2015; Murphy, 2011; Powell, 2012; Ricketts, Bliss, Murphy, & Brooker, 2005; Salzman, 2023; Schiff & Waegemakers Schiff, 2010)

## References

Bates, T. J. (2009). *Drug court: Breaking the black magic spell of drug addiction for women: A qualitative study.* (Doctor of Philosophy). The University of Utah, Dissertation Abstracts International Section A: Humanities and Social Sciences.

Bevli, S. (2018). *Effectiveness of the substance abuse and crime prevention act: the experiences of Hispanic residents.* (Doctor of Psychology). University of the Rockies, Dissertation Abstracts International: Section B: The Sciences and Engineering.

Datchi, C. C., & Ancis, J. R. (2017). Women and adult drug treatment courts: Surveillance, social conformity, and the exercise of agency. In J. R. Ancis (Ed.), *Gender, psychology, and justice: The mental health of women and girls in the legal system* (pp. 101-126). New York, NY: New York University Press; US.

Dickson-Gomez, J., Spector, A., Krechel, S., Li, J., Montaque, H. D. G., Ohlrich, J., . . . Weeks, M. (2022). Barriers to drug treatment in police diversion programs and drug courts: A qualitative analysis. *Am J Orthopsychiatry, 92*(6), 692-701. doi:10.1037/ort0000643

10.1037/ort0000643. Epub 2022 Oct 13.

Eley, S., Malloch, M., McIvor, G., Yates, R., & Brown, A. (2002). *The Glasgow drug court in action: the first six months*. Retrieved from Scotland:

Fischer, M., Geiger, B., & Hughes, M. E. (2007). Female recidivists speak about their experience in drug court while engaging in appreciative inquiry. *International Journal of Offender Therapy & Comparative Criminology, 51*(6), 703-722. doi:10.1177/0306624X07299304

10.1177/0306624X07299304. Epub 2007 Jul 5.

Francis, T. R., & Abel, E. M. (2014). Redefining success: A qualitative investigation of therapeutic outcomes for noncompleting drug court clients. *Journal of Social Service Research, 40*(3), 325-338. doi:10.1080/01488376.2013.875094

Fulkerson, A., Keena, L. D., & O'Brien, E. (2012). Understanding success and nonsuccess in the drug court. *International Journal of Offender Therapy & Comparative Criminology, 57*(10), 1297-1316. doi:10.1177/0306624X12447774

10.1177/0306624X12447774. Epub 2012 May 28.

Gallagher, J. R., Nordberg, A., & Dibley, A. R. (2019). Improving graduation rates for African Americans in drug court: Importance of human relationships and barriers to gaining and sustaining employment. *J Ethn Subst Abuse, 18*(3), 387-401. doi:10.1080/15332640.2017.1381661

10.1080/15332640.2017.1381661. Epub 2017 Nov 16.

Gallagher, J. R., & Wahler, E. A. (2018). Racial Disparities in Drug Court Graduation Rates: The Role of Recovery Support Groups and Environments. *Journal of Social Work Practice in the Addictions, 18*, 113-127.

Gallagher, J. R., Wahler, E. A., Minasian, R. M., & Edwards, A. (2019). Treating opioid use disorders in drug court: participants’ views on using medication-assisted treatments (MATs) to support recovery. *International Criminal Justice Review, 29*(3), 249-261. doi:10.1177/1057567719846227

Garcia, R. A., Kenyon, K. H., Brolan, C. E., Coughlin, J., & Guedes, D. D. (2019). Court as a health intervention to advance Canada's achievement of the sustainable development goals : a multi-pronged analysis of Vancouver's Downtown Community Court. *Global Health, 15*(1), 80. doi:10.1186/s12992-019-0511-9

10.1186/s12992-019-0511-9.

Hamilton, L. (2019). *Health-related quality of life among community-based offenders: How 'well-being' affects substance abuse treatment engagement.* (Doctor of Philosophy). Temple University, Dissertation Abstracts International Section A: Humanities and Social Sciences.

Harrell, A., Cavanagh, S., & Roman, J. (1998). *Findings from the evaluation of the D.C. Superior Court drug intervention program*. Retrieved from US:

Kennedy-Hendricks, A., Bandara, S., Merritt, S., Barry, C. L., & Saloner, B. (2021). Structural and organizational factors shaping access to medication treatment for opioid use disorder in community supervision. *Drug Alcohol Depend, 226*, 108881. doi:10.1016/j.drugalcdep.2021.108881

10.1016/j.drugalcdep.2021.108881. Epub 2021 Jun 26.

Kerr, J., Tompkins, C., Tomaszewski, W., Dickens, S., Grimshaw, R., Wright, N., & Barnard, M. (2011). *The dedicated drug courts pilot evaluation process study*. Retrieved from Ministry of Justice, UK: [www.justice.gov.uk/publications/research.htm](https://stir-my.sharepoint.com/personal/eff2_stir_ac_uk/Documents/NESSIE%20NIHR%20Evidence%20Synthesis%20Group%20Todhunter%202022/Treatment%20order%20review%202023/TO%20Qual%20Review%20Article/www.justice.gov.uk/publications/research.htm)

Kouimtsidis, C., Reynolds, M., & Asamoah, V. (2007). Treatment or prison: service user and staff experiences of drug treatment and testing orders. *Psychiatric Bulletin, 31*(12), 463-466. doi:10.1192/pb.bp.107.014548

Maddox, M. E. (2023). *The effectiveness of drug treatment court: Participants' recommendations for improvement of the drug treatment court diversion program.* (Doctor in Psychology). William James College, Dissertation Abstracts International: Section B: The Sciences and Engineering.

McIvor, G. (2009). Therapeutic jurisprudence and procedural justice in Scottish Drug Courts. *Criminology & Criminal Justice, 9*(1), 29–49. doi:10.1177/1748895808099179

McIvor, G., Barnsdale, L., Eley, S., Malloch, M., Yates, R., & Brown, A. (2006). *The operation and effectiveness of the Scottish drug court pilots*. Retrieved from Scotland:

Moore, K. A., Barongi, M. M., & Rigg, K. K. (2017). The Experiences of Young Adult Offenders Who Completed a Drug Court Treatment Program. *Qual Health Res, 27*(5), 750-758. doi:10.1177/1049732316645782

10.1177/1049732316645782. Epub 2016 Jul 10.

Morse, D. S., Cerulli, C., Bedell, P., Wilson, J. L., Thomas, K., Mittal, M., . . . Chin, N. (2014). Meeting health and psychological needs of women in drug treatment court. *J Subst Abuse Treat, 46*(2), 150-157. doi:10.1016/j.jsat.2013.08.017

10.1016/j.jsat.2013.08.017. Epub 2013 Sep 24.

Morse, D. S., Silverstein, J., Thomas, K., Bedel, P., & Cerulli, C. (2015). Finding the loopholes: a cross-sectional qualitative study of systemic barriers to treatment access for women drug court participants. *Health & Justice, 3*, 12. doi:10.1186/s40352-015-0026-2

10.1186/s40352-015-0026-2. Epub 2015 Jun 17.

Murphy, J. (2011). Drug court as both a legal and medical authority. *Deviant Behavior, 32*(3), 257-291.

Powell, C. L. (2012). *Coerced drug treatment in England and Wales: An evaluation of Drug Treatment and Testing Orders in one locality.* (PhD Psychology). University of Leicester, UK.

Ricketts, T., Bliss, P., Murphy, K., & Brooker, C. (2005). Engagement with drug treatment and testing orders: A qualitative study. *Addiction Research & Theory, 13*(1), 65-78. doi:10.1080/16066350512331328168

Salzman, H. J. (2023). *Motherhood and substance use: An examination of societal pressures in the motivation to complete court-ordered drug treatment and to desist from future criminal activity and drug use.* (Doctor of Philosophy). University of Manchester, Dissertation Abstracts International: Section B: The Sciences and Engineering, UK.

Schiff, R., & Waegemakers Schiff, J. (2010). Housing needs and preferences of relatively homeless Aboriginal women with addiction. *Social Development Issues, 32*(3), 65-76.
