# Supplementary material for: Understanding the health and well-being impacts and implementation barriers and facilitators of legally-mandated non-custodial drug and alcohol treatment for justice-involved adults: a qualitative evidence synthesis
Source: Health Justice. 2025 Oct 1;13:58. doi: 10.1186/s40352-025-00361-5 (PMC12487214; doi:10.1186/s40352-025-00361-5)
Supplement: Supplementary file 12 — Additional file 12. Summary of Qualitative Findings Table. Description of data: GRADE-CERQual Assessment of confidence summaries of the review findings, the overall CERQual assessments, and an explanation of each CERQual assessment [file 40352_2025_361_MOESM12_ESM.docx]

# Additional file 12. Summary of Qualitative Findings Table

| **#** | **Summarised review finding** | **GRADE-CERQual Assessment of confidence** | **Explanation of GRADE-CERQual Assessment** | **References** |
| --- | --- | --- | --- | --- |
| **PERCEIVED MENTAL HEALTH IMPACTS OF TREATMENT ORDERS** | | | | |
| 1 | Justice involved adults (men, women, and young adults) in drug courts in the United States of America perceived some positive impacts of treatment orders on their mental health and emotional well-being including an improved sense of self (Moore 2017) and a greater ability to deal with their emotions (Bates 2009). Treatment providers also perceived participants had improved mental health issues (Morse 2014). | Moderate confidence | Minor concerns regarding methodological limitations, Minor concerns regarding coherence, Moderate concerns regarding adequacy, and Moderate concerns regarding relevance | Bates 2009; Moore et al. 2017; Morse et al. 2014; |
| 2 | In drug courts in the United States of America (USA), justice-involved adults identified unintended negative consequences of mandated drug use treatments on their mental health and emotional well-being, especially women who were more likely to have dependent children and/or to have trauma. For instance, the use of time in prison as a sanction for drug use relapse or placement in residential treatment facilities that did not allow children contributed to the loss of custody of one’s children; this could compound women’s pre-existing trauma (Fischer 2007, Datchi 2017). Justice-involved men and women perceived that the stress of competing demands of family, work and treatment order requirements also negatively affected their mental health (Hamilton 2019). | Low confidence | Moderate concerns regarding methodological limitations, No/Very minor concerns regarding coherence, Serious concerns regarding adequacy, and Moderate concerns regarding relevance | Hamilton 2019; Fischer et al. 2007; Datchi et al. 2017; |
| **PERCEIVED PHYSICAL HEALTH IMPACTS OF TREATMENT ORDERS** | | | | |
| 3 | In drug courts in Scotland justice-involved adults and sheriffs (Scottish judges), pregnant women in a residential substance-abuse treatment facility and court staff in the USA reported improved health of justice-involved adults due to mandated drug use treatment (Eley 2002, Morse 2015, Salzman 2023). For example, women perceived it had saved their life by getting them off drugs and had helped them avoid sexually-transmitted diseases through stopping sex work which had funded their illicit drug use (Salzman 2023). Court staff (possibly treatment providers but it was not specified) felt that combining substance use treatment with housing for women and their children helped give women the stability to focus on their health and recovery (Morse 2015). | Low confidence | Moderate concerns regarding methodological limitations, Minor concerns regarding coherence, Serious concerns regarding adequacy, and Minor concerns regarding relevance | Eley et al. 2002; Morse et al. 2015; Salzman 2023; |
| **TREATMENT ORDERS AND DRUG USE** | | | | |
| 4 | Factors related to mandated treatment that some justice-involved adults (men, women and young adults from a range of ethnic groups) and judicial staff perceived to reduce and/or stabilise drug use in drug courts in the USA and Scotland or undertaking drug treatment and testing orders in England were: the provision of structure, routine and daily occupation e.g., frequently attending court, probation appointments and counselling; medical support including medication-assisted treatment and psychiatric treatment. Another possible aid was peer support e.g., from other justice-involved individuals in group counselling or in Alcoholics Anonymous and Narcotics Anonymous organisation support meetings (Bates 2009, Bevli 2018, Eley 2002, Francis 2014, Gallagher, Nordberg & Dibley 2019, McIvor 2006, Moore 2017). In addition, an individual’s readiness to stop or reduce illicit drug use was seen as important (Bates 2009, Bevli 2018, Datchi 2017; Eley 2002, Fischer 2007; Harrell 1998, Kouimtsidis 2007, Maddox 2023, McIvor 2006, Morse 2014, Powell 2012, Ricketts 2005, Salzman 2023). | High confidence | Minor concerns regarding methodological limitations, Minor concerns regarding coherence, Minor concerns regarding adequacy, and No/Very minor concerns regarding relevance | Bates 2009; Bevli 2018; Eley et al. 2002; Harrell et al. 1998; McIvor et al. 2006; Powell 2012; Fischer et al. 2007; Gallagher et al. 2019; Kouimtsidis et al. 2018; Moore et al. 2017; Morse et al. 2014; Datchi et al. 2017; Francis & Abel 2014; Maddox 2023; Ricketts et al. 2005; Salzman 2023; |
| 5 | In drug courts in the USA and Scotland and for drug treatment and testing orders in England, judicial staff and justice-involved adults often believed that participants needed to be ready to change their drug use behaviours for treatment orders to succeed (Bevli 2018; Datchi 2017; Eley 2002; Fischer 2007; Kouimtsidis 2007; McIvor 2006; Morse 2014; Powell 2012; Ricketts 2005). Motivating factors to be drug-free included justice-involved adults seeing the harm their drug use caused to others (Fulkerson 2013), and pregnancy and motherhood for pregnant women, although many continued to, or resumed, drug use after the birth of their children (Salzman 2023). However, many justice-involved adults highlighted that initially they agreed to attend drug court to avoid going to jail (Bevli 2018; Eley 2002; Harrell 1998; Maddox 2023; McIvor 2006; Salzman 2023). Other reasons were the worry about the impact of prison on their family or family life e.g., difficulties with caring for their children (Harrell 1998, Maddox 2023). Many justice-involved adults undertook the treatment order solely due to legal coercion rather than a desire to reduce/cease drug use (Bates 2009). Nonetheless, motivation to complete the treatment order could develop over time with encouragement from judicial staff (Bates 2009; Francis 2014; Gallagher, Nordberg & Dibley 2019; Kerr 2011; McIvor 2006) and through experiencing success at reducing/ceasing drug use (Moore 2017; Powell 2012). | High confidence | Minor concerns regarding methodological limitations, No/Very minor concerns regarding coherence, No/Very minor concerns regarding adequacy, and Minor concerns regarding relevance | Bates 2009; Bevli 2018; Eley et al. 2002; Harrell et al. 1998; McIvor et al. 2006; Powell 2012; Fischer et al. 2007; Fulkerson et al. 2013; Gallagher et al. 2019; Kouimtsidis et al. 2018; Moore et al. 2017; Morse et al. 2014; Datchi et al. 2017; Francis & Abel 2014; Kerr et al. 2011; Maddox 2023; Ricketts et al. 2005; Salzman 2023; |
| 6 | Barriers to justice-involved adults reducing or ceasing illicit drug use through a treatment order in drug courts in the USA, Canada and Scotland or under Drug Treatment and Testing Orders in England which affected their ability to engage with drug use treatment and substance use counsellors included their poor mental health (Francis 2014; Gallagher, Nordberg & Dibley 2019; Hamilton, 2019; Salzman, 2023); prior trauma and current challenging life circumstances (lack of housing, unemployment, financial problems, lack of childcare) (Bevli 2018; Datchi 2017; Eley 2002; Fischer 2007; Maddox 2023; McIvor 2006; Moore 2017; Salzman 2023); difficulties paying for their mandated treatment in the USA (Bates 2009, Maddox 2023); and living in neighbourhoods with high availability and use of illicit drugs (Murphy 2011). Another barrier was clashes between an individual’s job responsibilities and the strict drug court schedule that caused them to miss treatment appointments (Francis 2014). Also important was a lack of individual readiness to stop or reduce illicit drug use (Bates 2009; Bevli 2018; Datchi 2017; Eley 2002; Fischer 2007, Harrell 1998, Kouimtsidis 2007, Maddox 2023, McIvor 2006, Morse 2014, Powell 2012; Ricketts 2005, Salzman 2023). In addition, factors which contributed to relapse were delays in psychiatric treatment; perceived insufficient doses of methadone (a heroin-substitute treatment); and interactions with justice-involved peers who were selling drugs, talking about substance use, having relapses or demonstrating a lack of motivation to stop using which could trigger an individual's desire for drugs (Eley 2002, Morse 2014, Powell 2012, Schiff 2010). Family substance use, stressors and dynamics could also contribute to relapse (Bevli 2018 ; Datchi 2017; Eley 2002; Maddox 2023; Morse 2014; Morse 2015). | High confidence | Moderate concerns regarding methodological limitations, No/Very minor concerns regarding coherence, Minor concerns regarding adequacy, and Minor concerns regarding relevance | Bates 2009; Bevli 2018; Eley et al. 2002; Hamilton 2019; Harrell et al. 1998; McIvor et al. 2006; Murphy 2011; Powell 2012; Schiff & Waegemakers Schiff 2010; Fischer et al. 2007; Gallagher et al. 2019; Kouimtsidis et al. 2018; Moore et al. 2017; Morse et al. 2014; Morse et al. 2015; Datchi et al. 2017; Francis & Abel 2014; Maddox 2023; Ricketts et al. 2005; Salzman 2023; |
| **RELATIONSHIPS WITH JUDICIAL STAFF** | | | | |
| 7 | In drug courts in the USA and Scotland and for drug treatment and testing orders in England, judges and sheriffs (the name for judges in Scotland) and justice-involved adults often saw positive relationships between justice-involved adults and judicial staff as central to the sustainability and success of mandated treatment (Bates 2009, Bevli 2018, Eley 2002, Fischer 2007, Fulkerson 2013, Gallagher, Nordberg & Dibley 2019, Kerr 2011, Maddox 2023, McIvor 2009, Ricketts 2005). Justice-involved adults highly valued, and were motivated to succeed, when staff treated them with acceptance, support, fairness, respect, care and compassion (Bates 2009, Bevli 2018, Eley 2002, Fischer 2007, Gallagher, Nordberg & Dibley 2019, Maddox 2023, Ricketts 2005). Furthermore, sheriffs in Scottish drug courts perceived that direct, personal connection with treatment order participants could increase participants’ commitment and motivation to change (McIvor 2009). Some justice-involved adults highlighted the judge or sheriff in drug courts as a key “parental” figure of importance, (Bates 2009, Eley 2002, McIvor 2009). Seeing the same judge or sheriff repeatedly helped develop mutual trust, rapport and a relationship (Bates 2009, Fulkerson 2013, Kerr 2011, McIvor 2009). However, justice-involved adults sometimes distrusted and had conflict with drug court staff, often stemming from previous negative judiciary experiences (Bates 2009, Fischer 2007, Fulkerson 2013). | High confidence | Minor concerns regarding methodological limitations, No/Very minor concerns regarding coherence, No/Very minor concerns regarding adequacy, and Minor concerns regarding relevance | Bates 2009; Bevli 2018; Eley et al. 2002; Fischer et al. 2007; Fulkerson et al. 2013; Gallagher et al. 2019; Kerr et al. 2011; Maddox 2023; McIvor 2009; Ricketts et al. 2005; |
| **RELATIONSHIPS WITH TREATMENT PROVIDER STAFF** | | | | |
| 8 | Justice-involved adults in the USA and Scotland often perceived the personal, caring approach of drug court treatment team staff as a major contributory factor to achieving success while on a treatment order (Bevli 2018, Francis 2014, McIvor 2009, Moore 2017, Salzman 2023). Participants in the USA often preferred treatment staff who previously had had drug use problems because they saw them as more relatable and trustworthy, and this helped them engage with treatment (Fischer 2007, Hamilton 2019, Harrell 1998). In Scottish and USA drug courts, justice-involved adults reported lack of trust in, or feeling stigmatised by, the treatment team which could impact on progress, often resulting in missed appointments and non-compliance with treatment order requirements (McIvor 2006; Morse 2014). | Moderate confidence | No/Very minor concerns regarding methodological limitations, No/Very minor concerns regarding coherence, Moderate concerns regarding adequacy, and Minor concerns regarding relevance | Bevli 2018; Hamilton 2019; Harrell et al. 1998; McIvor et al. 2006; Fischer et al. 2007; Moore et al. 2017; Morse et al. 2014; Francis & Abel 2014; McIvor 2009; Salzman 2023; |
| **PERCEIVED IMPACTS OF TREATMENT ORDERS ON FAMILY RELATIONSHIPS** | | | | |
| 9 | Some justice-involved adults, including those who did not complete their treatment order, highlighted that attending drug court and treatment orders could aid with repairing family relationships which had been damaged by their past illicit drug use (Bates 2009, Eley 2002, Fischer 2007, Francis 2014, Fulkerson 2013, Garcia 2019, Maddox 2023, McIvor 2006, McIvor 2009, Moore 2017). However, many women within drug courts in Scotland and USA felt that treatment order requirements did not recognise their caring responsibilities and specific needs as mothers which negatively affected their relationships with their children (Eley 2002, Hamilton 2019, Harrell 1998, Maddox 2023, Morse 2015). For instance, justice-involved women in the US discussed the negative impact of being separated from their children when sent to a residential drug use treatment programme; they, and treatment providers, expressed an urgent need for facilities that accepted children (Fischer 2007, Maddox 2023, Morse 2014, Salzman 2023). Furthermore, some women reported that attending court became a competing priority with their family relationships (Datchi 2017). | High confidence | Minor concerns regarding methodological limitations, Minor concerns regarding coherence, No/Very minor concerns regarding adequacy, and No/Very minor concerns regarding relevance | Bates 2009; Eley et al. 2002; Hamilton 2019; Harrell et al. 1998; McIvor et al. 2006; Fischer et al. 2007; Fulkerson et al. 2013; Garcia et al. 2019; Moore et al. 2017; Morse et al. 2014; Morse et al. 2015; Datchi et al. 2017; Francis & Abel 2014; Maddox 2023; McIvor 2009; Salzman 2023; |
| **FAMILY AND PEER RELATIONSHIPS IMPACTS ON TREATMENT ORDER SUCCESS** | | | | |
| 10 | In Canada, the USA and Scotland family connections and peer support from other drug court participants could be an important source of support, strength and motivation helping individuals complete a treatment order and become abstinent from illicit drugs (Bevli 2018, Eley 2002, McIvor 2006, Garcia 2019, Gallagher, Nordberg & Dibley 2019, Maddox 2023, Moore 2017, Morse 2014,). However, judicial legal staff and justice-involved participants highlighted that participants’ family influences, stressors and dynamics, such as a partner or friends who used drugs or alcohol or experiencing domestic abuse, could negatively affect treatment order success, for example, by influencing a relapse ( Bevli 2018, Datchi 2017, Eley 2002, Garcia 2019, Maddox 2023, Morse 2014, Morse 2015). Courts could assist ability to comply with a treatment order by developing safety plans for domestic abuse victims (Garcia 2019). | High confidence | Minor concerns regarding methodological limitations, No/Very minor concerns regarding coherence, No/Very minor concerns regarding adequacy, and Minor concerns regarding relevance | Bevli 2018; Eley et al. 2002; McIvor et al. 2006; Gallagher et al. 2019; Garcia et al. 2019; Moore et al. 2017; Morse et al. 2014; Morse et al. 2015; Datchi et al. 2017; Maddox 2023; |
| **COERCION AND PUNISHMENT** | | | | |
| 11 | Coercion to undergo drug use treatment in drug courts and via drug treatment and testing orders could facilitate or interfere with the success of treatment orders in changing drug use behaviours which might then influence health outcomes in justice-involved adults in Scotland, England, and the USA. Justice-involved participants in drug courts perceived that being coerced to accept the legally-mandated treatment interfered with “therapeutic change” (addressing the underlying problems to facilitate recovery through therapy) (Bates 2009; Bevli 2018; Datchi 2017; McIvor 2006). Similarly in England, treatment providers felt they put in a lot of effort to overcome the therapeutic barrier of forced treatment as part of drug treatment and testing orders since participants should attend voluntarily (Kouimitsidis 2007). Some justice-involved adults undergoing drug treatment and testing orders in England perceived that precisely because treatment was forced it gave them the opportunity to benefit from it (Kouimitsdis 2007). Despite coercion to participate some justice-involved participants had positive views of mandated treatment as an opportunity to change drug use behaviours and recover (Eley 2002, Fischer 2007, McIvor 2006). | Moderate confidence | Moderate concerns regarding methodological limitations, No/Very minor concerns regarding coherence, Minor concerns regarding adequacy, and Moderate concerns regarding relevance | Bates 2009; Bevli 2018; Eley et al. 2002; McIvor et al. 2006; Fischer et al. 2007; Kouimtsidis et al. 2018; Datchi et al. 2017; |
| 12 | The threat of imprisonment for non-compliance with treatment orders could encourage justice-involved adults to accept accountability for their behaviour (Fulkerson 2013) but monitoring and legal sanctions were not enough to prevent rule breaking and promote active engagement in treatment (Fischer 2007; Harrell 1998). In drug courts in the USA and Scotland, justice-involved adults tended to perceive sanctions to be helpful and fair if they were not humiliating or excessive, for example, taking longer to complete the treatment order, amending or revoking the order, or a fine for breaking the rules were acceptable (Datchi 2017, Fischer 2007, McIvor 2006). In Midwest USA, justice-involved women saw a short stay in prison for minor violations, such as lying or being late, as too punitive (Datchi 2017). However, some justice-involved adults in the USA viewed all sanctions as purely punitive rather than helpful (Murphy 2011). Some participants responded well to a structured programme but it made recovery more difficult for others (Maddox 2023). Sanctions as part of a wider treatment and support programme helped some justice-involved adults comply with treatment order requirements which ultimately could help reduce or cease their illicit drug use (Datchi 2017, Fischer 2007, Maddox 2023, McIvor 2006). | Moderate confidence | Moderate concerns regarding methodological limitations, Minor concerns regarding coherence, Minor concerns regarding adequacy, and Minor concerns regarding relevance | Harrell et al. 1998; McIvor et al. 2006; Murphy 2011; Fischer et al. 2007; Fulkerson et al. 2013; Datchi et al. 2017; Maddox 2023; |
| **ORGANISATIONAL BARRIERS TO TREATMENT ORDER IMPLEMENTATION** | | | | |
| 13 | Organisational barriers to efficient operation of treatment orders and drug court processes were identified in the legal system and treatment provider organisations in Scotland, England, and the USA which negatively impacted justice-involved adults undertaking treatment orders. These included heavy staff workloads and caseloads e.g., for social workers, counsellors and probation officers; under-resourcing of treatment teams; staff illness; difficulties recruiting and retaining staff, particularly in newly-established drug courts; and a perceived need for more mental health staff (Eley 2002, Kerr 2011, Maddox 2023, McIvor 2006). Another barrier was related to the challenges of multi-disciplinary working: differences in organisational culture and perspectives contributed to communication problems between staff with different roles e.g., probation and legal staff, medical and social work staff (Eley 2002, Kerr 2011, Kouimtsidis 2007, McIvor 2006, Powell 2012). Staff perceived that poor communication and understanding between staff could affect referrals to drug court or result in treatment order participants receiving inconsistent messages about the rules and expectations, their appointments, and the services (Eley 2002; Kouimtsidis 2007; McIvor 2006). Lack of communication about a participants’ compliance with the treatment order was problematic for imposing sanctions (Kennedy-Hendricks 2021). In addition, delays between justice-involved adults being assessed and receiving drug use treatment (e.g. due to delayed drug test results) caused problems because the participants were highly likely to continue to use illicit drugs in breach of their order (Eley 2002, McIvor 2006, Maddox 2023, Ricketts 2005). Treatment providers perceived these issues, many of which could be resolved with additional resources, to adversely affect the quality of service (Eley 2002, McIvor 2006). | High confidence | Moderate concerns regarding methodological limitations, No/Very minor concerns regarding coherence, Minor concerns regarding adequacy, and Minor concerns regarding relevance | Eley et al. 2002; McIvor et al. 2006; Powell 2012; Kennedy-Hendricks et al. 2021; Kouimtsidis et al. 2018; Kerr et al. 2011; Maddox 2023; Ricketts et al. 2005; |

(Bates, 2009; Bevli, 2018; Datchi & Ancis, 2017; Dickson-Gomez et al., 2022; Eley, Malloch, McIvor, Yates, & Brown, 2002; Fischer, Geiger, & Hughes, 2007; Francis & Abel, 2014; Fulkerson, Keena, & O'Brien, 2012; Gallagher, Nordberg, & Dibley, 2019; Gallagher, Wahler, Minasian, & Edwards, 2019; Garcia, Kenyon, Brolan, Coughlin, & Guedes, 2019; Hamilton, 2019; Harrell, Cavanagh, & Roman, 1998; Kennedy-Hendricks, Bandara, Merritt, Barry, & Saloner, 2021; Kerr et al., 2011; Kouimtsidis, Reynolds, & Asamoah, 2007; Maddox, 2023; McIvor, 2009; McIvor et al., 2006; Moore, Barongi, & Rigg, 2017; Morse et al., 2014; Morse, Silverstein, Thomas, Bedel, & Cerulli, 2015; Murphy, 2011; Powell, 2012; Ricketts, Bliss, Murphy, & Brooker, 2005; Salzman, 2023; Schiff & Waegemakers Schiff, 2010)

## References

Bates, T. J. (2009). *Drug court: Breaking the black magic spell of drug addiction for women: A qualitative study.* (Doctor of Philosophy). The University of Utah, Dissertation Abstracts International Section A: Humanities and Social Sciences.

Bevli, S. (2018). *Effectiveness of the substance abuse and crime prevention act: the experiences of Hispanic residents.* (Doctor of Psychology). University of the Rockies, Dissertation Abstracts International: Section B: The Sciences and Engineering.

Datchi, C. C., & Ancis, J. R. (2017). Women and adult drug treatment courts: Surveillance, social conformity, and the exercise of agency. In J. R. Ancis (Ed.), *Gender, psychology, and justice: The mental health of women and girls in the legal system* (pp. 101-126). New York, NY: New York University Press; US.

Dickson-Gomez, J., Spector, A., Krechel, S., Li, J., Montaque, H. D. G., Ohlrich, J., . . . Weeks, M. (2022). Barriers to drug treatment in police diversion programs and drug courts: A qualitative analysis. *Am J Orthopsychiatry, 92*(6), 692-701. doi:10.1037/ort0000643

10.1037/ort0000643. Epub 2022 Oct 13.

Eley, S., Malloch, M., McIvor, G., Yates, R., & Brown, A. (2002). *The Glasgow drug court in action: the first six months*. Retrieved from Scotland:

Fischer, M., Geiger, B., & Hughes, M. E. (2007). Female recidivists speak about their experience in drug court while engaging in appreciative inquiry. *International Journal of Offender Therapy & Comparative Criminology, 51*(6), 703-722. doi:10.1177/0306624X07299304

10.1177/0306624X07299304. Epub 2007 Jul 5.

Francis, T. R., & Abel, E. M. (2014). Redefining success: A qualitative investigation of therapeutic outcomes for noncompleting drug court clients. *Journal of Social Service Research, 40*(3), 325-338. doi:10.1080/01488376.2013.875094

Fulkerson, A., Keena, L. D., & O'Brien, E. (2012). Understanding success and nonsuccess in the drug court. *International Journal of Offender Therapy & Comparative Criminology, 57*(10), 1297-1316. doi:10.1177/0306624X12447774

10.1177/0306624X12447774. Epub 2012 May 28.

Gallagher, J. R., Nordberg, A., & Dibley, A. R. (2019). Improving graduation rates for African Americans in drug court: Importance of human relationships and barriers to gaining and sustaining employment. *J Ethn Subst Abuse, 18*(3), 387-401. doi:10.1080/15332640.2017.1381661

10.1080/15332640.2017.1381661. Epub 2017 Nov 16.

Gallagher, J. R., Wahler, E. A., Minasian, R. M., & Edwards, A. (2019). Treating opioid use disorders in drug court: participants’ views on using medication-assisted treatments (MATs) to support recovery. *International Criminal Justice Review, 29*(3), 249-261. doi:10.1177/1057567719846227

Garcia, R. A., Kenyon, K. H., Brolan, C. E., Coughlin, J., & Guedes, D. D. (2019). Court as a health intervention to advance Canada's achievement of the sustainable development goals : a multi-pronged analysis of Vancouver's Downtown Community Court. *Global Health, 15*(1), 80. doi:10.1186/s12992-019-0511-9

10.1186/s12992-019-0511-9.

Hamilton, L. (2019). *Health-related quality of life among community-based offenders: How 'well-being' affects substance abuse treatment engagement.* (Doctor of Philosophy). Temple University, Dissertation Abstracts International Section A: Humanities and Social Sciences.

Harrell, A., Cavanagh, S., & Roman, J. (1998). *Findings from the evaluation of the D.C. Superior Court drug intervention program*. Retrieved from US:

Kennedy-Hendricks, A., Bandara, S., Merritt, S., Barry, C. L., & Saloner, B. (2021). Structural and organizational factors shaping access to medication treatment for opioid use disorder in community supervision. *Drug Alcohol Depend, 226*, 108881. doi:10.1016/j.drugalcdep.2021.108881

10.1016/j.drugalcdep.2021.108881. Epub 2021 Jun 26.

Kerr, J., Tompkins, C., Tomaszewski, W., Dickens, S., Grimshaw, R., Wright, N., & Barnard, M. (2011). *The dedicated drug courts pilot evaluation process study*. Retrieved from Ministry of Justice, UK: [www.justice.gov.uk/publications/research.htm](https://stir-my.sharepoint.com/personal/eff2_stir_ac_uk/Documents/NESSIE%20NIHR%20Evidence%20Synthesis%20Group%20Todhunter%202022/Treatment%20order%20review%202023/TO%20Qual%20Review%20Article/www.justice.gov.uk/publications/research.htm)

Kouimtsidis, C., Reynolds, M., & Asamoah, V. (2007). Treatment or prison: service user and staff experiences of drug treatment and testing orders. *Psychiatric Bulletin, 31*(12), 463-466. doi:10.1192/pb.bp.107.014548

Maddox, M. E. (2023). *The effectiveness of drug treatment court: Participants' recommendations for improvement of the drug treatment court diversion program.* (Doctor in Psychology). William James College, Dissertation Abstracts International: Section B: The Sciences and Engineering.

McIvor, G. (2009). Therapeutic jurisprudence and procedural justice in Scottish Drug Courts. *Criminology & Criminal Justice, 9*(1), 29–49. doi:10.1177/1748895808099179

McIvor, G., Barnsdale, L., Eley, S., Malloch, M., Yates, R., & Brown, A. (2006). *The operation and effectiveness of the Scottish drug court pilots*. Retrieved from Scotland:

Moore, K. A., Barongi, M. M., & Rigg, K. K. (2017). The Experiences of Young Adult Offenders Who Completed a Drug Court Treatment Program. *Qual Health Res, 27*(5), 750-758. doi:10.1177/1049732316645782

10.1177/1049732316645782. Epub 2016 Jul 10.

Morse, D. S., Cerulli, C., Bedell, P., Wilson, J. L., Thomas, K., Mittal, M., . . . Chin, N. (2014). Meeting health and psychological needs of women in drug treatment court. *J Subst Abuse Treat, 46*(2), 150-157. doi:10.1016/j.jsat.2013.08.017

10.1016/j.jsat.2013.08.017. Epub 2013 Sep 24.

Morse, D. S., Silverstein, J., Thomas, K., Bedel, P., & Cerulli, C. (2015). Finding the loopholes: a cross-sectional qualitative study of systemic barriers to treatment access for women drug court participants. *Health & Justice, 3*, 12. doi:10.1186/s40352-015-0026-2

10.1186/s40352-015-0026-2. Epub 2015 Jun 17.

Murphy, J. (2011). Drug court as both a legal and medical authority. *Deviant Behavior, 32*(3), 257-291.

Powell, C. L. (2012). *Coerced drug treatment in England and Wales: An evaluation of Drug Treatment and Testing Orders in one locality.* (PhD Psychology). University of Leicester, UK.

Ricketts, T., Bliss, P., Murphy, K., & Brooker, C. (2005). Engagement with drug treatment and testing orders: A qualitative study. *Addiction Research & Theory, 13*(1), 65-78. doi:10.1080/16066350512331328168

Salzman, H. J. (2023). *Motherhood and substance use: An examination of societal pressures in the motivation to complete court-ordered drug treatment and to desist from future criminal activity and drug use.* (Doctor of Philosophy). University of Manchester, Dissertation Abstracts International: Section B: The Sciences and Engineering, UK.

Schiff, R., & Waegemakers Schiff, J. (2010). Housing needs and preferences of relatively homeless Aboriginal women with addiction. *Social Development Issues, 32*(3), 65-76.
