# Supplementary material for: Understanding the health and well-being impacts and implementation barriers and facilitators of legally-mandated non-custodial drug and alcohol treatment for justice-involved adults: a qualitative evidence synthesis
Source: Health Justice. 2025 Oct 1;13:58. doi: 10.1186/s40352-025-00361-5 (PMC12487214; doi:10.1186/s40352-025-00361-5)
Supplement: Supplementary file 14 — Additional file 14. Reflexivity. Description of data: details of the authors’ backgrounds, expertise and assumptions relevant to the review [file 40352_2025_361_MOESM14_ESM.docx]

# Additional file 14. Reflexivity

The core team (anonymised for review) has varied professional and academic backgrounds including psychology (anonymised for review), development of qualitative evidence synthesis methodology (anonymised for review), information specialist/systematic review methodologist (anonymised for review), Cochrane systematic reviews (all), health professions (anonymised for review) and health and social care services research (all).

Some of the core team have personal experience of substance use problems through affected family members; for confidentiality and anonymity we have chosen not to disclose which team members. Members of the core team believe that people with substance use problems have a fundamental right to compassionate treatment, others have no strong views on the use of mandatory treatment orders. Core team members have not published any eligible studies and so there was a low risk of biased appraisal when assessing study methodological limitations. The core team had no preconceptions of what the findings of our reviews might reveal. The review process and progress were regularly assessed and discussed between the review authors, topic experts (anonymised for review) and PPI and stakeholder contributors.

Our review topic experts (anonymised for review) have expertise in drug and alcohol research, occupational therapy and mental health research (anonymised for review) and research with people involved in the criminal justice system and have authored several publications on these topics. Our PPI lead has expertise in public health relating to alcohol and drug use (anonymised for review). Engagement with our wider PPI and stakeholder contributor group throughout the review has contributed further topic expertise and minimised the risk of our preconceptions and backgrounds influencing our selection of qualitative studies and analysis and the interpretation of the findings.

To improve the transparency of this review our team undertook an Equality, Diversity and Inclusion evaluation for systematic reviews prior to running the literature searches (Centre for Ethnic Health Research, 2023). Available on request from the lead author.

## Reference

Centre for Ethnic Health Research. (2023). Equality impact assessment (EqIA) form for systematic reviews. Retrieved from <https://arc-em.nihr.ac.uk/clahrcs-store/equality-impact-assessment-eqia-toolkit>
