# Supplementary material for: Understanding the health and well-being impacts and implementation barriers and facilitators of legally-mandated non-custodial drug and alcohol treatment for justice-involved adults: a qualitative evidence synthesis
Source: Health Justice. 2025 Oct 1;13:58. doi: 10.1186/s40352-025-00361-5 (PMC12487214; doi:10.1186/s40352-025-00361-5)
Supplement: Supplementary file 15 — Additional file 15. CRediT author Statement. Description of data: table describing author contributions in the form of the CRediT statement [file 40352_2025_361_MOESM15_ESM.docx]

## Additional file 15. CRediT author Statement

| **Term** | **Definition** | **Authors contributing** |
| --- | --- | --- |
| Conceptualization | Ideas; formulation or evolution of overarching research goals and aims | Anonymised for review |
| Methodology | Development or design of methodology; creation of models |  |
| Software | Programming, software development; designing computer programs; implementation of the computer code and supporting algorithms; testing of existing code components; designing of search strategies |  |
| Validation | Verification, whether as a part of the activity or separate, of the overall replication/ reproducibility of results/experiments and other research outputs |  |
| Formal analysis | Application of statistical, mathematical, computational, or other formal techniques to analyze or synthesize study data |  |
| Investigation | Conducting a research and investigation process, specifically performing the experiments, or data/evidence collection |  |
| Resources | Provision of study materials, reagents, materials, patients, laboratory samples, animals, instrumentation, computing resources, or other analysis tools |  |
| Data Curation | Management activities to annotate (produce metadata), scrub data and maintain research data (including software code, where it is necessary for interpreting the data itself) for initial use and later reuse |  |
| Writing - Original Draft | Preparation, creation and/or presentation of the published work, specifically writing the initial draft (including substantive translation) |  |
| Writing - Review & Editing | Preparation, creation and/or presentation of the published work by those from the original research group, specifically critical review, commentary or revision – including pre-or postpublication stages |  |
| Visualization | Preparation, creation and/or presentation of the published work, specifically visualization/ data presentation |  |
| Supervision | Oversight and leadership responsibility for the research activity planning and execution, including mentorship external to the core team |  |
| Project administration | Management and coordination responsibility for the research activity planning and execution |  |
| Funding acquisition | Acquisition of the financial support for the project leading to this publication | Not applicable |
